# An fNIRS-based investigation of visual merchandising displays for fashion stores

**DOI:** 10.1371/journal.pone.0208843

**Published:** 2018-12-11

**Authors:** Xiaolong Liu, Chang-Seok Kim, Keum-Shik Hong

**Affiliations:** 1 School of Mechanical Engineering, Pusan National University, Geumjeong-gu, Busan, Republic of Korea; 2 School of Life Science and Technology, University of Electronic Science and Technology of China, West Hi-Tech Zone, Chengdu, Sichuan, P. R. China; 3 Department of Cogno-Mechatronics Engineering, Pusan National University, Geumjeong-gu, Busan, Republic of Korea; Tohoku University, JAPAN

## Abstract

This paper investigates a brain-based approach for visual merchandising display (VMD) in fashion stores. In marketing, VMD has become a research topic of interest. However, VMD research using brain activation information is rare. We examine the hemodynamic responses (HRs) in the prefrontal cortex (PFC) using functional near-infrared spectroscopy (fNIRS) while positive/negative displays of four stores (menswear, womenswear, underwear, and sportswear) are shown to 20 subjects. As features for classifying the HRs, the mean, variance, peak, skewness, kurtosis, *t-*value, and slope of the signals for a 20-sec time window for the activated channels are analyzed. Linear discriminant analysis is used for classifying two-class (positive and negative displays) and four-class (four fashion stores) models. PFC brain activation maps based on *t*-values depicting the data from the 16 channels are provided. In the two-class classification, the underwear store had the highest average classification result of 67.04%, whereas the menswear store had the lowest value of 64.15%. Men’s classification accuracy for the underwear stores with positive and negative displays was 71.38%, whereas the highest classification accuracy obtained by women for womenswear stores was 73%. The average accuracy over the 20 subjects for positive displays was 50.68%, while that of negative displays was 51.07%. Therefore, these findings suggest that human brain activation is involved in the evaluation of the fashion store displays. It is concluded that fNIRS can be used as a brain-based tool in the evaluation of fashion stores in a daily life environment.

## Introduction

Customer behavior due to marketing is strongly influenced by the perception of the surrounding environment as shown by dandyism [1]. Visual merchandising display (VMD) is a marketing strategy to develop floor plans and displays to attract, engage, and stimulate the customer towards making a purchase. If done effectively, the marketers are able to maximize sales. It can also help marketers develop a unique marketing identity and brand, differentiating themselves in the market [2]. In this paper, by using functional near-infrared spectroscopy (fNIRS), we investigate the brain activation of potential customers when viewing various VMDs of four fashion stores.

From the early twenty-first century, VMD has become a science and one of the main tools for increasing sales. A good display can attract the consumer’s attention, which is essential in the decision-making process leading to a purchase. To capture a consumer’s mind, the needs of the consumer when viewing the VMD should be considered. Law et al. [3] investigated, from a psychological perspective, the affective responses of the consumers to visual stimuli in stores by considering the aesthetic and symbolic aspects of function-oriented products. The conformity between the preconceived fashion image and the image projected from a fashion store works as a mediator that affects the actual purchase decision. Shin et al. [4] investigated the benefits of eco-friendly VMDs on satisfaction as well as the effect of satisfaction on store displays. They demonstrated experimental evidence that psychological and social satisfaction play a fundamental role in the benefit of eco-friendly VMDs leading to positive store characteristics. Such results indicate that social information results in positive psychological and emotional satisfaction, which consequently leads to a positive view of the store for retail customers.

Currently, VMD studies of the influence of brain activity from a visual stimulus has become an important research issue. To investigate the activated brain region when viewing positive and negative stimuli, Zhang et al. [5] used functional magnetic resonance imaging (fMRI) and reported that the local connections in the brain increased while viewing affective pictures. In the positive emotional network, a positive affect score was correlated with the betweenness value of the right orbital frontal cortex (OFC) and the right putamen, while in the negative emotional network, a negative affect score was correlated with the betweenness value of the left OFC and the left amygdala. Local efficiencies in the left superior and inferior parietal lobes were correlated with the subsequent arousal ratings of the positive and negative pictures, respectively. Metereau et al. [6] found that the activities in the medial OFC and the ventromedial prefrontal cortex (PFC) were correlated positively in terms of the expected values of the cues in anticipation of rewards and punishments. This finding indicates that the medial OFC and the ventromedial PFC encode a generally unsigned anticipatory value signal, regardless of the valence of anticipation (positive/negative) and type (appetitive/aversive events). Bercik et al. [7] investigated the impact of color illumination (an essential factor in food shoppers’ perception) using EEG. Schmeichel et al. [8] found that exercising (vs. not exercising) self-control increases left frontal cortical activity during picture viewing, particularly among individuals with a relatively more active behavioral activation system than behavioral inhibition system, and particularly when viewing positive pictures. A similar but weaker pattern emerged when viewing negative pictures. Using fNIRS to measure the brain hemodynamic responses (HRs), the finding from Kreplin et al. [9] indicated that the right PFC was involved in positive evaluation of visual art that may be related to the judgment of pleasantness or attraction. This result suggests that emotional processing takes precedence over digressing viewing discipline during visual deliberation of art. In this case, the emotional nodule was brought to the beginning because of the participant's search for a personal association with the art images. Hence, participants relied on their own judgement and personal relationship with the art to make a subjective evaluation of the price.

In this work, fNIRS, an optical imaging method [10–17] is used to obtain brain signals. fNIRS offers several advantages over other modalities, such as being non-invasive, portable, no-noise, harmless, and inexpensive [18–22]. fNIRS can measure the light intensity changes in near-infrared lights of wavelength between 650 nm and 1,000 nm [23] and monitors the variation in regional cerebral blood flow. These changes are caused by concentration variations in oxygenated hemoglobin (HbO) and deoxygenated hemoglobin (HbR) from neural activity. The principles and practical applications of fNIRS were described in detail previously [10].

In this paper, the HRs evoked by viewing positive and negative pictures of four different store categories (menswear store, womenswear store, underwear store, and sportswear store) are measured. The obtained results are portrayed in brain activation maps using the *t*-values of the measured HRs and the desired HRs [24]. Two-class and four-class linear discriminate analysis (LDA) [25] classifiers are used to distinguish the HRs from different picture stimuli, for which seven features (mean, peak, variance, skewness, kurtosis, *t*-value, and slope) are utilized.

## Materials and methods

### Ethics statement

The experiment was conducted upon the approval of the Pusan National University Institutional Review Board. Written consent was obtained from all subjects prior to starting. The experimental procedure was conducted in accordance with the ethical standards described in the latest Declaration of Helsinki.

### Participants

Twenty healthy subjects (age 28 ± 3, all right handedness, 10 males and 10 females) participated in this experiment. All of them were healthy with no previous history of neurological disorders. All participants were informed about the procedure and operation of the fNIRS system prior to providing written consent. They were asked to avoid body motion and remain relaxed during the experiment. When viewing the stimuli, the subjects were asked to focus on each picture presented on the computer screen and indicate either like or dislike.

### Visual stimuli

**Fig 1:**
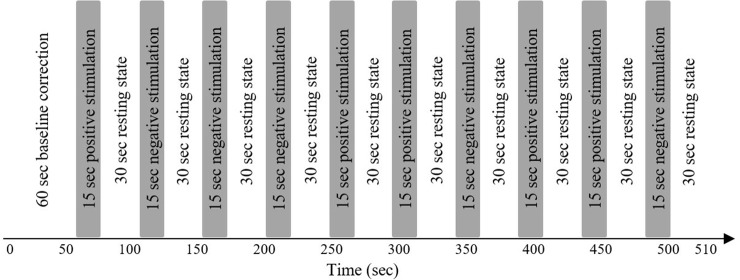
The experimental paradigm for ten trials of positive and negative store images: One trial includes a 15-sec stimulus followed by a 30-sec rest, and then a 60-sec baseline correction is performed followed by five trials of positive/negative images are randomly exhibited.

The visual stimuli were projected onto a monitor screen (Samsung LED Model: LS24A300) in front of the participants. The viewing distance to the screen was approximately 50 cm. The size of screen is 23 inch (16:9) and the resolution is 1,980 × 1,080 pixels. Four categories of fashion stores (menswear, womenswear, underwear, and sportswear) were shown on the screen to prompt negative and positive images. One trial consists of a 15 sec picture stimulus followed by a 30 sec rest for individual categories as shown in Fig 1. One session includes an initial 60 sec baseline correction period and a 450 sec task period (5 trials of positive and negative images in random order). The entire experiment for 4 categories takes a total of 2,040 sec.

### Stimulation picture selection

**Fig 2:**
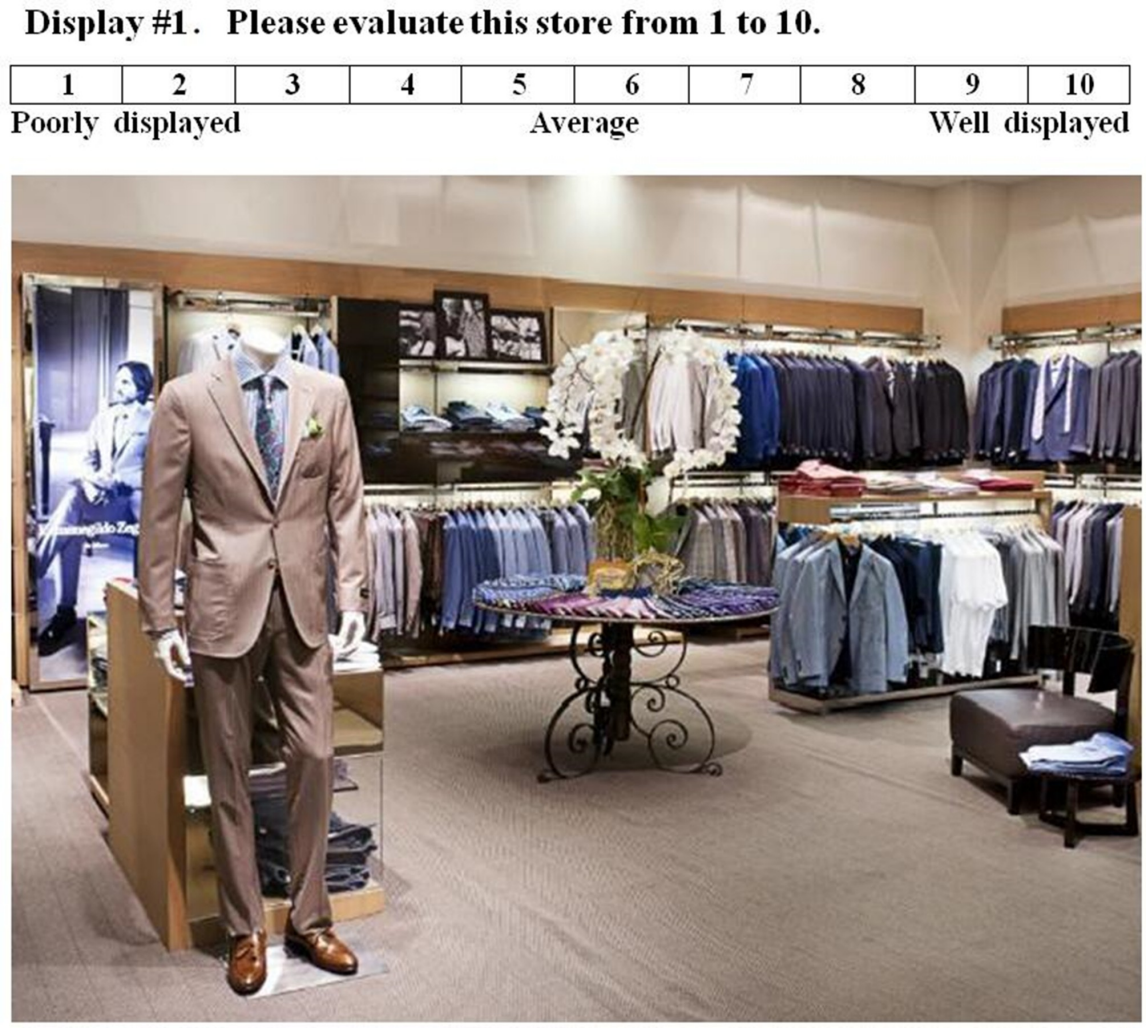
The example of the survey.

**Fig 3:**
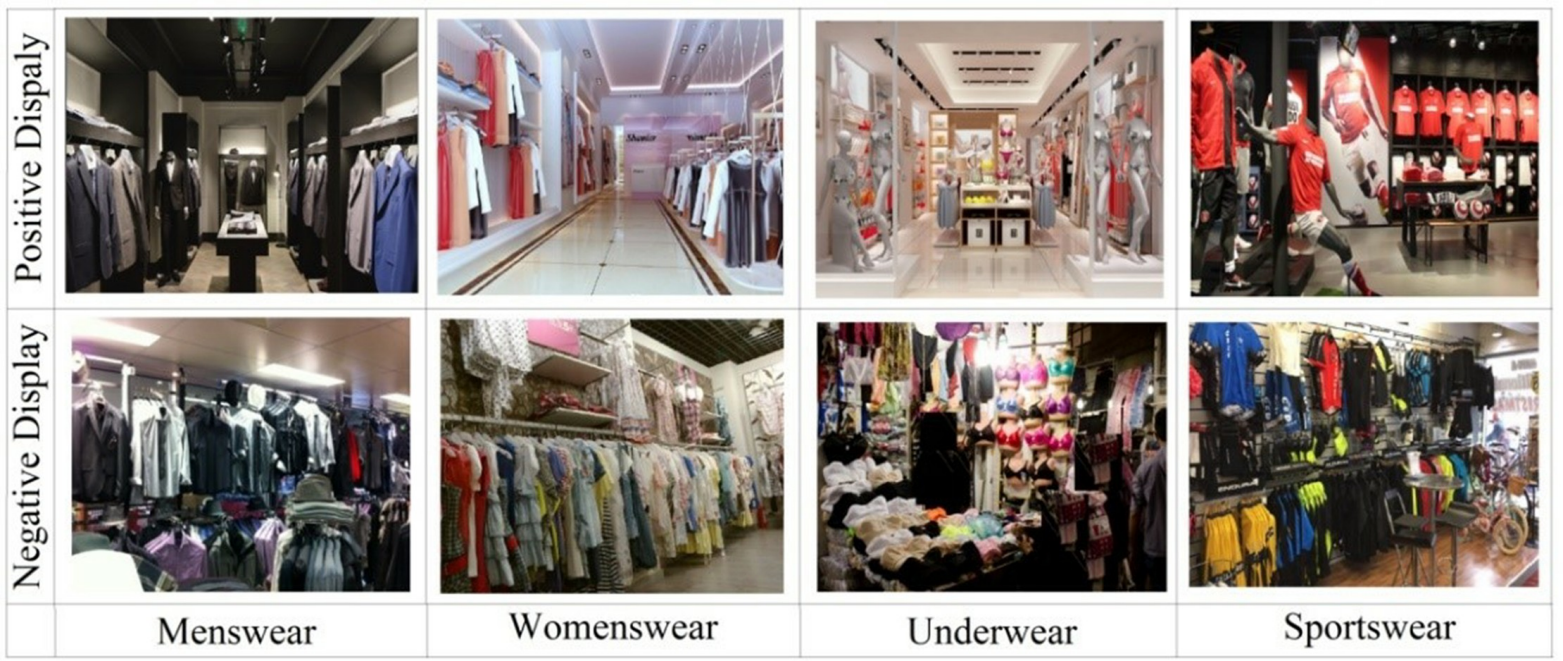
Examples of positive and negative displays: Four categories.

The pictures used in this work were initially attained from the Internet (from a collection of standardized photographic materials) by typing “menswear store display,” “womenswear store display,” “underwear store display,” and “sportswear store display” into http://google.com [26, 27]. Only those pictures with good resolution were first screened. To narrow down the number of pictures to be used for experiment, an online survey was performed, in which two hundred sixteen people participated. In this survey, students and workers (all participants are adults) with different majors and positions were invited, yielding a total of 216 answers. In evaluating the quality of the pictures, a 10 points scale was used (10 means the most positive, and 1 means the most negative), see Fig 2 for instance. Lastly, 10 pictures (5 positive, 5 negative) were selected for each category, which resulted in a total of 40 pictures for 4 categories (menswear, womenswear, underwear, sportswear). It is noted that the scores of the selected 5 positive pictures ranged from 8 to 10 on average, and those of the 5 negative pictures ranged from 1 to 3. It is also remarked that four out of 10 underwear pictures were from men’s, and six out of 10 sportwear pictures were from women’s. Fig 3 shows typical examples of positive and negative images from the four categories.

### Signal acquisition and processing

**Fig 4:**
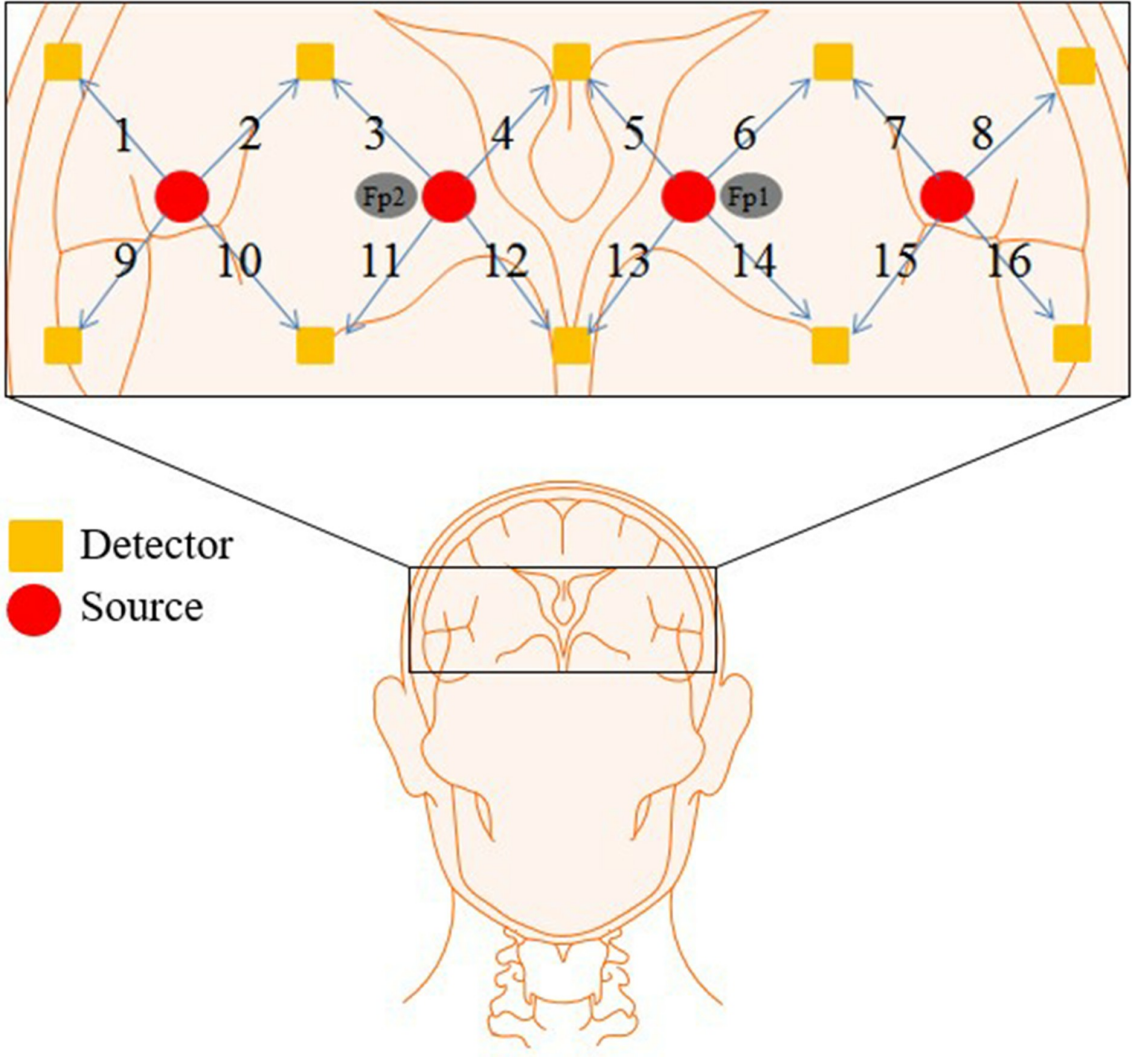
Optode arrangement in the prefrontal cortex: Squares and circles are the detectors and emitters, respectively, and Fp1 and Fp2 denote the reference points from the International 10–20 System.

To measure brain activity, a multi-channel continuous fNIRS imaging system (DYNOT: Dynamic Near-infrared Optical Tomography; two wavelengths: 760 and 830 nm; NIRx Medical Technologies, NY) with a sampling rate of 1.81 Hz was used. A 16-channel probe configuration was placed on the prefrontal cortex [28–30] aligned to the locations of Fp1 and Fp2 in the International 10–20 System as shown in Fig 4.

To investigate the fNIRS data, the open source software NIRS-SPM [31] was used together with our own MATLAB codes (Mathworks, USA). The obtained intensity signals of near-infrared lights were first converted to HbO and HbR concentration changes using the modified Beer-Lamberts law.

$$\left[\begin{array}{l} \Delta C_{\mathrm{HbO}(t)}\\ \Delta C_{\mathrm{HbR}(t)} \end{array}\right]={\left[\begin{array}{l} \alpha_{\mathrm{HbO}}(\lambda_{1})\;\alpha_{\mathrm{HbO}}(\lambda_{2})\\ \alpha_{\mathrm{HbR}}(\lambda_{1})\;\alpha_{\mathrm{HbR}}(\lambda_{2}) \end{array}\right]}^{-1}\left[\begin{array}{l} \Delta A(t,\lambda_{1})\\ \Delta A(t,\lambda_{2}) \end{array}\right]\frac{1}{l\cdot d},$$ (1)

where Δ*A* denotes the variation in the optical density of the near-infrared light emitted at wavelength *λ*_*j*_, *α*_HbX_(*λ*_1_) refers to the extinction coefficient of HbX in μMmm^−1^, *d* denotes the differential path length factor (DPF) that is unitless, and *l* indicates the emitter-detector distance (in mm). To remove the physiological noise related to respiration and cardiac signals, a 4th order Butterworth low-pass filter with a cutoff frequency of 0.15 Hz was used [32]. Additionally, to eliminate drift in the signal, de-trending was carried out inside the NIRS-SPM software [33].

### Regions of interest

To visualize the brain activity from fNIRS data, *t-*value maps are the most intuitive method. In this paper, the *t*-values were computed by using the *robustfit* function available in Matlab^TM^, which is an iterative reweighted least squares estimation algorithm with a bisquare weighting function. Let $y_{i}^{j}\in R^{N\times 1}$ be the measured data at the *i*-th channel for the *j*-th stimulus (i.e., 10 stimuli for one category), and let *N* be the data size per stimulus (in this study, *N* = 64 since 15 sec stimulus and 20 sec rest were adopted using a sampling frequency of 1.81 Hz). It is noted that out of the 30 sec rest period, only 20 sec data were used. Then, the linear regression model is defined as follows [24].

$$y_{i}^{j}=\beta_{i}^{j}X_{r}+\alpha_{i}^{j}\cdot 1+\varepsilon_{i}^{j}$$ (2)

where **X**_r_∈*R*^*N*×1^ is the expected hemodynamic response upon viewing the given stimuli, **1**∈*R*^*N*×1^ is a column vector made of 1’s to correct the offset of the baseline, *β* is the unknown coefficient indicating the activity strength of the expected hemodynamic response, and **ε**∈*R*^*N*×1^ denotes white Gaussian noise. The following equations describe the steps to obtain the *t*-values.

$$[{\hat{\beta }}_{i}^{j},\mathrm{stats}]=robustfit(X_{r},y_{i}^{j}),$$ (3)

$$t_{i}^{j}=\frac{{\hat{\beta }}_{i}^{j}}{SE({\hat{\beta }}_{i}^{j})},$$ (4)

where ${\hat{\beta }}_{i}^{j}$ indicates the estimate of $\beta_{i}^{j}$ and stats refers to the statistical data including *t*-value, *p*-value, standard error (SE) of the estimated coefficient.

The brain activity-related coefficient was related to the *t*-value to determine if its value was statistically greater than the *t*-critical (*t*_crt_) value. The *t*_crt_ value depends on the degrees of freedom (i.e., 63, which is *N*—1), and it is 1.67 in this case. If the shape of the HbO response is closer to that of the expected HR (**y**_r_), the *t*-value is high. If the HbO response is different, the *t*-value is low (or negative). If a channel with the computed *t*-value is greater than *t*_crt_, the channel is considered active. After obtaining all the *t*-maps from the 4 categories, the regions of interest (ROIs) [34, 35] for individual categories are investigated through the maps. The ROI denotes the area where the *t*-value(s) are higher than *t*_crt_.

**Fig 5:**
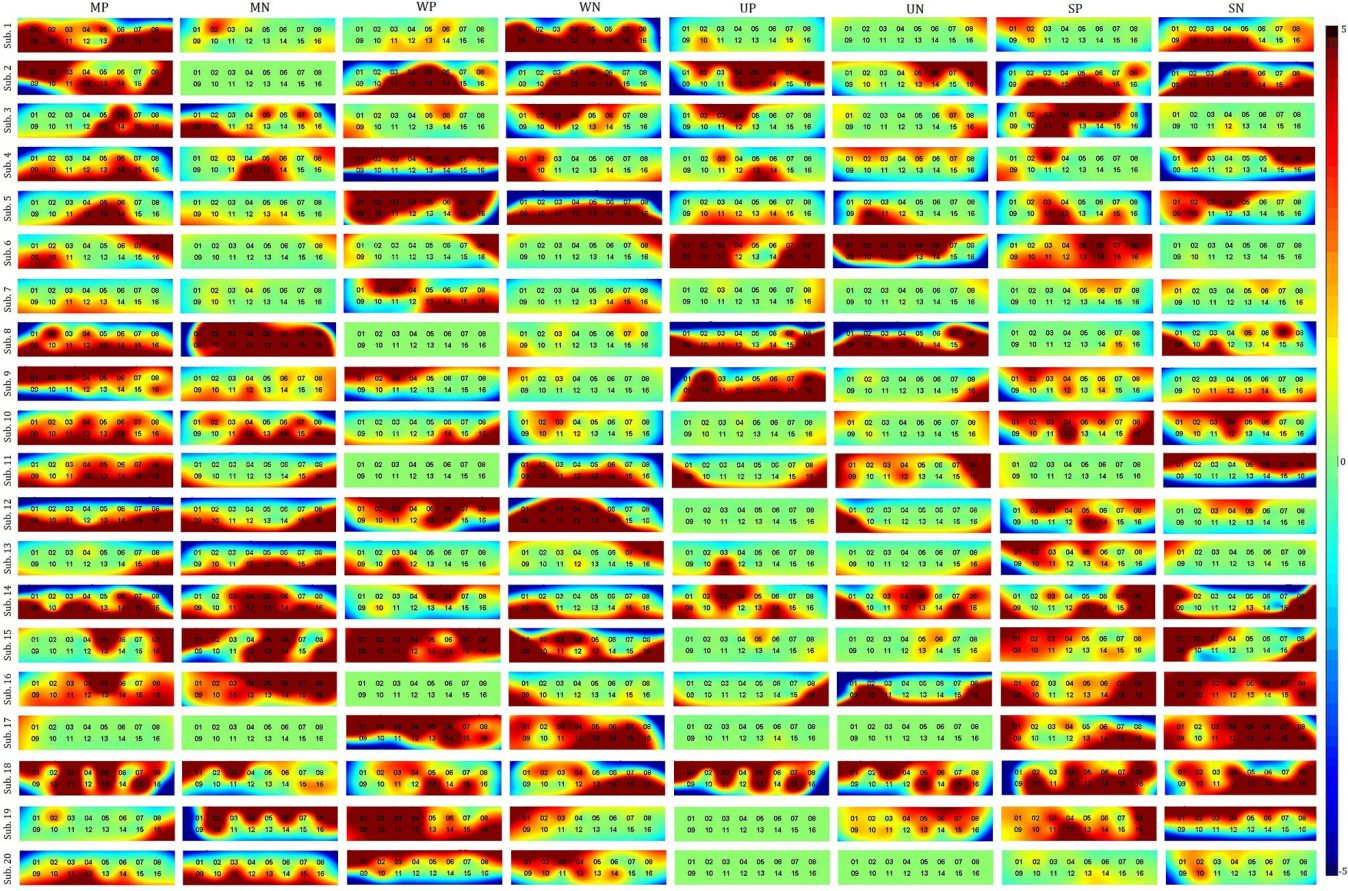
Brain activation maps (averaged over 5 trials) from 20 subjects based on *t*-values: Positive and negative displays for the four categories (MP: Menswear positive, MN: Menswear negative, WP: Womenswear positive, WN: Womenswear negative, UP: Underwear positive, UN: Underwear negative, SP: Sportswear positive, SN: Sportswear negative).

**Fig 6:**
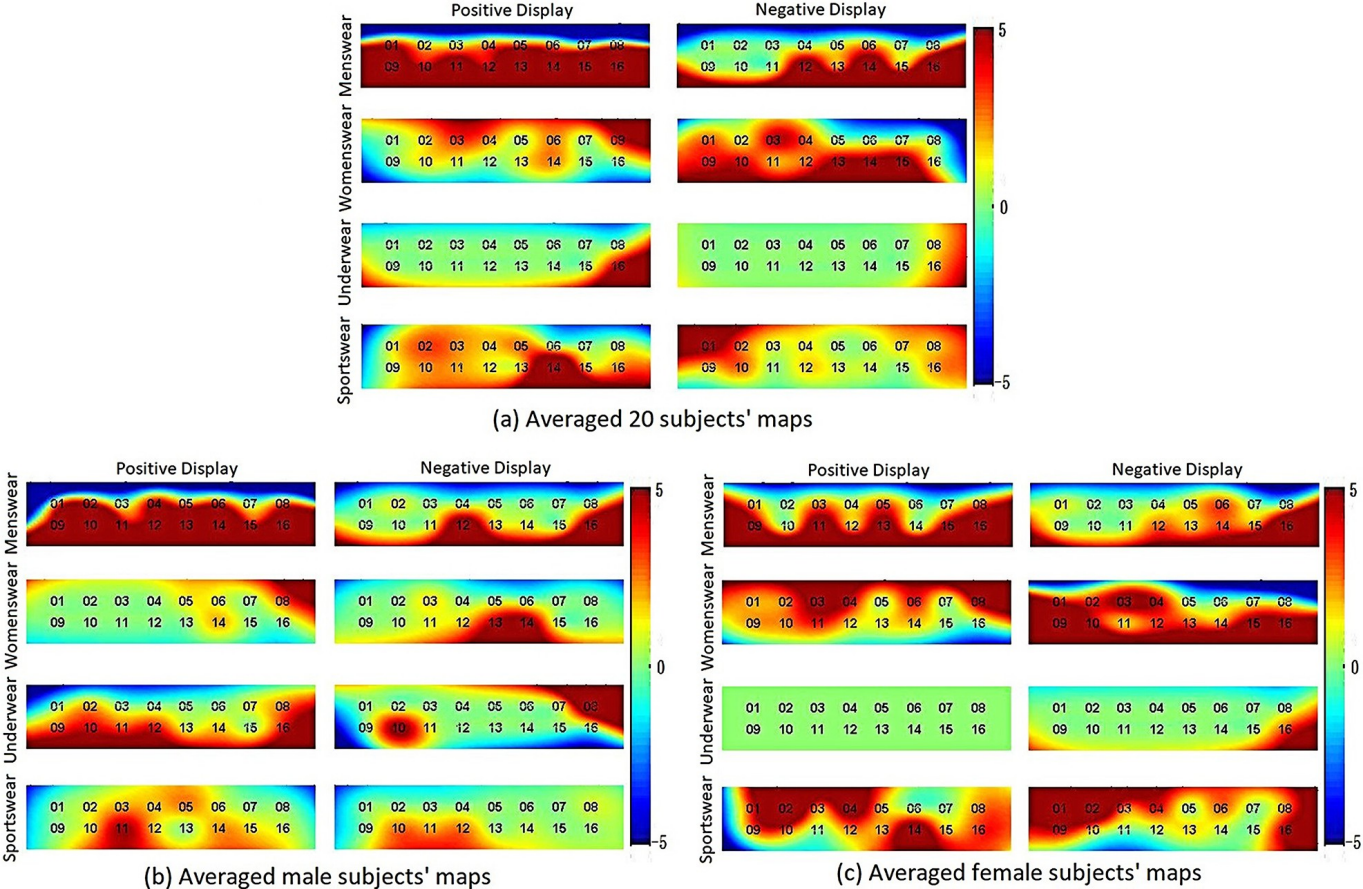
The averaged maps (over 20 subjects) for 4 categories of positive and negative displays.

Fig 5 depicts the averaged brain activation maps over five trials for 20 subjects, in which MP, MN, WP, WN, UP, UN, SP, and SN stand for menswear positive, menswear negative, womenswear positive, womenswear negative, underwear positive, underwear negative, sportswear positive, and sportswear negative, respectively. Fig 6A compares the positive and negative maps averaged over all 20 subjects, Fig 6B contrasts those averaged over ten male subjects, and Fig 6C analyzes those averaged over 10 female subjects. As seen in Fig 6A, 6B and 6C, the ROIs when viewing positive/negative displays from the four categories are clearly different. For further analysis of feature selection and classification, only the active channels from each ROI were used because using the signals only from the associated ROI (rather than using the data from all channels) results in better classification accuracy [36, 37].

### Feature extraction and classification

**Fig 7:**
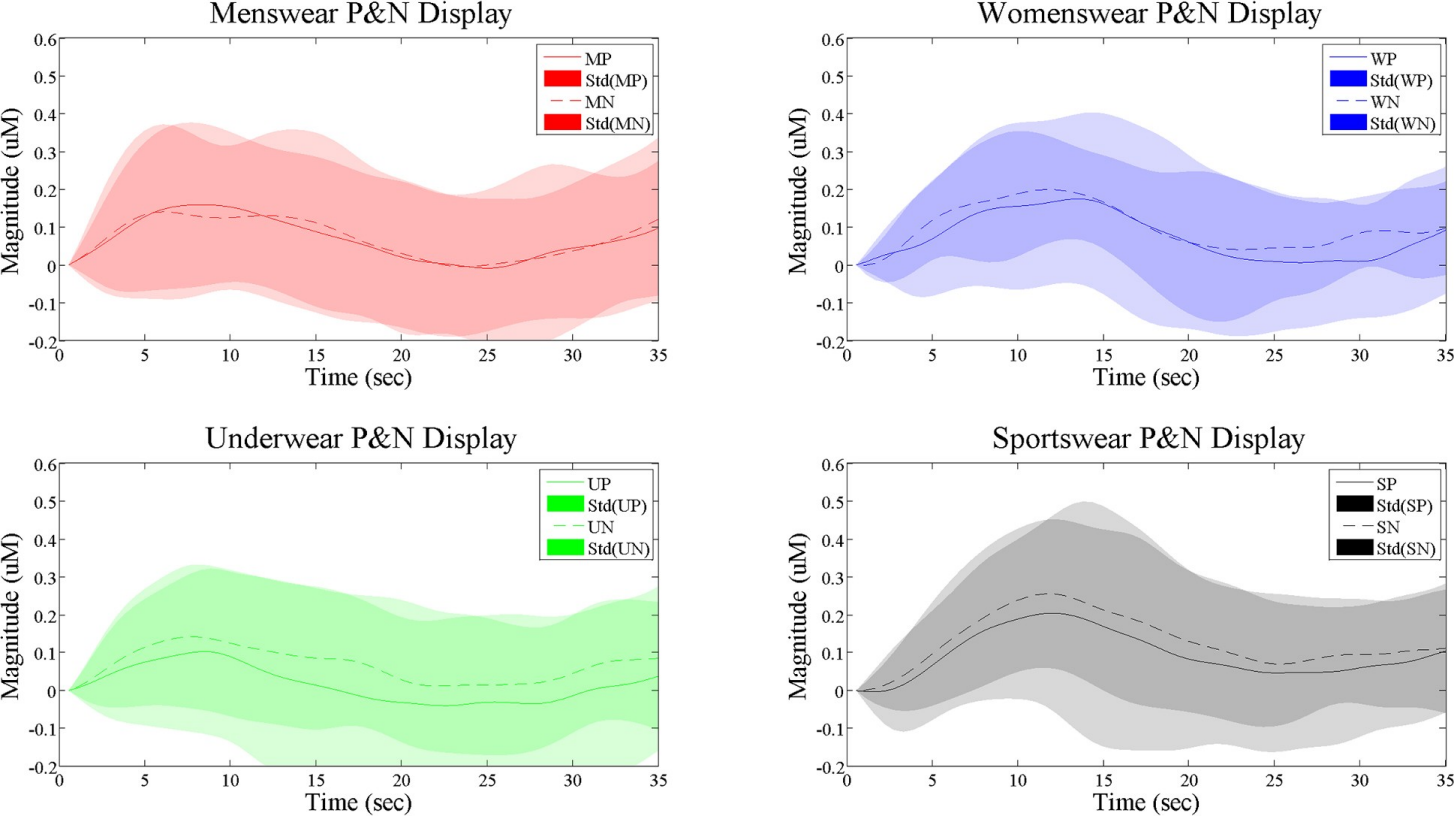
Comparison of the averaged HRs of positive and negative displays (the *x*-axis indicates the time series, in which 0–15 sec is the stimulus period and 16–35 sec is the rest period; the *y*-axis indicates the magnitude of HbO).

**Fig 8:**
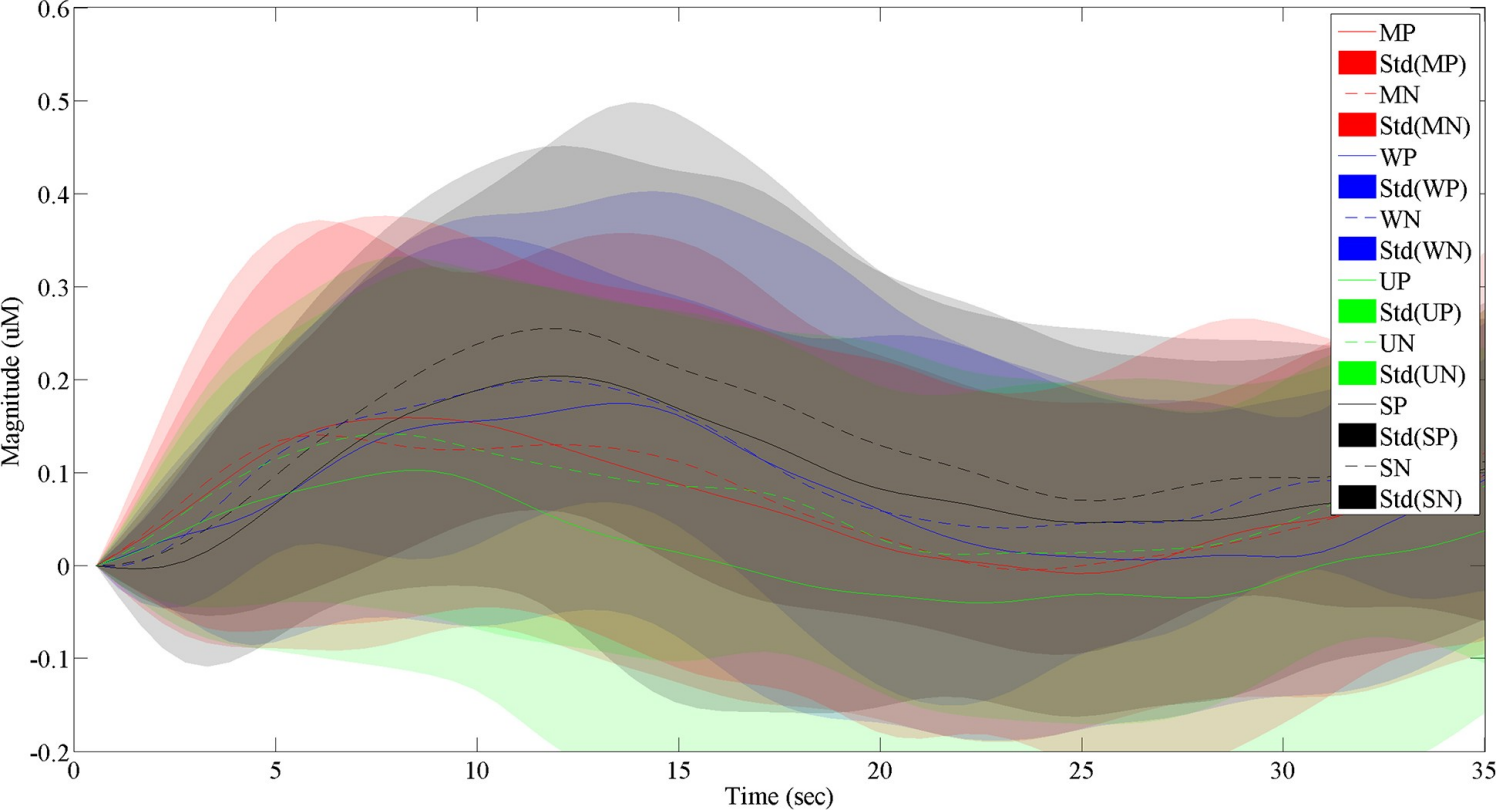
Comparison of the averaged HRs (over 5 trials and 20 subjects) of positive and negative displays for the 4 categories.

In this study, the averaged HbO signals from the active channels were considered for classification [38, 39] (see Fig 7 and Fig 8). For features, the following seven statistical signal properties (mean, peak, variance, skewness, kurtosis, *t*-value, and slope) were considered [22, 40].

- Mean: the average signal value (for 20 sec from the start of the display),
- Peak: the maximum value of the selected window (for 20 sec),
- Variance: a measure of the signal spread (for 20 sec),
- Skewness: a measure of the asymmetry of the signal around its mean relative to a normal distribution,
- Kurtosis: a measure for the sharpness of the peak relative to a normal distribution,
- *t*-value: a measure of a test statistic for the difference between the dataset and the expected HR,
- Slope: the value that fits a regression line to the given data set.

To enhance the classification accuracy, a 20 sec window signal was used rather than using the entire 45 sec task-rest period. The previous study [41] used a 2–7 sec time window for a 10 sec stimulus for classification purposes of brain-computer interface (BCI) based on two features: mean and slope. However, in this study, we applied a different stimulus period (15s) with different features selection. Therefore, a 20 sec time window is selected for further classification. Additionally, the feature values have been rescaled between 0 and 1 using the following equation.

$$z^{\prime }=\frac{z-\min z}{\max\;z‑\min\;z},$$ (5)

where *z*∈*R*^*n*^, *z*′ is the rescaled value between 0 and 1, min *z* is the smallest value, and max *z* is the largest value. For efficient classifications, several classifiers have been discussed in the literature [42–48]. Here, we used an LDA classifier to verify the HbO signals.

## Results

Fig 5 shows all brain activation maps from 20 participants for menswear, womenswear, underwear, and sportswear by projecting positive and negative pictures of store displays and whose data were obtained from the prefrontal cortex. There is some similarity across the subjects for a given category. Therefore, using the data from the ROI of each experiment, the averaged brain activation maps for the 20 subjects were obtained as shown in Fig 6A. In Figs 6B and 6C, the gender-based brain activation maps are drawn.

In the menswear experiment shown in Fig 6A, channels 1, 5, 8, 10, 11, 12, 13, 14, 15, and 16 were activated when viewing positive displays, and channels 12, 14, and 16 were activated when viewing negative displays. In the womenswear experiment, channels 3, 6, 8, and 14 were activated when viewing positive displays, and channels 1, 9, 10, 13, 14, and 15 were activated when viewing negative displays. In the underwear experiment, only channel 16 was activated when viewing both positive and negative displays. In the sportswear experiment, channels 2, 5, 10, 11, and 14 were activated when viewing positive displays, and channels 1, 2, 7, 12, and 16 were activated when viewing negative displays. Figs 6B and 5C present the male and female PFC activation maps. For male subjects, all channels with MP display stimulation were activated. Channel 8, 9, 10, 11, 12, 16 were activated by UP displays. In other stimulations, a few channels were activated (WP: channel 8, SP: channel 11, MN: channels 12 and 16, WN: channels 13 and 14, UN: channels 8 and 10, SN: channels 10 and 12). For female subjects, MP, WP, WN, and SP showed a large number of activated channels. Conversely, no channels were activated in UP, and only channel 16 was activated in UN. Based on averaging the activated channels, either among all subjects or by gender, through the intuitive observation a significant asymmetry of the *t*-maps is shown in the prefrontal cortex.

Fig 7 compares the average HbO signals over time and their standard deviations over 20 participants for the four categories. The following findings can be summarized as follows. i) For menswear, the peak HbO values from positive stimuli were higher than those of negative stimuli. ii) In contrast, the peak value of positive displays of underwear and sportswear were lower than that of negative displays. iii) However, peak values of positive/negative displays of womenswear were not distinguishable. Overall, out of four categories, the negative sportswear display was the most influential to the customer, showing the highest HbO response at its peak value. Fig 8 compares all the HbO responses of positive/negative displays from the four categories. We conclude that the order of hemodynamic activation levels for positive displays are sportswear, womenswear, menswear, and underwear.

Table 1 shows the two-class classification accuracies of all feature combinations (averaged over 20 subjects). In individual positive and negative classifications, the highest accuracies are marked in bold (blue): 64.15% (menswear), 64.95% (womenswear), 69.12% (underwear), and 66.27% (sportswear). Based on these best accuracies, Table 2 lists the subject-wise results of two-class classifications between positive and negative displays, in which good/bad looking underwear stores were classified most accurately, at 67.04%, and menswear stores were classified least accurately at 64.15%. Table 3 compares the four-class classification accuracies based on the average of 20 subjects with all feature combinations. In classifying four-store positive displays, the highest accuracy was 50.70%. In the case of negative displays, the highest accuracy was 51.10%. Table 4 shows the subject-wise four-class classification accuracies. The accuracy averaged across 20 subjects for the four positive displays was 50.68% (Sub.10 shows the highest accuracy of 76.67%). In the case of negative displays, the averaged accuracy was 51.07% (Sub.7 had 66.95%). Table 5 shows the gender-based two-class and four-class classification accuracies. For male subjects, the best classification category was underwear (71.38%), and the classification accuracy for four-class positive displays was 51.69%. The order of classification accuracies was underwear (71.38%), sportswear (68.33%), menswear (58.50%), and womenswear (p = 0.069 is considered to be not statistically significant). For female subjects, the womenswear display showed the best classification accuracy (73.00%). The order was womenswear (73.00%), menswear (69.81%), underwear (64.71%), and sportswear (61.70%). For four-store classification, negative displays had 52.34% classification accuracy, and positive displays had 49.67% accuracy. For the statistical test, the one-sample t test was applied to evaluate the accuracies based on its chance levels (Table 6).

**Table 1:** Feature-wise two-class classification of positive and negative displays averaged over 20 subjects (%) (MP/N: Menswear pos./neg., WP/N: Womenswear pos./neg., UP/N: Underwear pos./neg., SP/N: Sportswear pos./neg.).

Feature Combination	MP vs. MN	WP vs. WN	UP vs. UN	SP vs. SN
Mean-Peak	53.06	**64.95**	53.15	54.30
Mean-Variance	52.43	50.53	55.30	44.40
Mean-Skewness	55.73	53.67	60.86	51.89
Mean-Kurtosis	53.21	53.59	53.21	46.77
Mean-*t* value	50.18	50.44	54.20	45.28
Mean-Slope	54.14	54.45	56.41	56.35
Peak-Variance	44.58	49.01	36.67	35.68
Peak-Skewness	57.29	53.39	50.76	46.76
Peak-Kurtosis	41.00	47.44	38.76	43.13
Peak-*t* value	39.77	47.80	45.58	42.66
Peak-Slope	63.07	63.05	49.66	60.52
Variance-Skewness	51.57	50.28	54.81	47.81
Variance-Kurtosis	42.31	45.19	41.79	43.57
Variance-*t* value	44.44	47.81	45.76	46.35
Variance-Slope	**64.15**	60.91	58.65	**66.27**
Skewness-Kurtosis	53.21	58.68	60.60	60.18
Skewness-*t* value	49.74	49.07	**69.12**	47.13
Skewness-Slope	62.88	64.16	65.77	58.54
Kurtosis-*t* value	47.67	50.95	48.70	44.29
Kurtosis-Slope	62.44	60.99	46.22	61.71
*t* value- Slope	55.61	53.51	54.02	49.46

**Table 2:** Subject-wise two-class classification of positive and negative displays based on best feature combination (%).

No.	MP vs. MN	WP vs. WN	UP vs. UN	SP vs. SN
1	75.00	54.03	66.67	83.33
2	66.67	60.00	83.33	66.67
3	66.67	50.00	83.33	75.00
4	(42.86)	60.00	66.67	55.00
5	50.00	80.00	57.14	66.67
6	66.67	(40.00)	60.00	83.33
7	50.00	50.00	66.67	66.67
8	50.00	60.00	80.00	66.67
9	57.14	50.00	66.67	60.00
10	60.00	75.00	83.33	60.00
11	83.33	66.67	50.00	57.14
12	66.67	83.33	75.00	75.00
13	75.00	83.33	60.00	83.33
14	60.00	50.00	66.67	57.14
15	83.33	83.33	57.14	50.00
16	66.67	80.00	50.00	(42.74)
17	71.43	66.67	66.67	50.00
18	50.00	66.67	75.00	75.00
19	75.00	75.00	80.00	66.67
20	66.67	75.00	66.67	60.00
Avg. ± Std.	64.15 ± 11.51	65.45 ± 13.54	**67.04** ± 10.55	65.54 ± 11.62

**Table 3:** Feature-wise four-class classification of positive and negative displays averaged over 20 subjects (%).

Feature Combination	Positive Display	Negative Display
Mean-Peak	30.59	44.53
Mean-Variance	37.54	44.71
Mean-Skewness	49.63	40.53
Mean-Kurtosis	45.13	36.07
Mean-*t* value	37.10	44.08
Mean-Slope	31.53	41.46
Peak-Variance	30.58	28.66
Peak-Skewness	32.05	39.65
Peak-Kurtosis	(21.72)	39.60
Peak-*t* value	32.30	47.47
Peak-Slope	47.07	40.61
Variance-Skewness	44.56	40.70
Variance-Kurtosis	(22.85)	29.70
Variance-*t* value	33.34	49.19
Variance-Slope	50.62	40.03
Skewness-Kurtosis	48.54	35.79
Skewness-*t* value	35.56	40.36
Skewness-Slope	50.41	38.46
Kurtosis-*t* value	36.36	**51.10**
Kurtosis -Slope	39.26	39.73
*t* value–Slope	**50.70**	39.49

**Table 4:** Subject-wise four-class classification of positive vs. negative displays based on best feature combination (%).

No.	Positive Display	Negative Display
1	51.90	48.33
2	31.67	44.67
3	51.00	51.24
4	60.76	44.67
5	42.38	31.33
6	38.52	54.67
7	60.00	55.00
8	50.00	51.00
9	54.00	62.38
10	**76.67**	54.67
11	40.00	**66.95**
12	40.76	63.33
13	65.95	54.67
14	64.57	50.00
15	48.33	48.14
16	42.00	44.33
17	46.10	42.74
18	44.38	51.00
19	45.57	59.55
20	59.00	42.67
Avg. ± Std.	50.68 ± 11.08	51.07 ± 8.37

**Table 5:** Gender-specific classification accuracies (%).

Class	Category	Male	Female
Two class	MP vs. MN	58.50 ± 10.21	69.81 ±10.22
	WP vs. WN	57.90 ± 12.10	**73.00** ±10.68
	UP vs. UN	**71.38** ± 10.13	64.71 ±10.38
	SP vs. SN	68.33 ± 9.56	61.70 ±13.01
Four class	Positive Display	**51.69** ± 12.67	49.67 ± 9.81
	Negative Display	49.80 ± 8.38	**52.34** ± 8.61

**Table 6:** Statistical testing for comparison of classification accuracy: ^a^One sample *t*-test: * and ** indicate *p* < 0.05 and *p* < 0.01, respectively.

Class	Category	Subject	Accuracy (mean ± SD)	*t*-value	*p*-value^a^
Two-class	MP vs.MN	All	64.15 ± 11.51	5.4957	0.00**
		Male	58.50 ± 10.21	2.6327	0.0272*
		Female	69.81 ± 10.22	6.1296	0.0002*
	WP vs.WN	All	65.45 ± 13.54	5.103	0.00**
		Male	57.90 ± 12.10	2.0646	0.069
		Female	73.00 ± 10.68	6.8101	0.00**
	UP vs.UN	All	67.04 ± 10.55	7.2232	0.00**
		Male	71.38 ± 10.13	6.6742	0.00**
		Female	64.71 ± 10.38	4.4814	0.0015*
	SP vs.SN	All	65.54 ± 11.62	5.9808	0.00**
		Male	68.33 ± 9.56	6.0632	0.0002*
		Female	61.70 ± 13.01	2.8439	0.0193*
Four-class	Positive	All	50.68 ± 11.08	10.365	0.00**
		Male	51.69 ± 12.67	6.6615	0.00**
		Female	49.67 ± 9.81	7.9524	0.00**
	Negative	All	51.07 ± 8.37	13.9293	0.00**
		Male	49.80 ± 8.38	9.3585	0.00**
		Female	52.34 ± 8.61	10.0414	0.00**

## Discussion

In the present study, the authors used fNIRS to monitor brain signals for visual merchandising displays because the fNIRS machine is portable, silent, noninvasiveness, and low cost and allows an experiment comparable to fMRI. The adopted fNIRS technique has high potential as a brain imaging modality. In examining the brain activation, the maps and classification results were discussed in relation to the types of stores and genders. In this study, positive and negative displays from four categories (menswear store, womenswear store, underwear store, and sportswear store) were investigated. Gender-based classification was typically distinguishable in the categories of womenswear and sportswear.

We found that the activated brain regions were asymmetrical across subjects (see Figs 5 and 6). This kind of asymmetry in functional responses in the prefrontal cortex was observed in other reports as well [5, 8, 49]. The results from Achterberg et al. [49] showed more activation in the right dorsal lateral PFC during negative social pictures. Our results are consistent with their report since the negative displays of three of four categories (menswear, womenswear, and sportswear) showed higher activation in the right PFC (see Fig 6A), which illustrates the asymmetry of brain responses. The activation channel number based on *t-*test analysis shows that the number of positive display channels is greater than that of negative displays in the menswear and sportswear cases. Some previous studies [50, 51] reported that activation in the PFC is positively correlated with positive and negative art picture stimulation. Vessel et al. [51] found greater activation in the PFC for esthetically pleasing images. The gender-based *t*-maps were different (Fig 6B and 6C). This conclusion is consistent with previous research [52]. There are more active channels in menswear and underwear displays in the case of male subjects. Very few active channels were found in the womenswear displays. However, in the case of female subjects, womenswear displays produced more activated channels. Underwear displays yielded the poorest activation (see Fig 6C). The active brain region was asymmetrical in gender-based maps as well.

We applied an LDA classifier to the two-class classification problem of positive and negative displays (in which 21 different feature combinations were examined) and to the four-class classification problem with four stores. In classifying positive and negative menswear stores (see Table 1), the best accuracy was obtained from the combination of variance and slope, which was 64.15%. In the case of womenswear, the best accuracy was achieved from the combination of mean and peak values. In the case of underwear, the combination of skewness and *t*-value resulted in the highest accuracy, and lastly, in the case of sportswear, the combination of variance and slope gave the highest accuracy. It is noted that no common feature combination covering all four categories was found. However, as seen in Table 2, all averaged accuracies were higher than chance level (50%). Some subjects (subs. 11 and 15) had very high accuracy, up to 83.33%. Additionally, some subjects’ data showed poor accuracy lower than the chance level, which are marked in parentheses.

In the case of four-class classification for the four different stores, the results are more meaningful because the accuracies are much higher than the chance level (25%). Furthermore, gender-based classification accuracies provide a direct indication of vulnerable categories: females are sensitive to womenswear displays, men are sensitive to underwear displays, and both males and females are less emotional with menswear and sportswear displays. Based upon classification accuracies and activation maps, it is concluded that gender-specific store displays should be pursued. Positive and negative displays for different products are conveyed differently to the customer by different emotions [52, 53]. Such brain responses are recognized differently, as seen in the maps. From this view, the overall intensity of a map may be related to the customer’s interest. Therefore, the brain response from the PFC related to emotion or pleasantness by viewing positive or negative store pictures can be scrutinized in the early stages of interior design. This study indicates that the examination of brain activation will help fashion marketing in the future.

Even though some conclusions like the above can be made, current research reveals that the relationship between VMD and brain signals is still vague. There are only a few papers reporting the effectiveness of brain-based approaches. This lack of research is not because VMDs do not influence consumers, but because the machines to measure brain signals do not provide enough data. Therefore, a new technique to investigate VMDs and brain activation should be developed for neuro-marketing applications.

There are some factors that influence classification accuracy. i) Signal strength variation is due to several factors, including skull scalp thicknesses and hair pigment. ii) In the present study, we measured the PFC as a brain region without hair, but a more emotion-related brain region needs to be investigated. iii) A subject’s concentration level is another important factor to consider. During the experiment, some subjects reported that they felt sleepy and fatigued during the experiment closer to the end. Therefore, minimizing such subject-wise variation is another issue to consider. iv) Additionally, the surrounding environments also should be considered, such as machine noise. v) In this work, the pictures used in the experiment were obtained from the Internet. However, pictures attained from a planned store will provide a direct evaluation of the planned store. Finally, vi) cognitive effects between fixed and moving displays would be interesting [54].

## Conclusions

In this study, we examined the possibility of using fNIRS as a brain-based tool to investigate the consumer’s response for fashion store displays. The hemodynamic responses from the prefrontal cortices of twenty subjects were quite distinctive when viewing positive and negative store images. This finding implies that consumer responses to tentative plans of new stores can be evaluated in advance using fNIRS. Because new interior design costs a lot of money, the proposed brain-based approach can reduce total expenditure and construction time. In this study, even though hemodynamic responses to general (uncoordinated) store displays were examined, the hemodynamic responses of coordinated displays for a planned item would provide more design-specific evaluation for prospective consumers. Overall, consumer behavior analysis related to VMDs deserves to be evaluated using fNIRS. Future research should include a design-specific evaluation of VMDs focusing on store layouts, mannequins, light, points of purchase displays, colors, atmospherics, exterior displays, and others. As fashion changes quickly, to follow the directions of consumer behaviors, it is expected that we will see a rapid growth in interdisciplinary research and subsequent development of VMDs utilizing a brain-based approach in fashion marketing.

## Supporting information

S1 File Dataset. **The dataset for the four stores with positive/negative display stimulation (averaged over 20 subjects).** To understand our work, fNIRS task-related data were provided as supporting information.
(XLSX)

S2 File The survey. (PDF)
