# Supplementary material for: An fNIRS-based investigation of visual merchandising displays for fashion stores
Source: PLoS One. 2018 Dec 11;13(12):e0208843. doi: 10.1371/journal.pone.0208843 (PMC6289445; doi:10.1371/journal.pone.0208843)
Supplement: S2 File — (PDF) [file pone.0208843.s002.pdf]

The survey for defined the category of fashion store display stimulus
有关时尚店铺展示刺激定义分类的调查

In this survey, we will provide you 80 fashion store display pictures. Please carefully read each picture, evaluate whether the store display attracts you to purchase, and make a decision either you like or dislike it. In evaluating the quality of the fashion store display, a 10-point scale was used (10 means the well displayed and 1 means the poorly displayed).

通过本次调查，我们将展示80张时尚店面图片。请仔细阅读每一张图片，评估店面展示是否吸引你去购买店内商品，然后对每张图片展示做出决定喜欢与否。调查采用10分值（1分为最为不好的展示，10分为最好的展示）选择方法来评估您对店铺选择。

Display #1. Please evaluate this store from 1 to 10.

1	2	3	4	5	6	7	8	9	10

Poorly displayed **Average** **Well displayed**

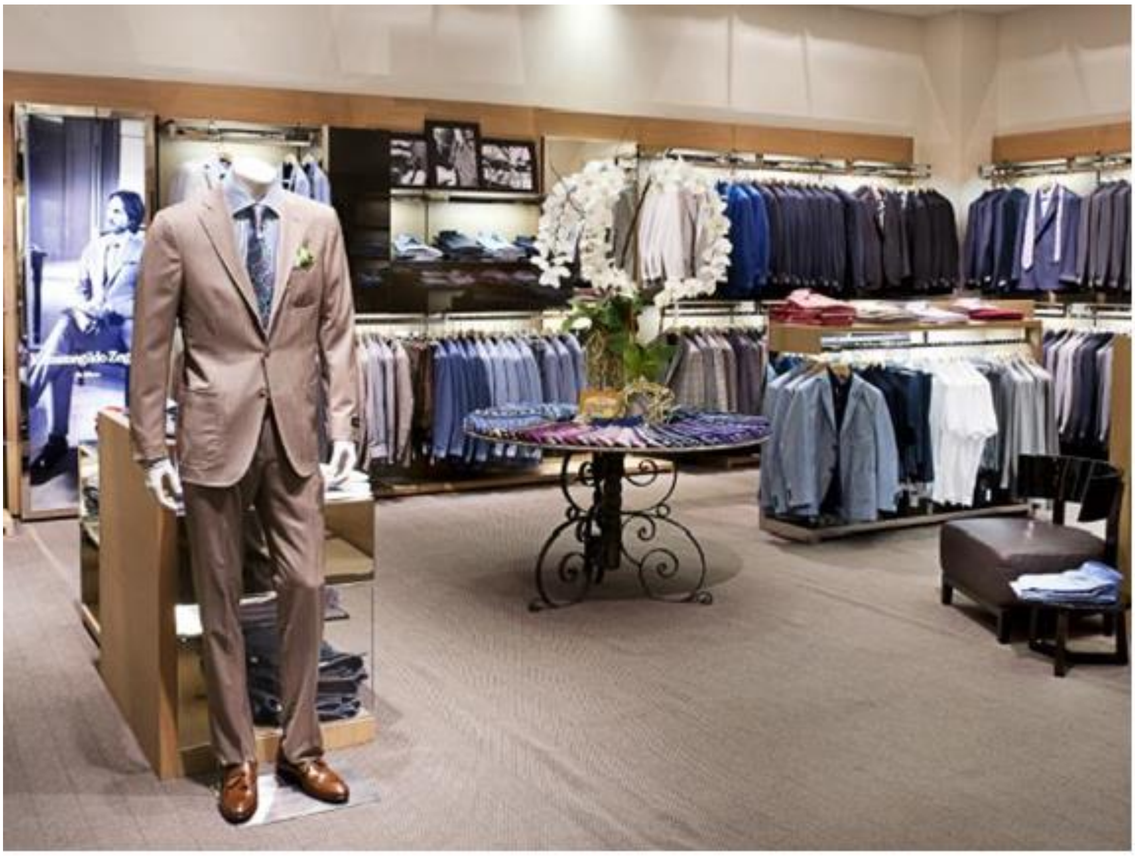

Display #2. Please evaluate this store from 1 to 10.

1	2	3	4	5	6	7	8	9	10

Poorly displayed **Average** **Well displayed**

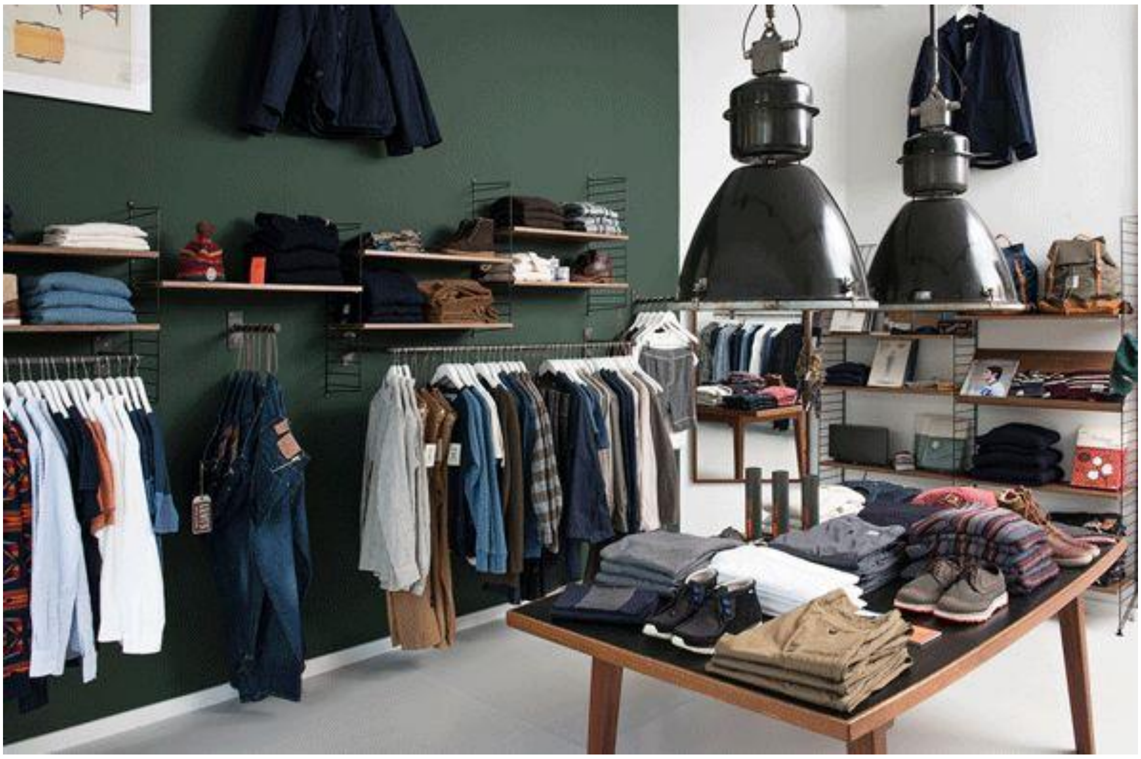

Display #3.   Please evaluate this store from 1 to 10.

1	2	3	4	5	6	7	8	9	10

Poorly displayed **Average** **Well displayed**

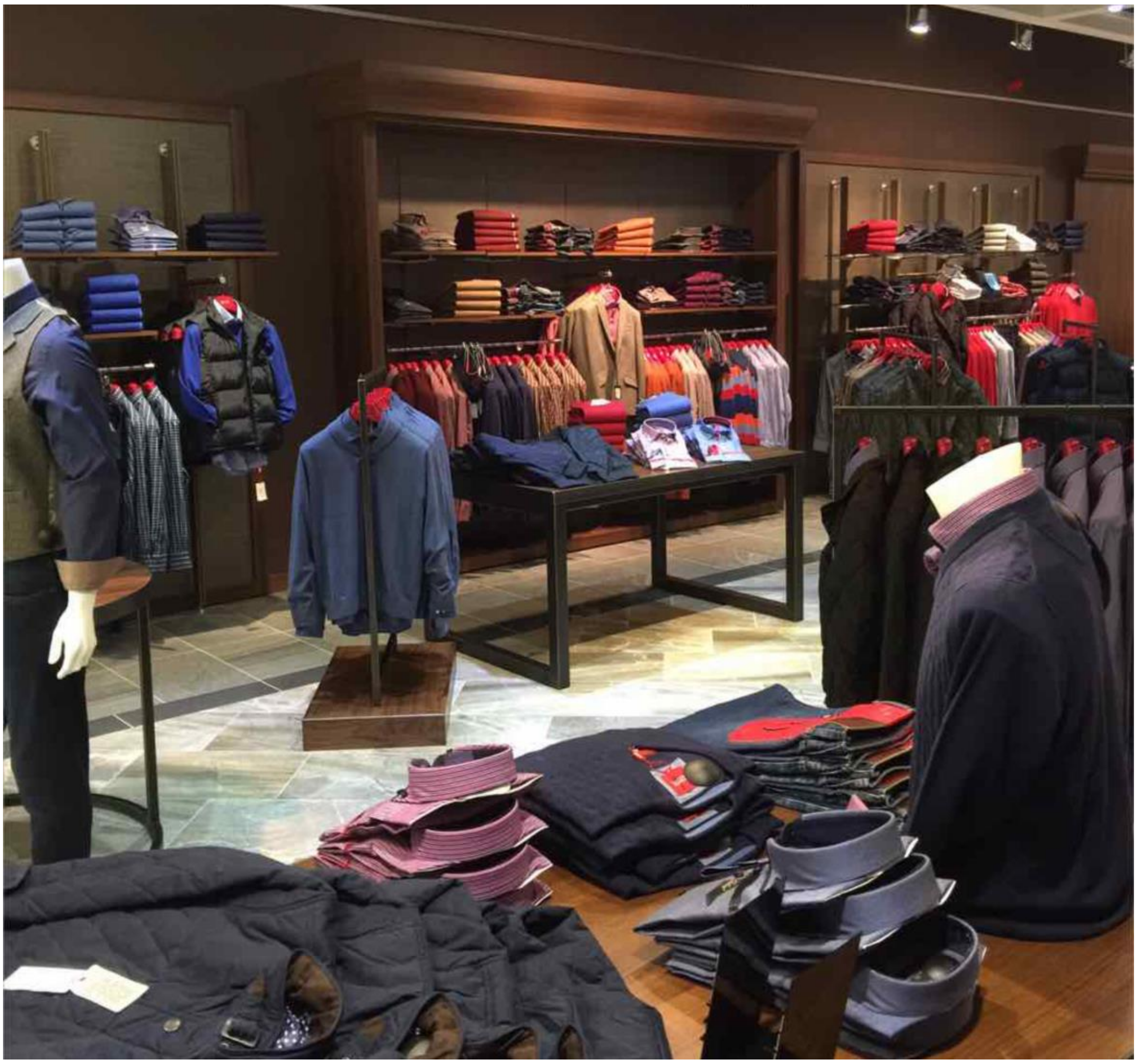

Display #4. Please evaluate this store from 1 to 10.

1	2	3	4	5	6	7	8	9	10

Poorly displayed **Average** **Well displayed**

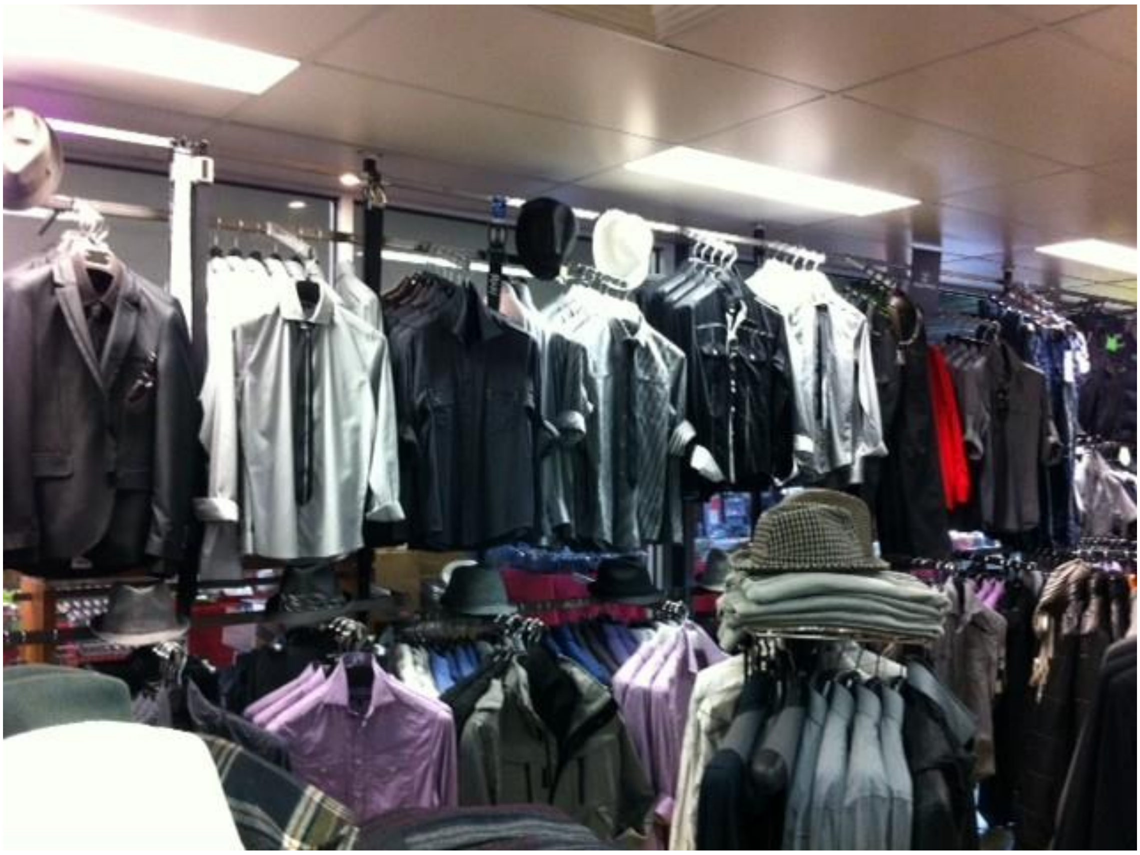

Display #5.   Please evaluate this store from 1 to 10.

1	2	3	4	5	6	7	8	9	10

Poorly displayed **Average** **Well displayed**

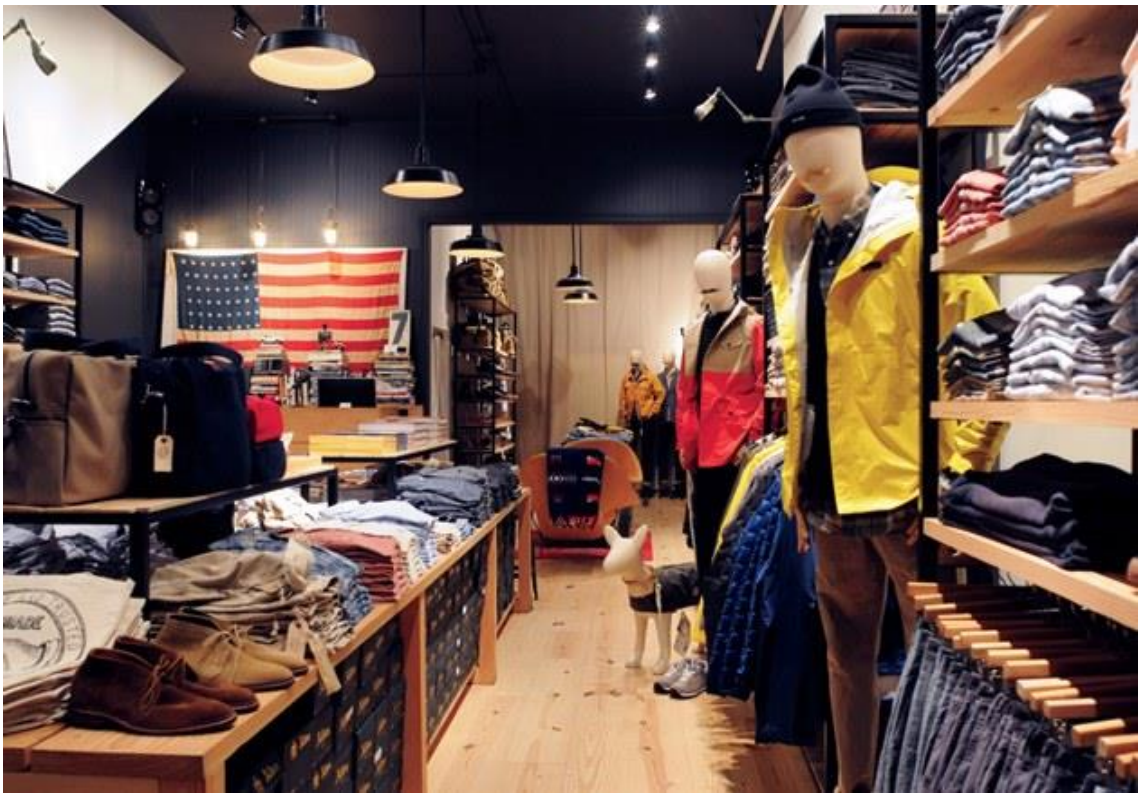

Display #6. Please evaluate this store from 1 to 10.

**1**	**2**	**3**	**4**	**5**	**6**	**7**	**8**	**9**	**10**

Poorly displayed **Average** **Well displayed**

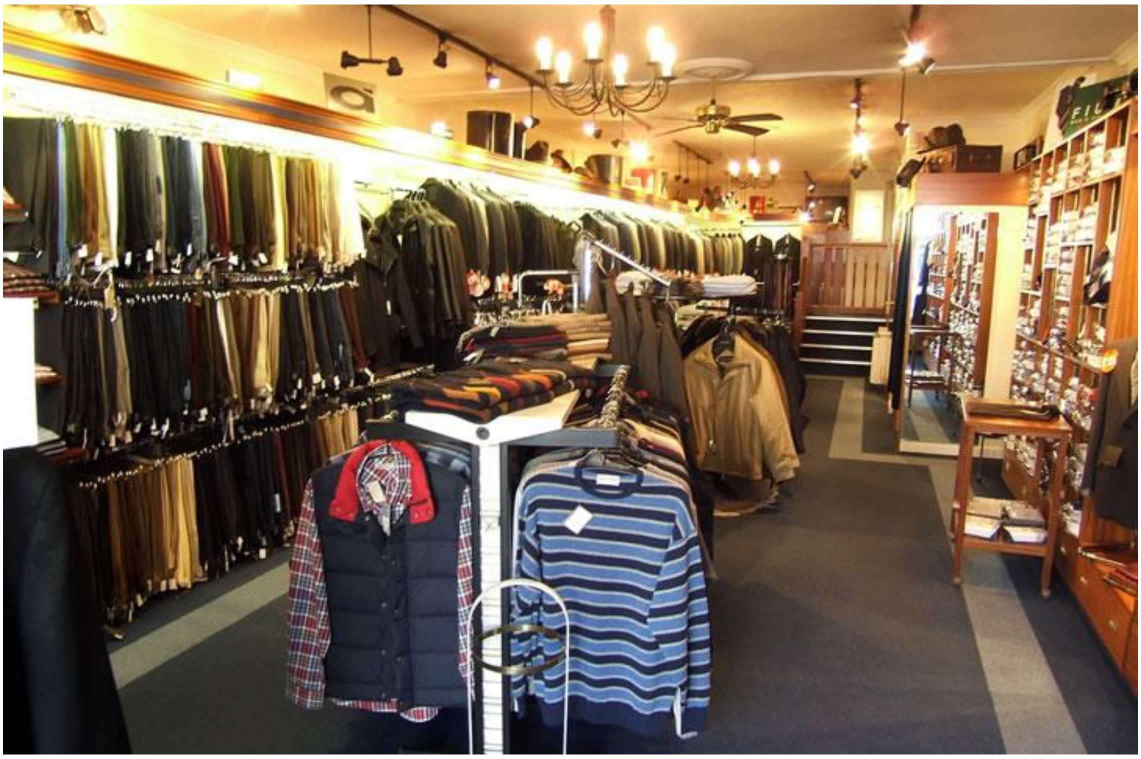

Display #7. Please evaluate this store from 1 to 10.

1	2	3	4	5	6	7	8	9	10

Poorly displayed **Average** **Well displayed**

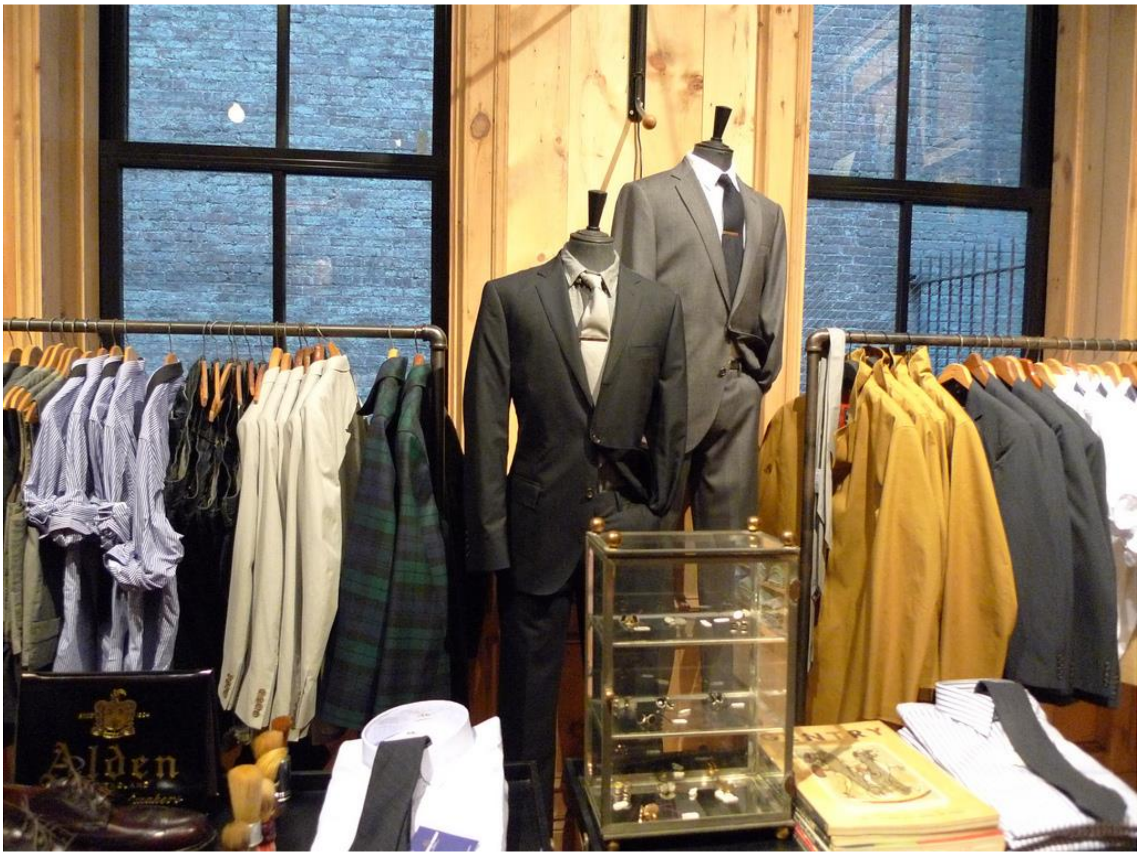

Display #8. Please evaluate this store from 1 to 10.

1	2	3	4	5	6	7	8	9	10

Poorly displayed **Average** **Well displayed**

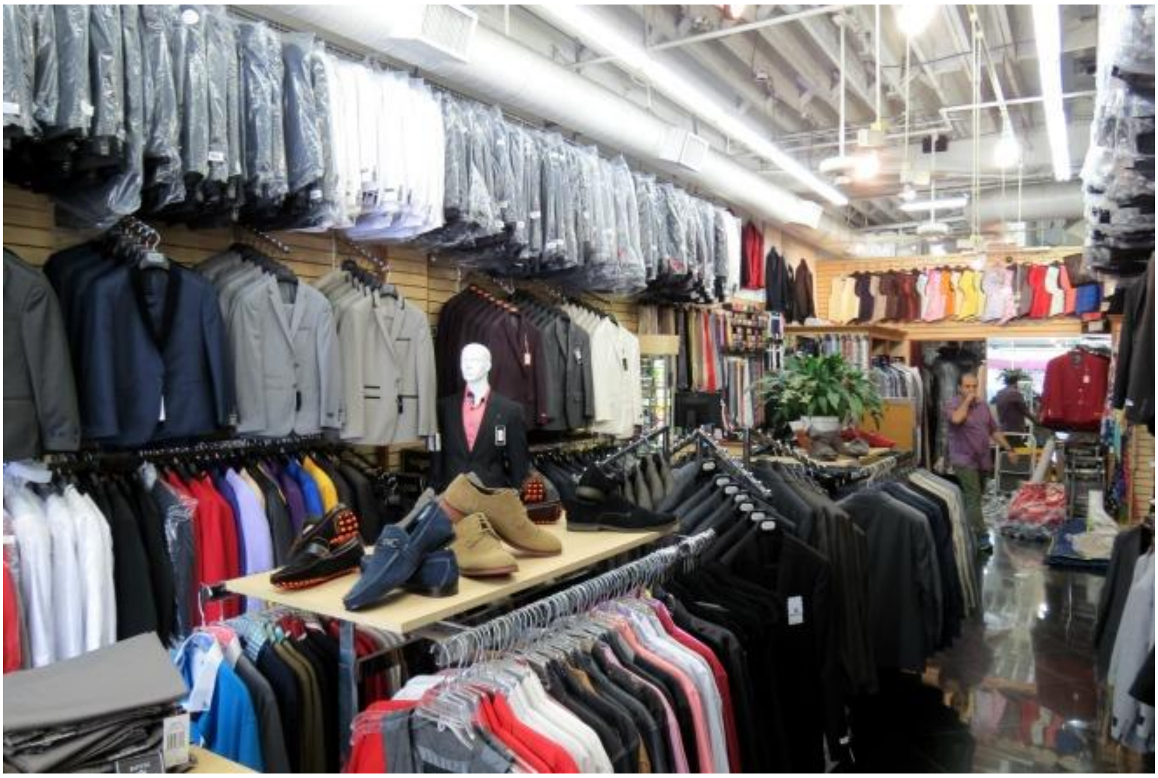

Display #9. Please evaluate this store from 1 to 10.

1	2	3	4	5	6	7	8	9	10

Poorly displayed **Average** **Well displayed**

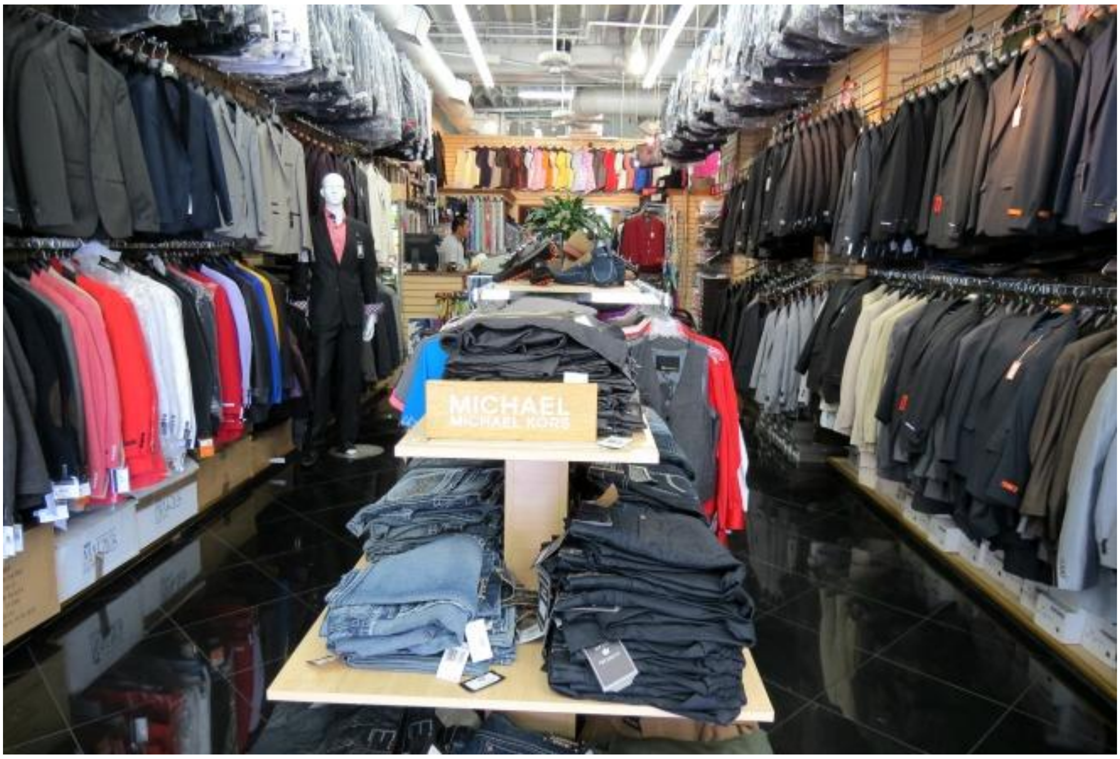

Display #10.   Please evaluate this store from 1 to 10.

1	2	3	4	5	6	7	8	9	10

Poorly displayed **Average** **Well displayed**

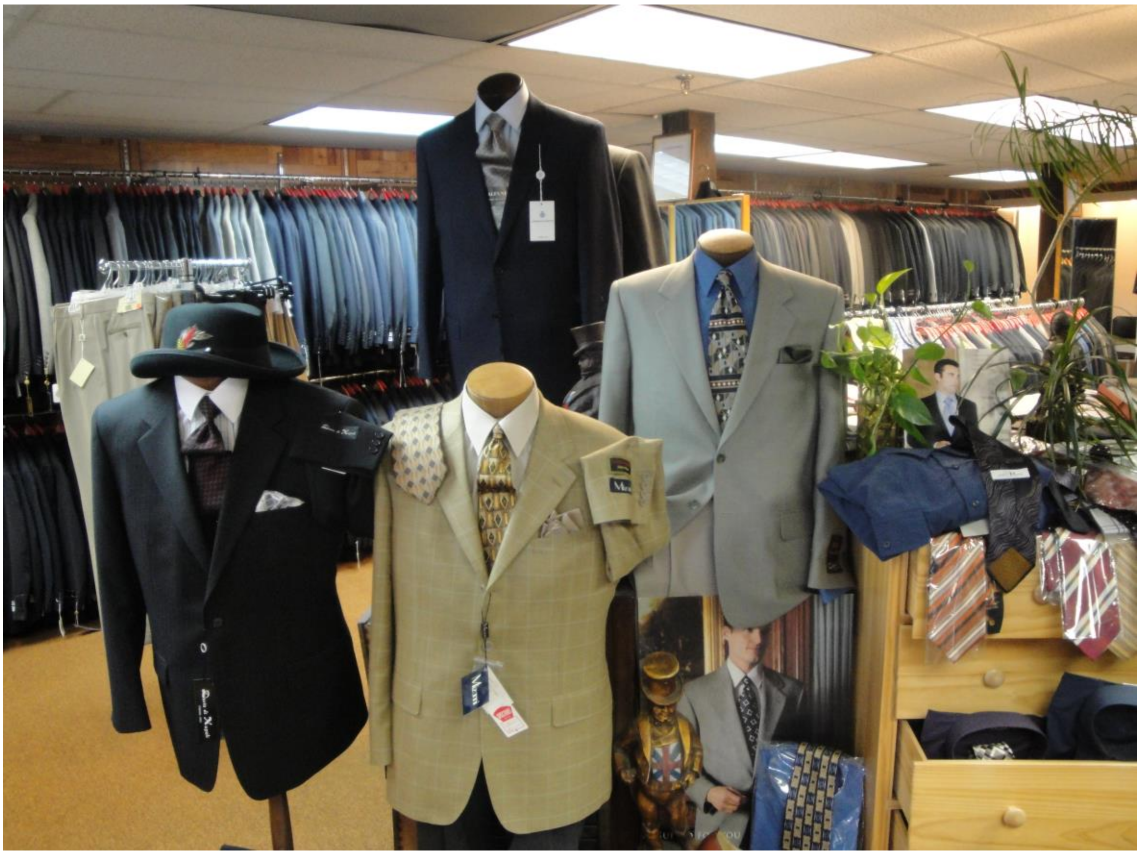

Display #11．Please evaluate this store from 1 to 10.

1	2	3	4	5	6	7	8	9	10

Poorly displayed **Average** **Well displayed**

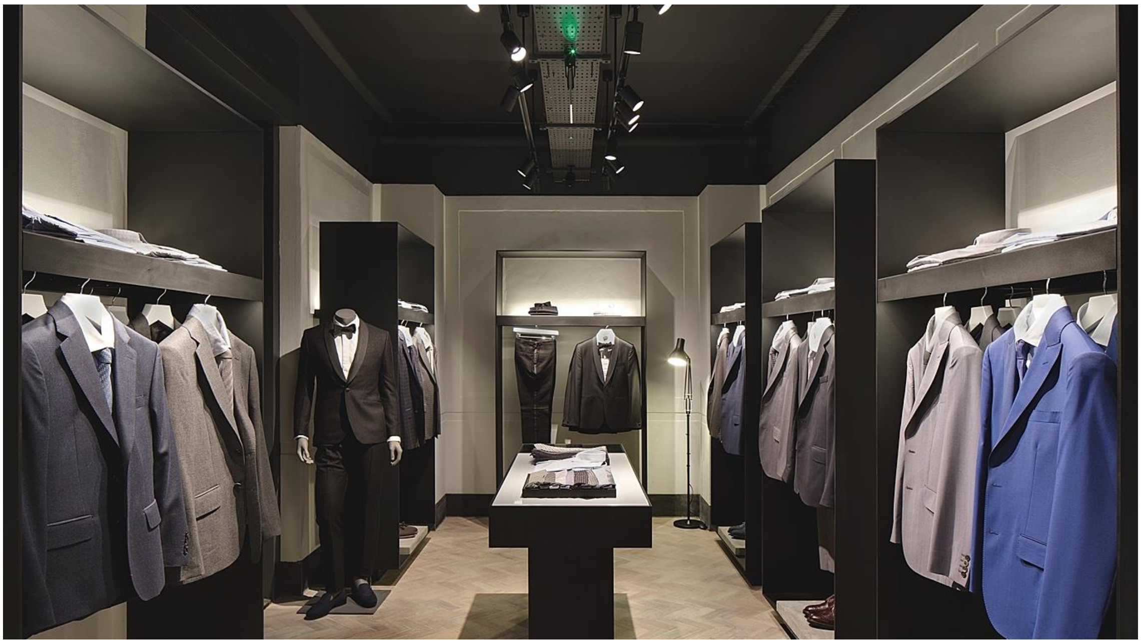

Display #12. Please evaluate this store from 1 to 10.

1	2	3	4	5	6	7	8	9	10

Poorly displayed **Average** **Well displayed**

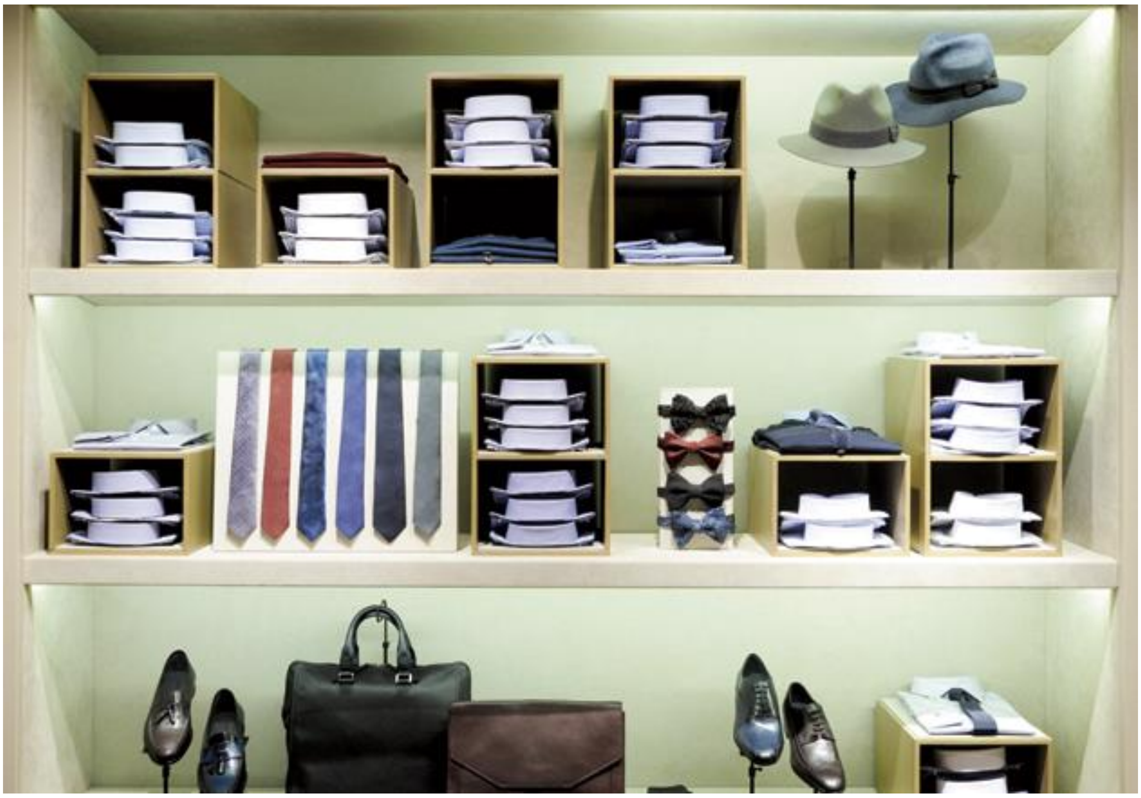

Display #13. Please evaluate this store from 1 to 10.

1	2	3	4	5	6	7	8	9	10

Poorly displayed **Average** **Well displayed**

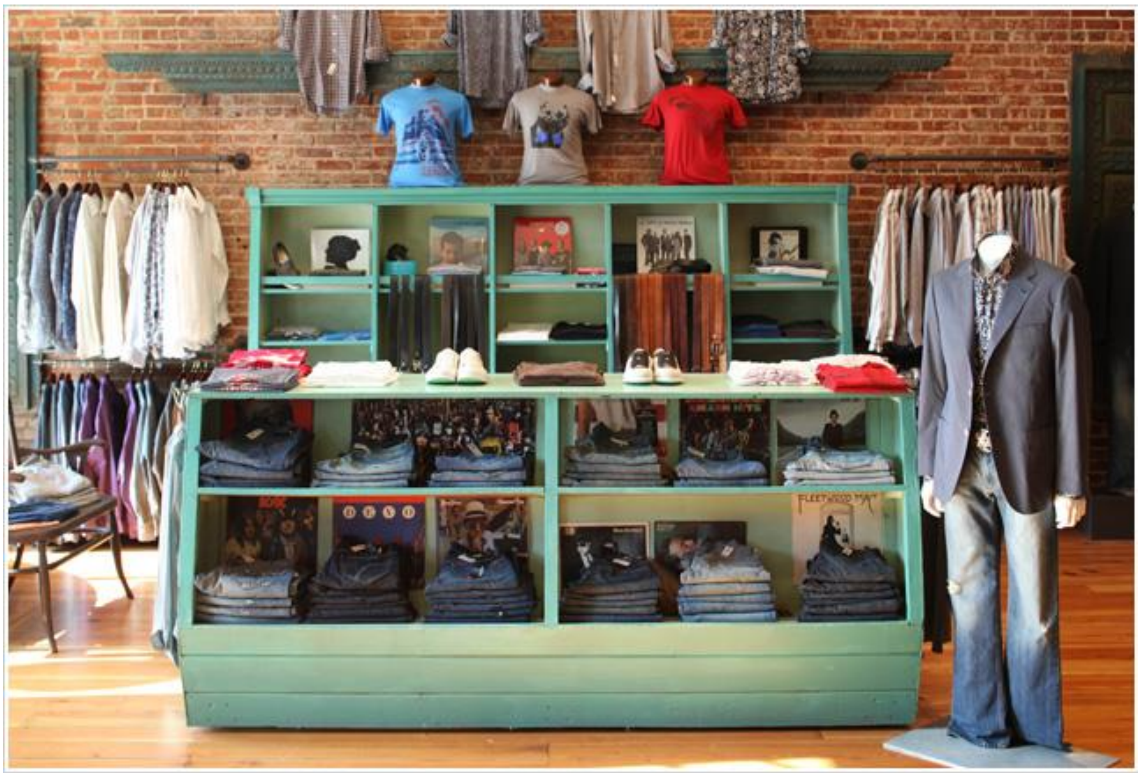

Display #14. Please evaluate this store from 1 to 10.

1	2	3	4	5	6	7	8	9	10

Poorly displayed **Average** **Well displayed**

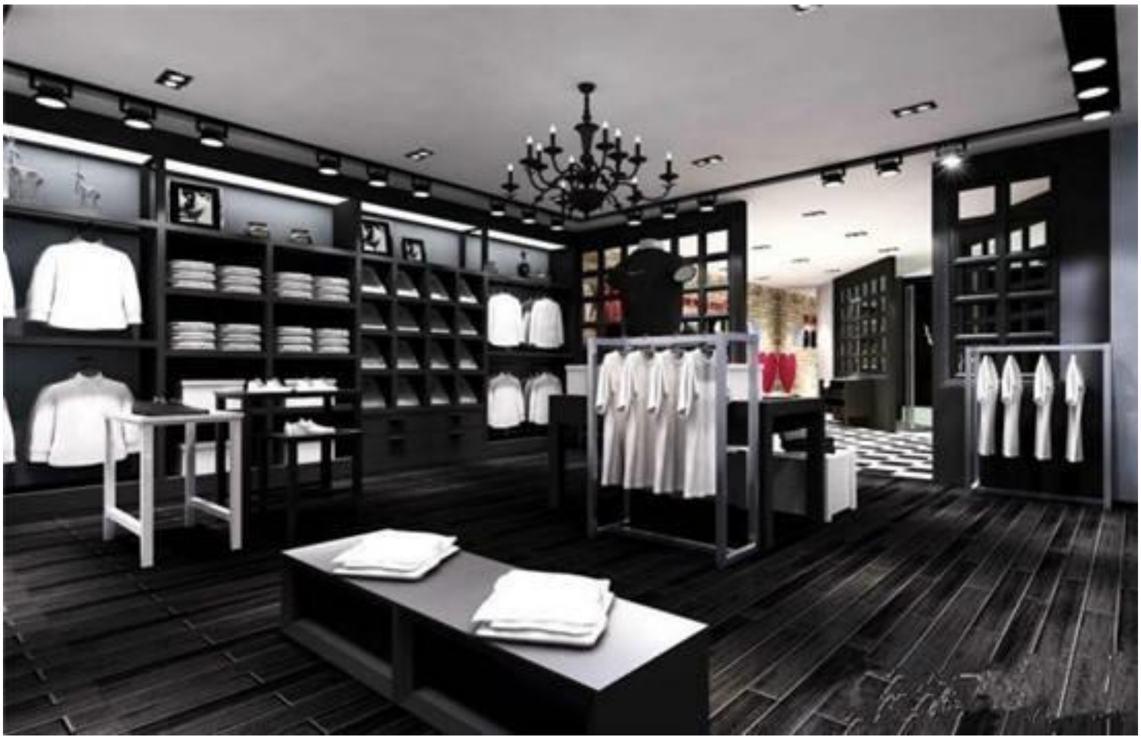

Display #15. Please evaluate this store from 1 to 10.

1	2	3	4	5	6	7	8	9	10

Poorly displayed **Average** **Well displayed**

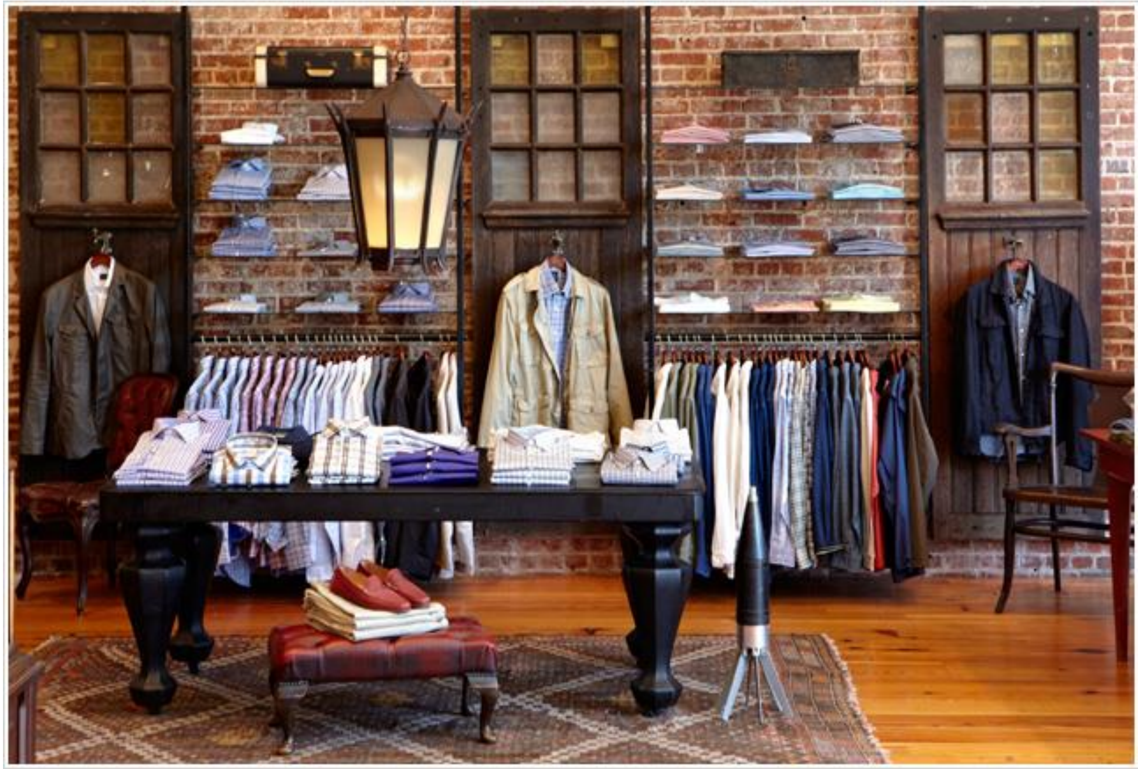

Display #16. Please evaluate this store from 1 to 10.

1	2	3	4	5	6	7	8	9	10

Poorly displayed **Average** **Well displayed**

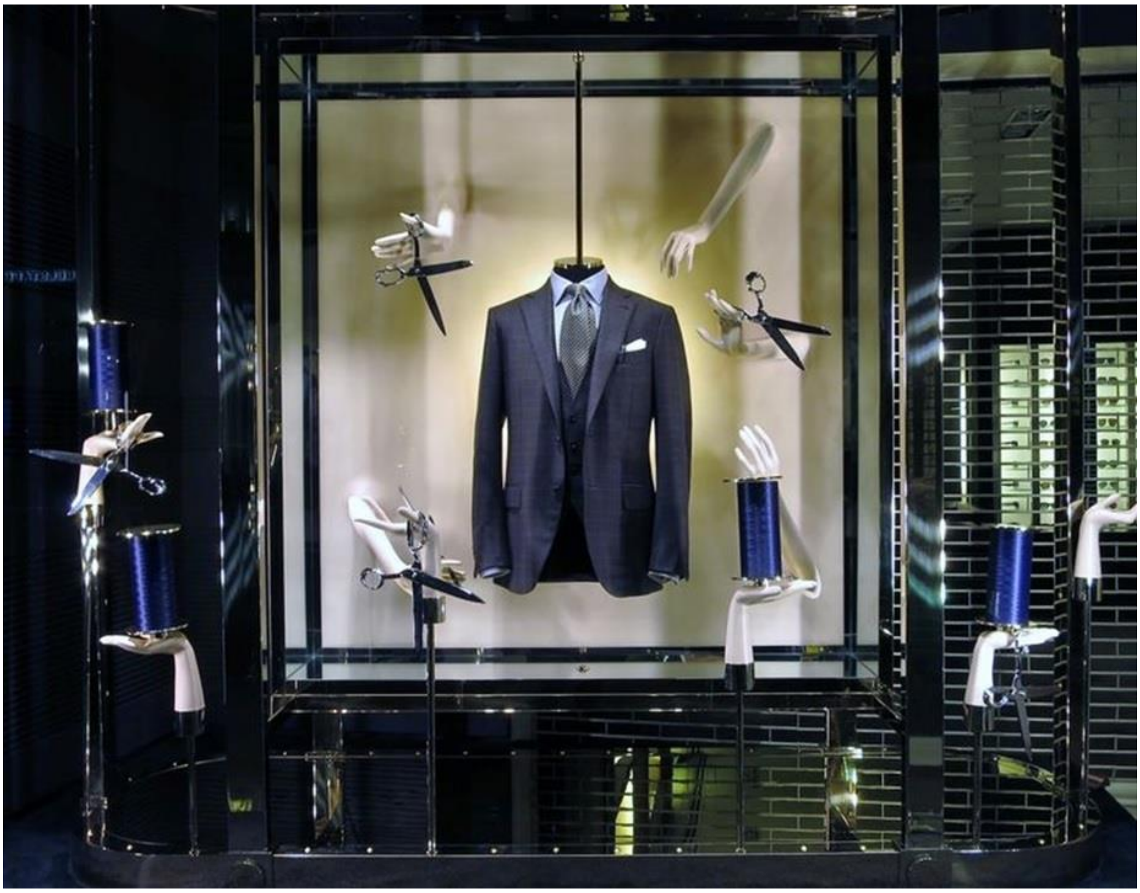

Display #17. Please evaluate this store from 1 to 10.

1	2	3	4	5	6	7	8	9	10

Poorly displayed **Average** **Well displayed**

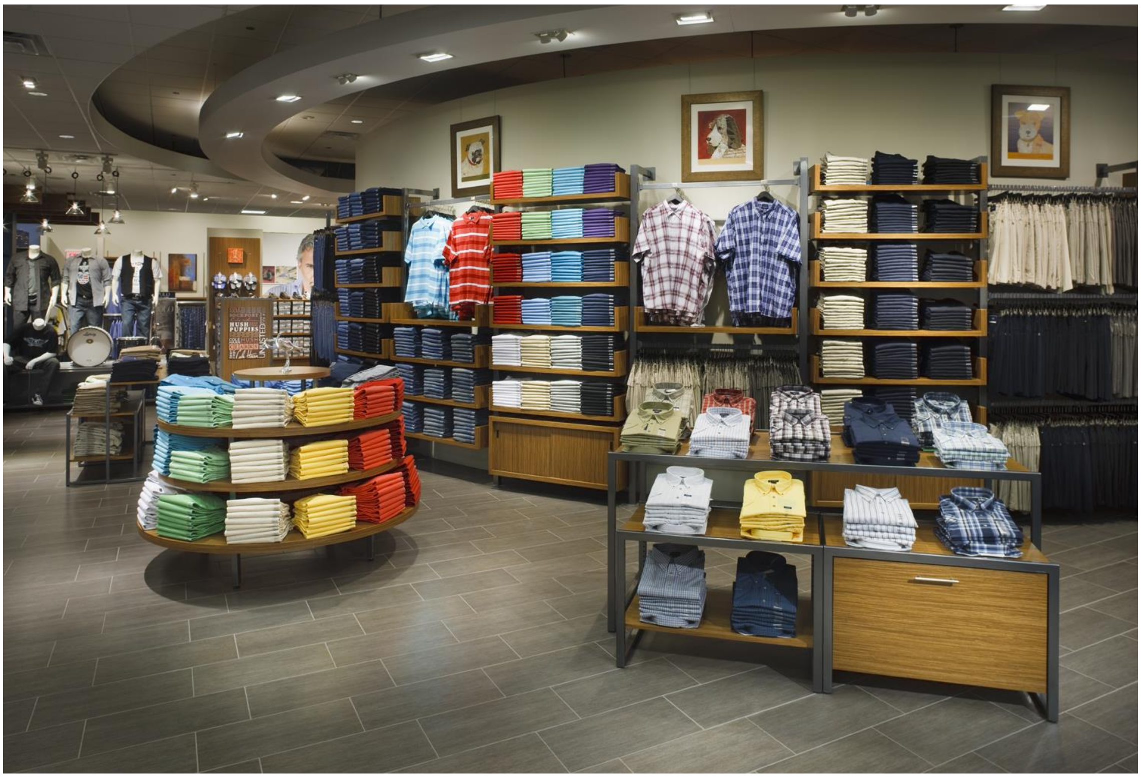

Display #18. Please evaluate this store from 1 to 10.

1	2	3	4	5	6	7	8	9	10

Poorly displayed **Average** **Well displayed**

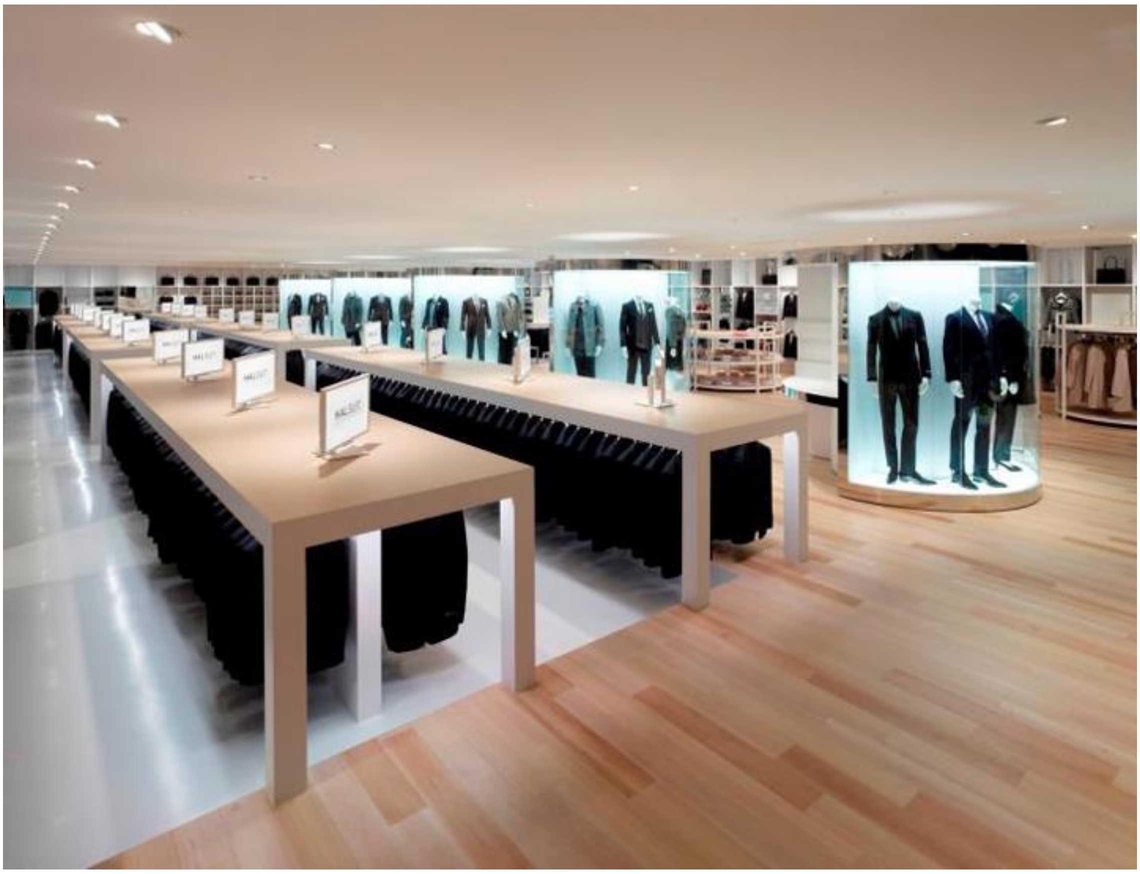

Display #19.   Please evaluate this store from 1 to 10.

1	2	3	4	5	6	7	8	9	10

Poorly displayed **Average** **Well displayed**

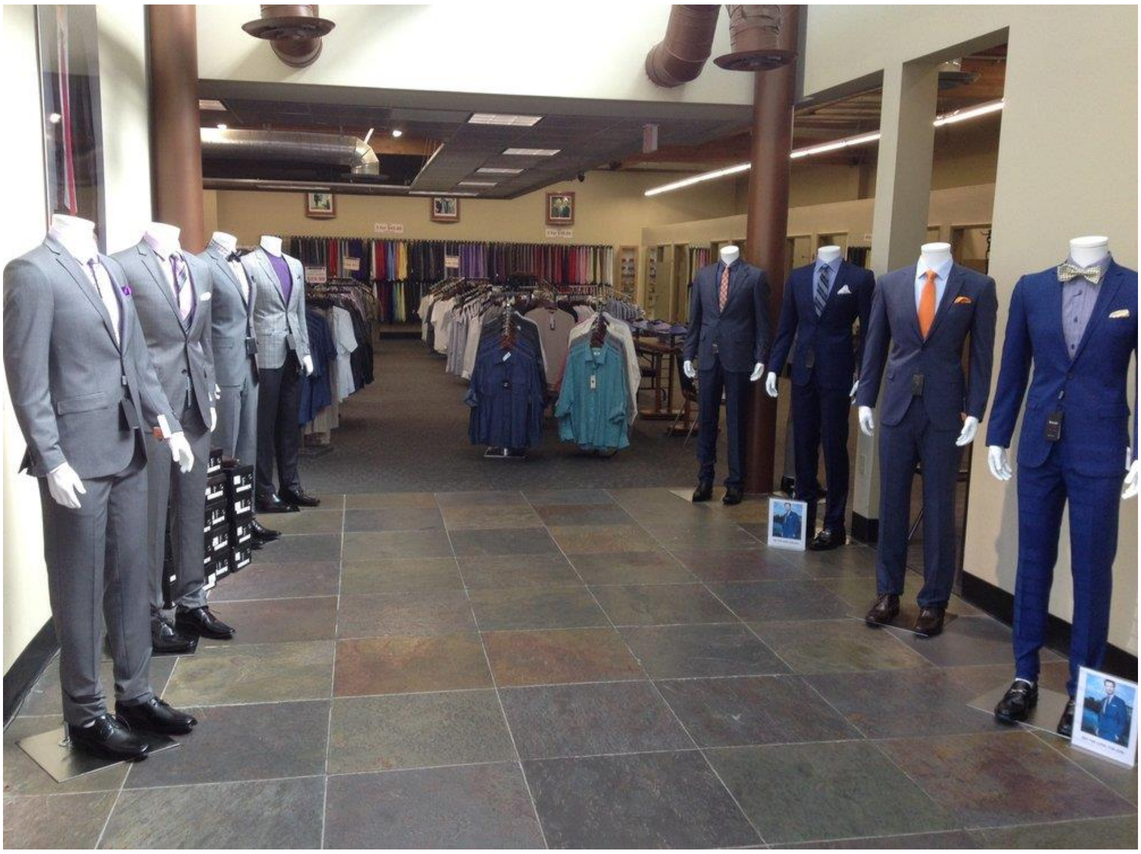

Display #20. Please evaluate this store from 1 to 10.

1	2	3	4	5	6	7	8	9	10

Poorly displayed **Average** **Well displayed**

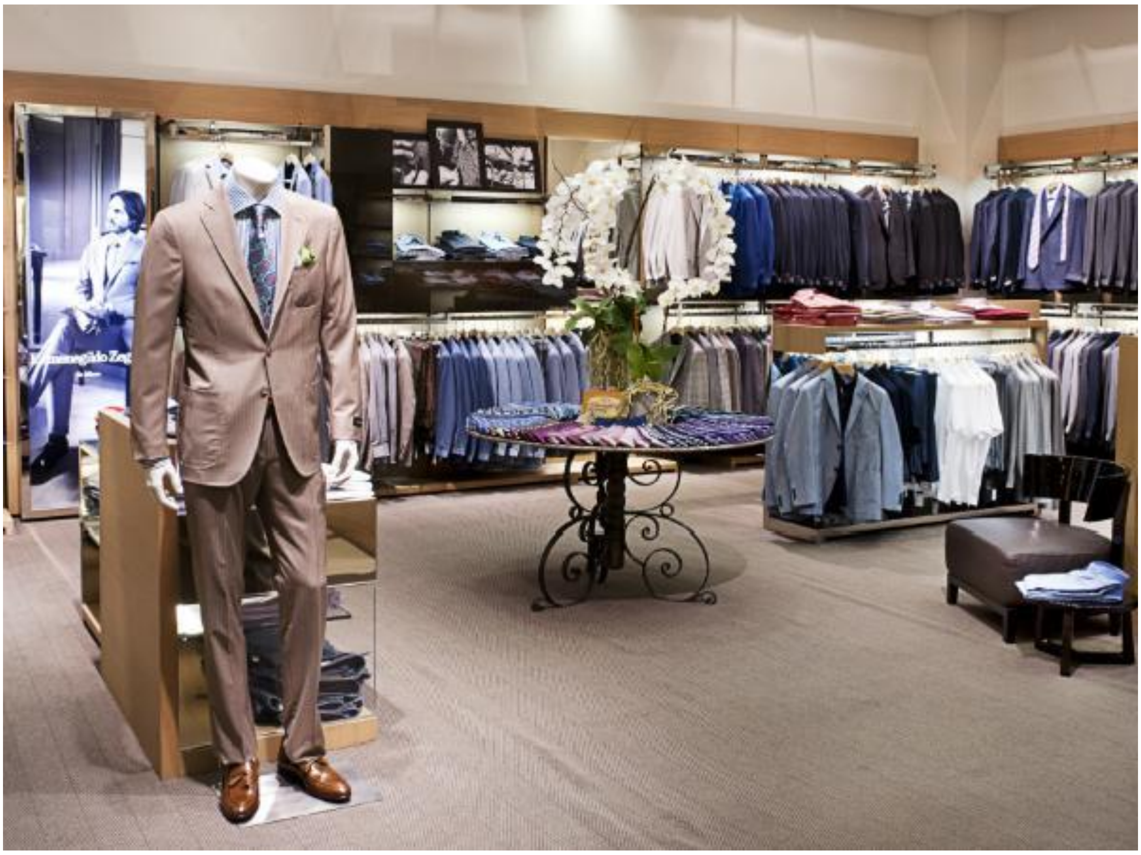

Display #21． Please evaluate this store from 1 to 10.

1	2	3	4	5	6	7	8	9	10

Poorly displayed **Average** **Well displayed**

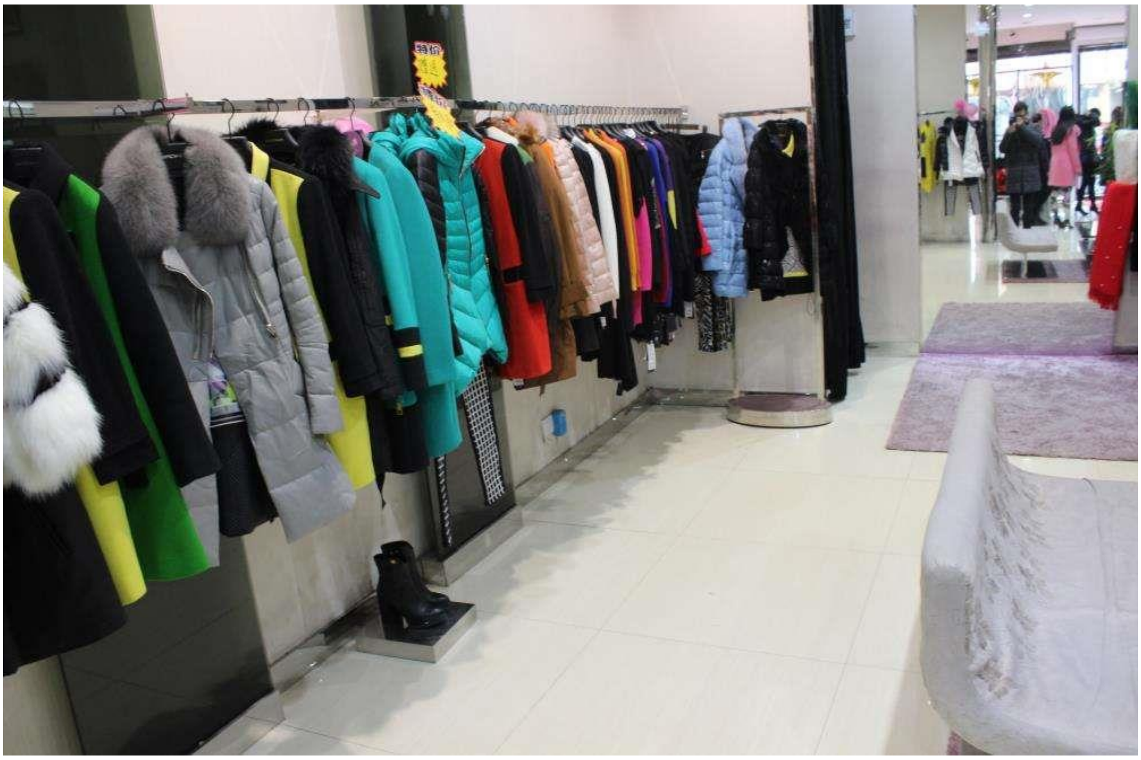

Display #22． Please evaluate this store from 1 to 10.

1	2	3	4	5	6	7	8	9	10

Poorly displayed **Average** **Well displayed**

喜欢

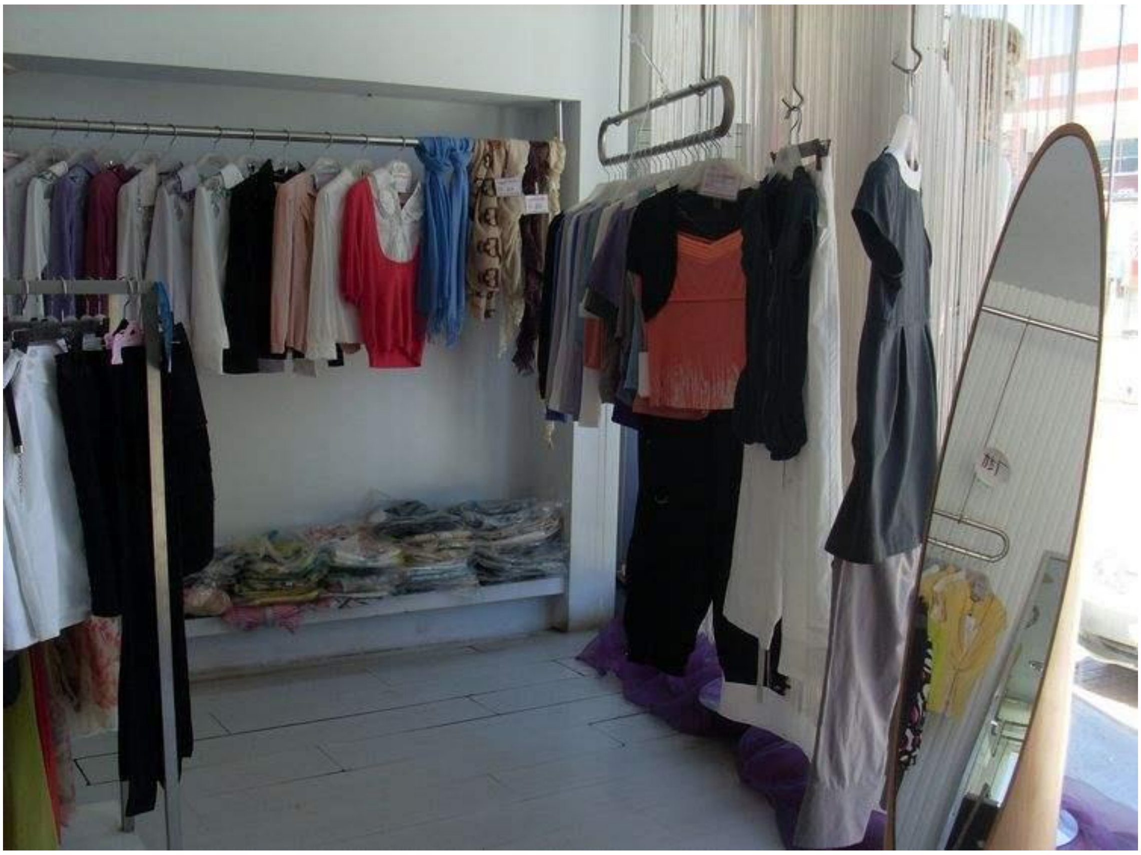

Display #23. Please evaluate this store from 1 to 10.

1	2	3	4	5	6	7	8	9	10

Poorly displayed **Average** **Well displayed**

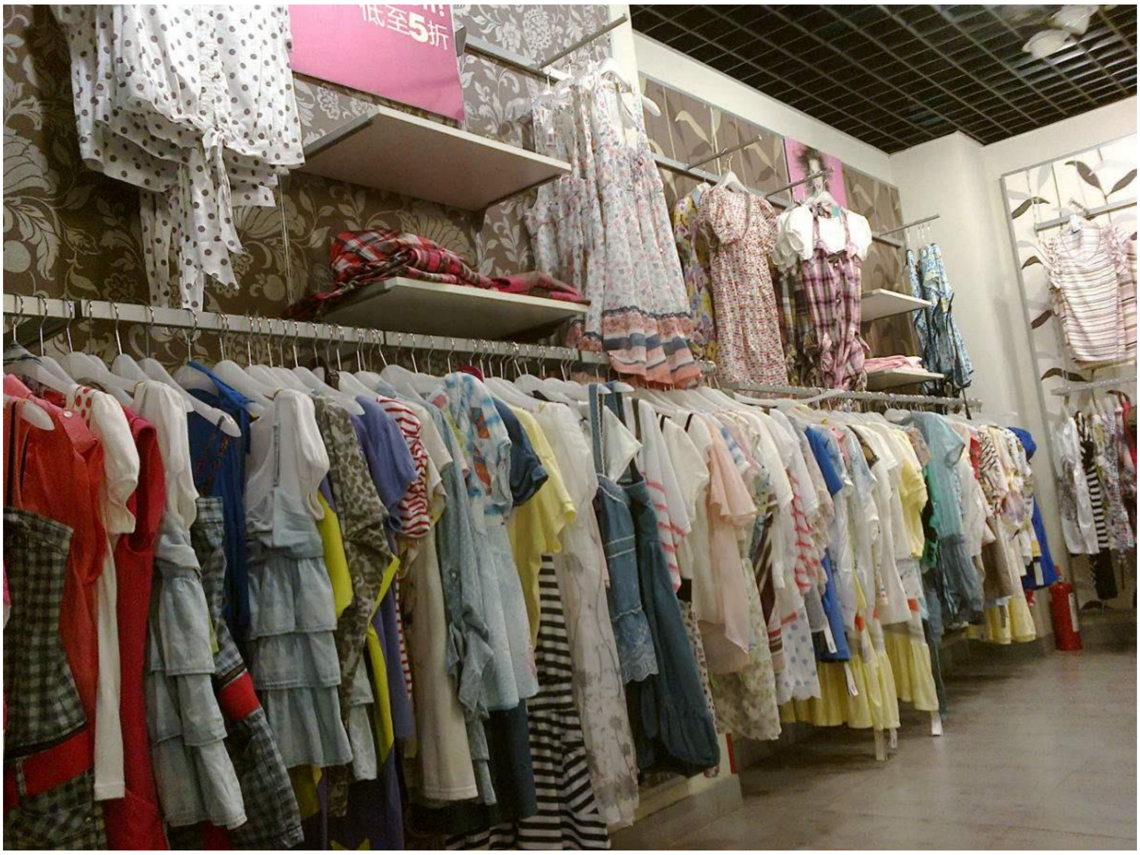

Display #24. Please evaluate this store from 1 to 10.

1	2	3	4	5	6	7	8	9	10

Poorly displayed **Average** **Well displayed**

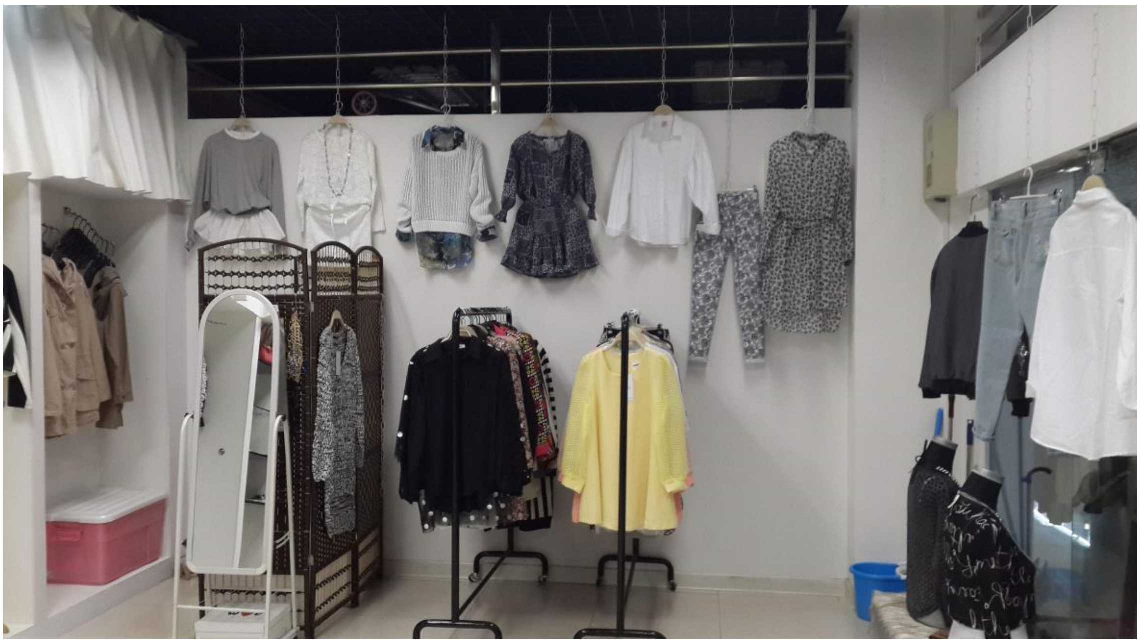

Display #25. Please evaluate this store from 1 to 10.

1	2	3	4	5	6	7	8	9	10

Poorly displayed **Average** **Well displayed**

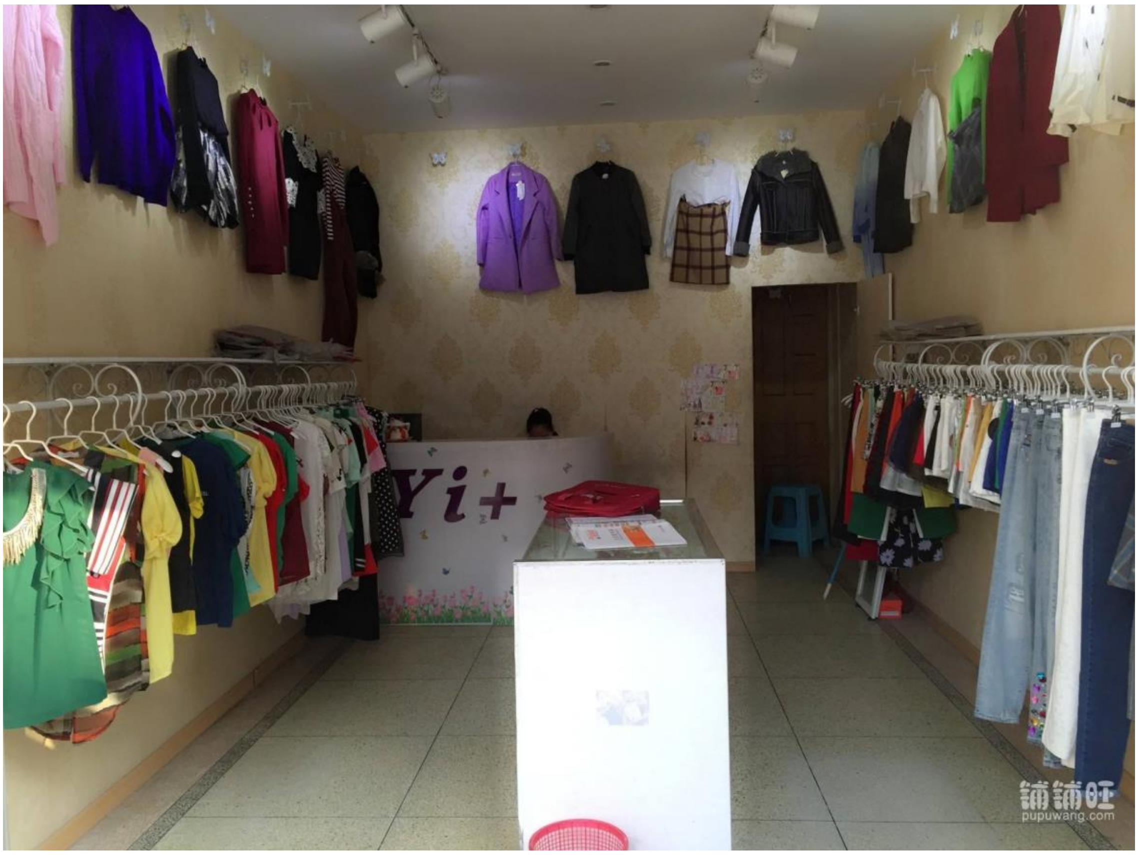

Display #26.   Please evaluate this store from 1 to 10.

**1**	**2**	**3**	**4**	**5**	**6**	**7**	**8**	**9**	**10**

Poorly displayed **Average** **Well displayed**

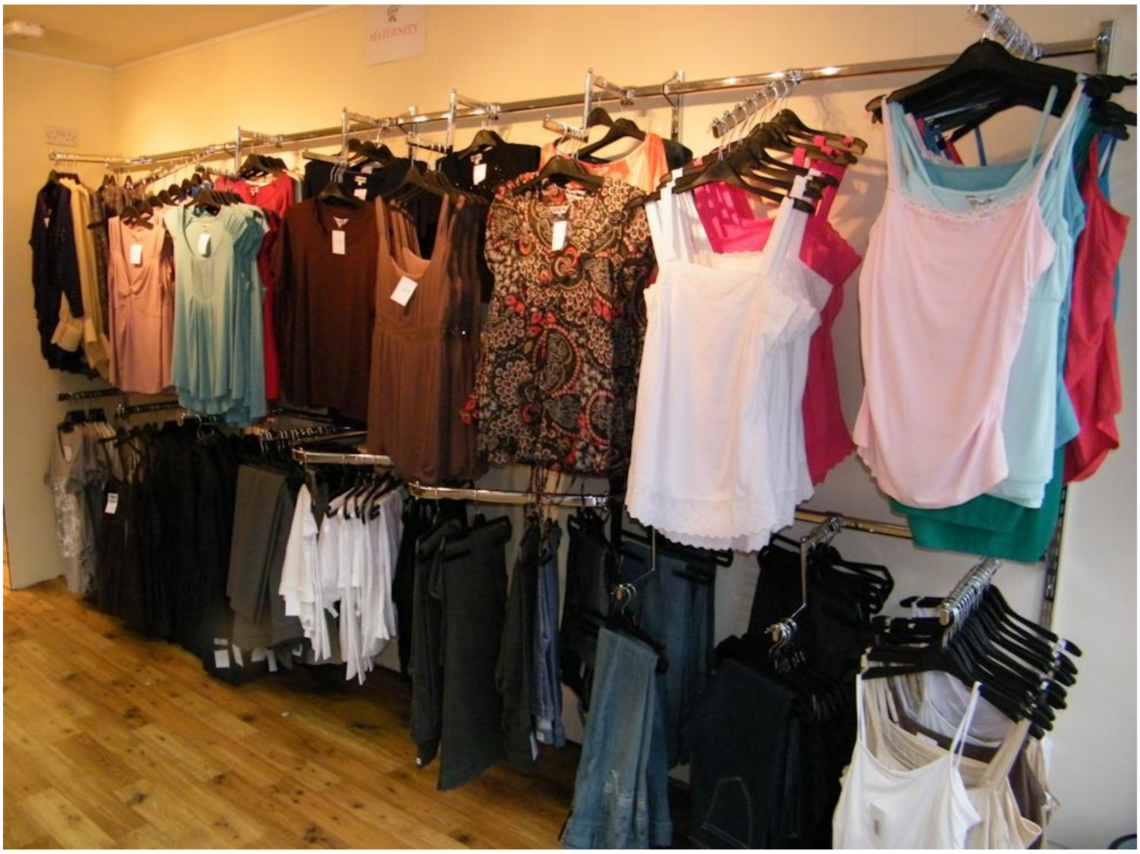

Display #27. Please evaluate this store from 1 to 10.

1	2	3	4	5	6	7	8	9	10

Poorly displayed **Average** **Well displayed**

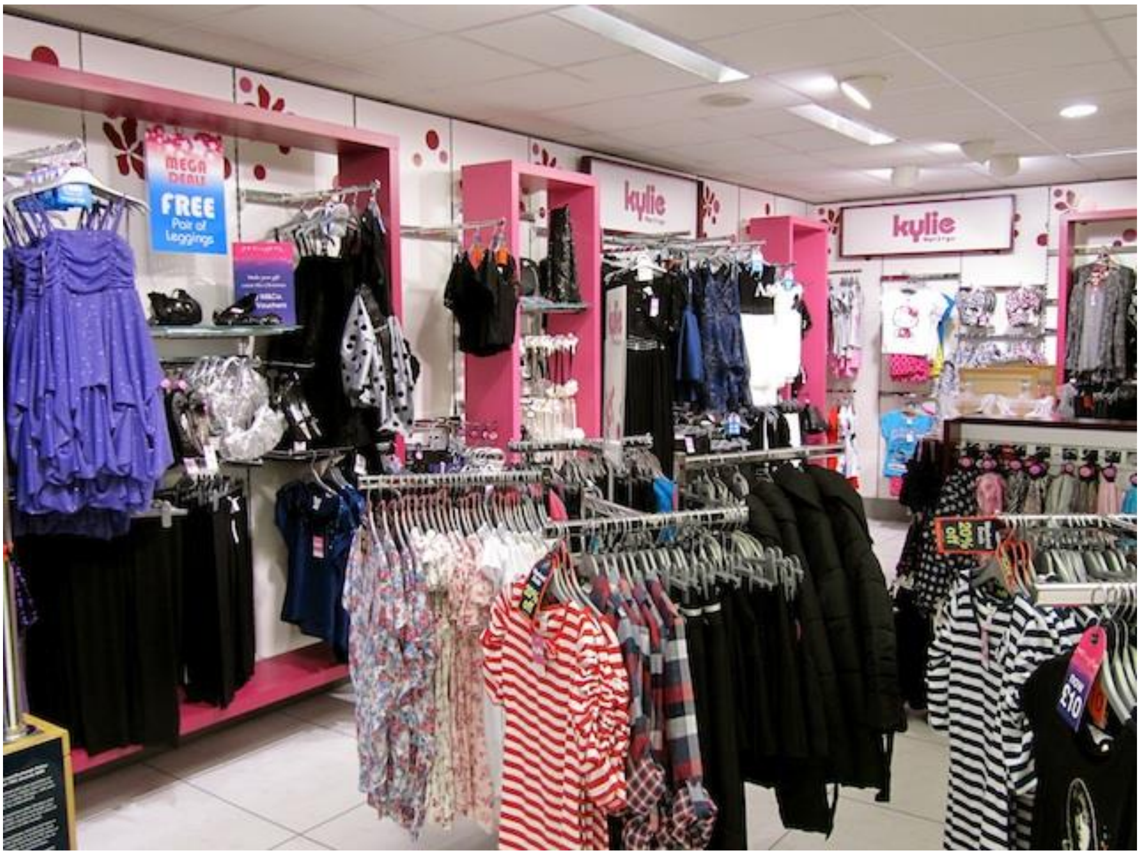

Display #28. Please evaluate this store from 1 to 10.

1	2	3	4	5	6	7	8	9	10

Poorly displayed **Average** **Well displayed**

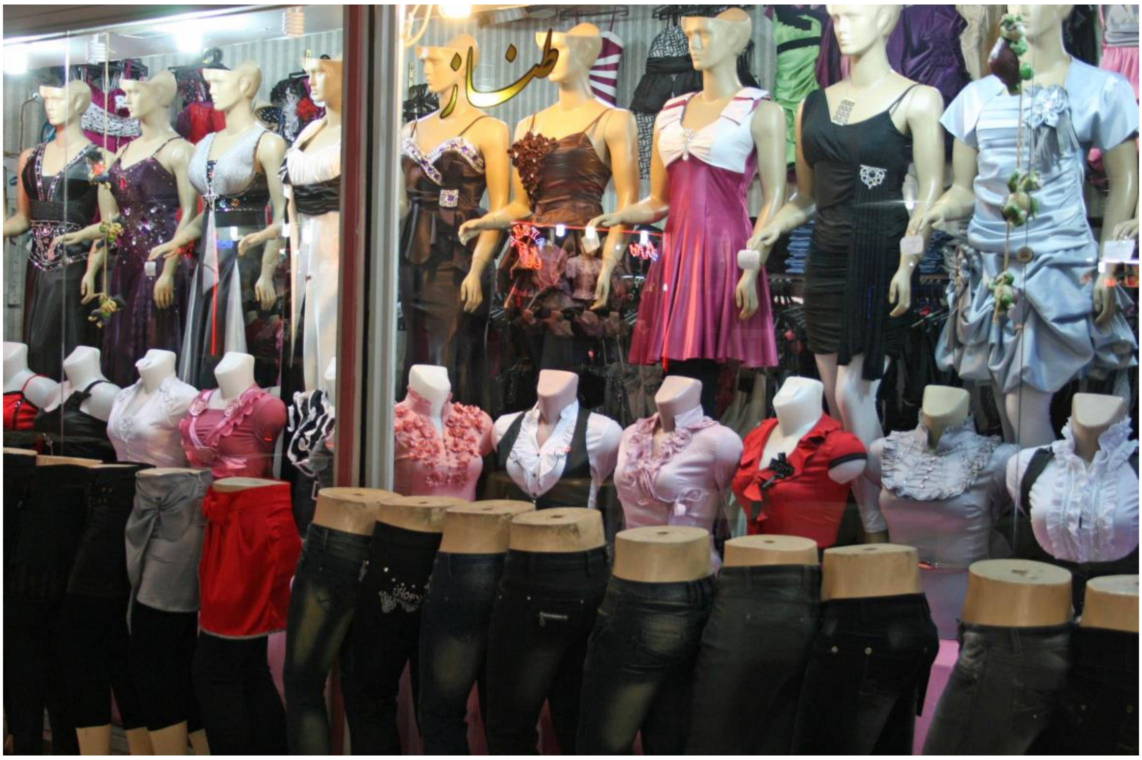

Display #29. Please evaluate this store from 1 to 10.

1	2	3	4	5	6	7	8	9	10

Poorly displayed **Average** **Well displayed**

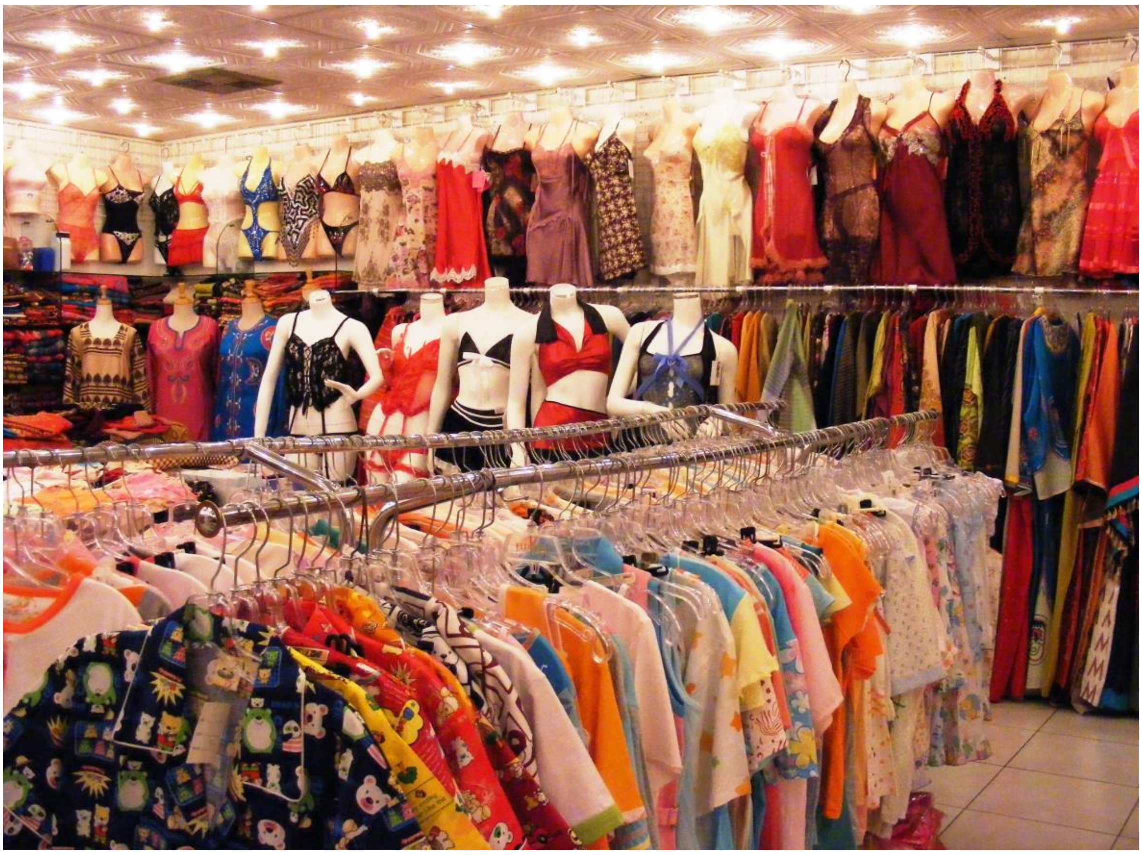

Display #30. Please evaluate this store from 1 to 10.

1	2	3	4	5	6	7	8	9	10

Poorly displayed **Average** **Well displayed**

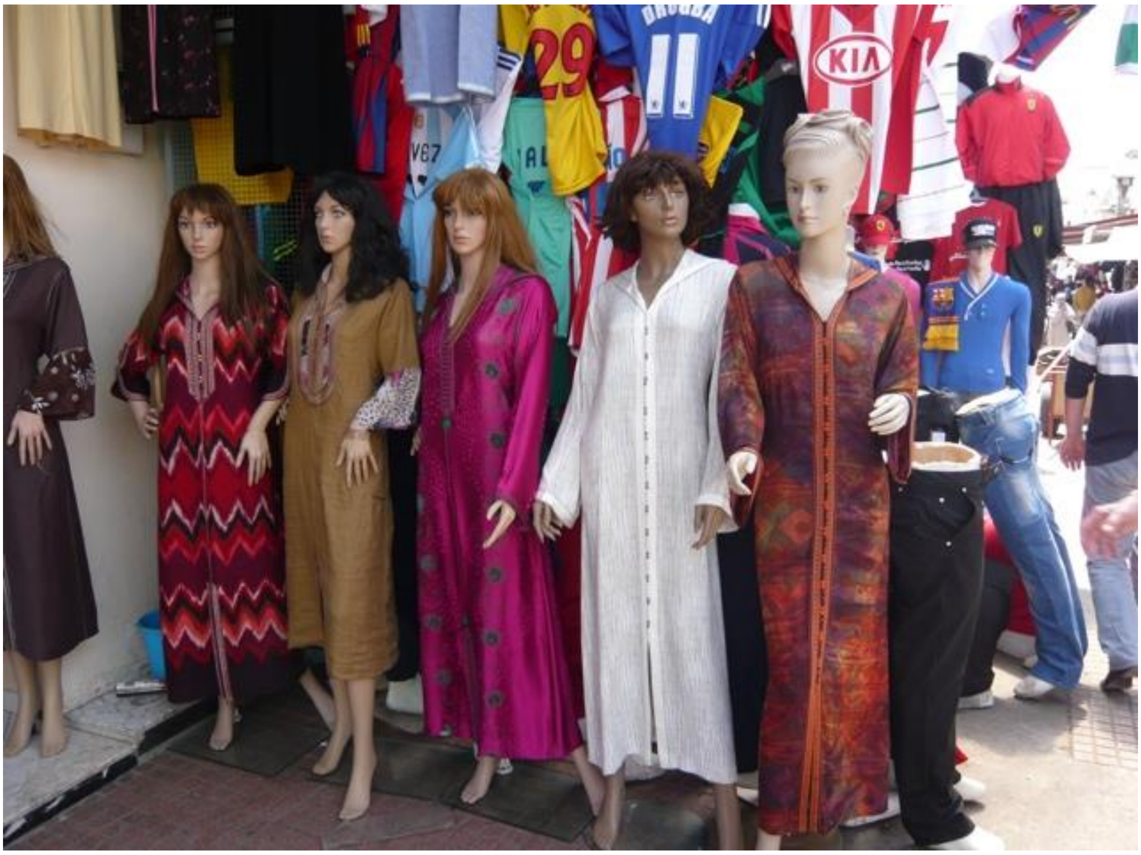

Display #31. Please evaluate this store from 1 to 10.

1	2	3	4	5	6	7	8	9	10

Poorly displayed **Average** **Well displayed**

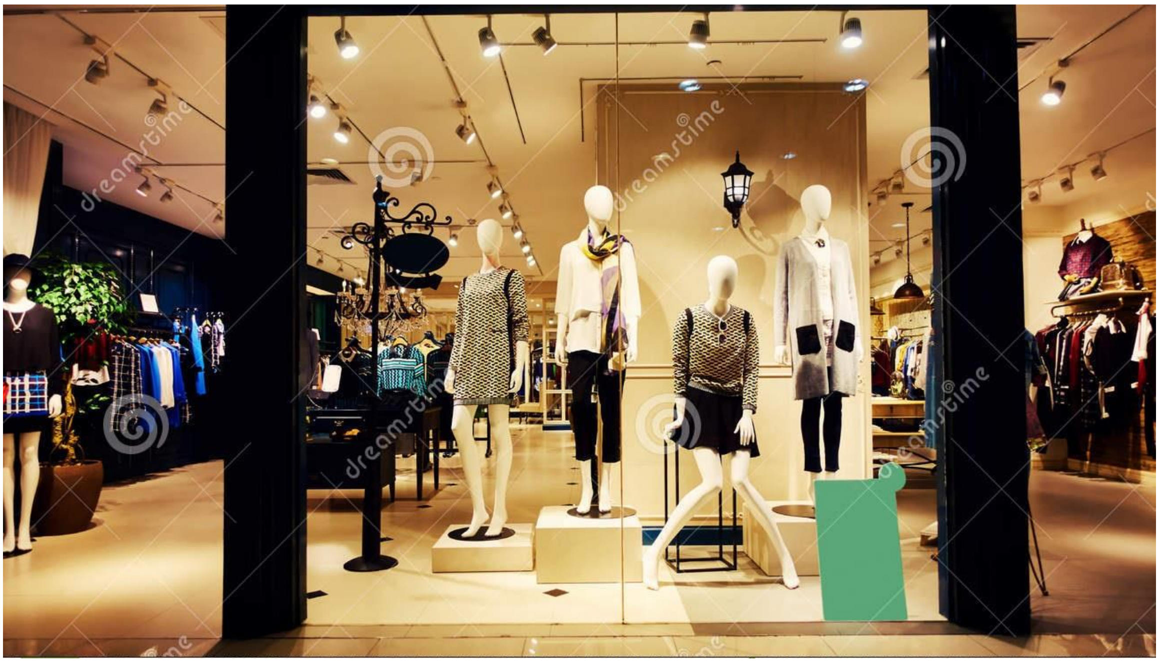

Display #32. Please evaluate this store from 1 to 10.

1	2	3	4	5	6	7	8	9	10

Poorly displayed **Average** **Well displayed**

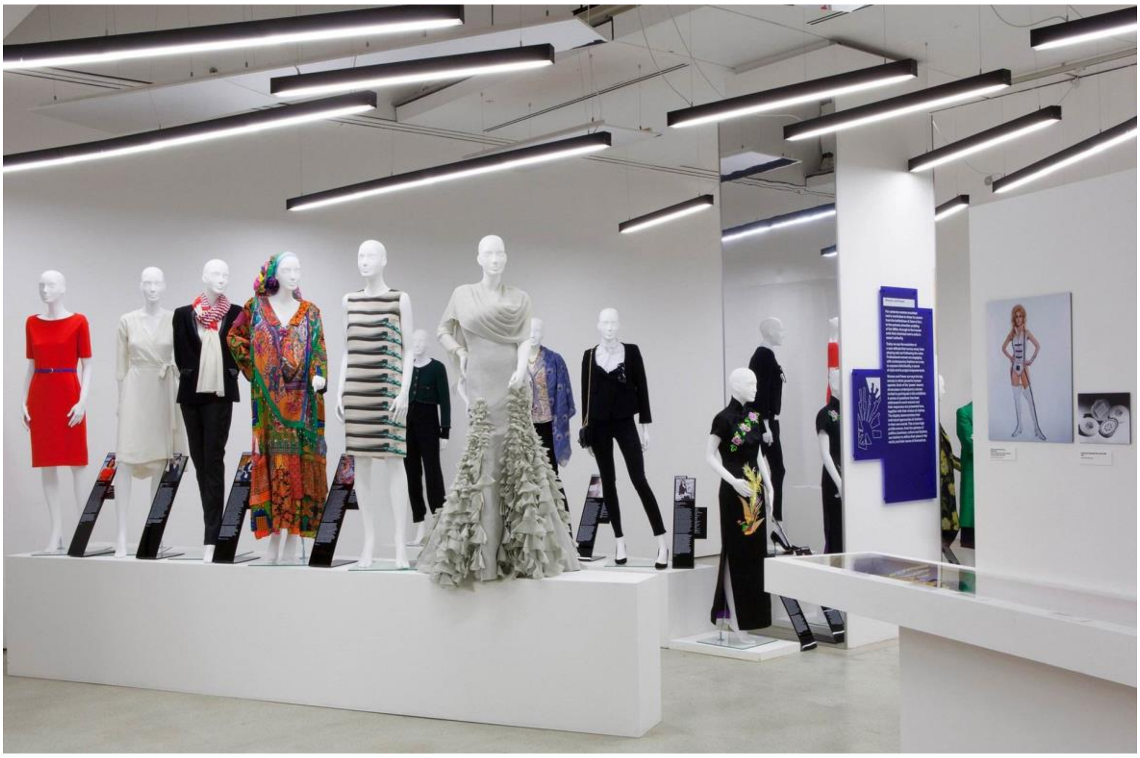

Display #33.   Please evaluate this store from 1 to 10.

1	2	3	4	5	6	7	8	9	10

Poorly displayed **Average** **Well displayed**

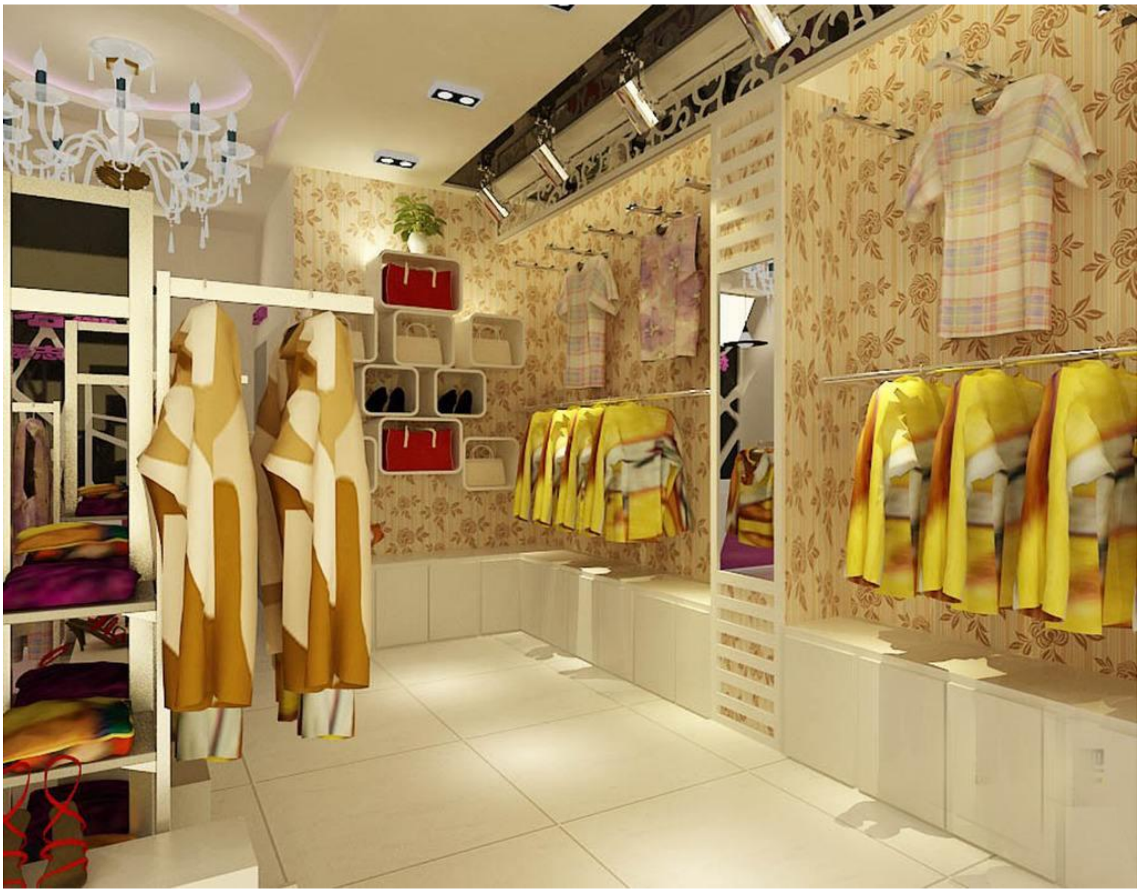

Display #34． Please evaluate this store from 1 to 10.

1	2	3	4	5	6	7	8	9	10

Poorly displayed **Average** **Well displayed**

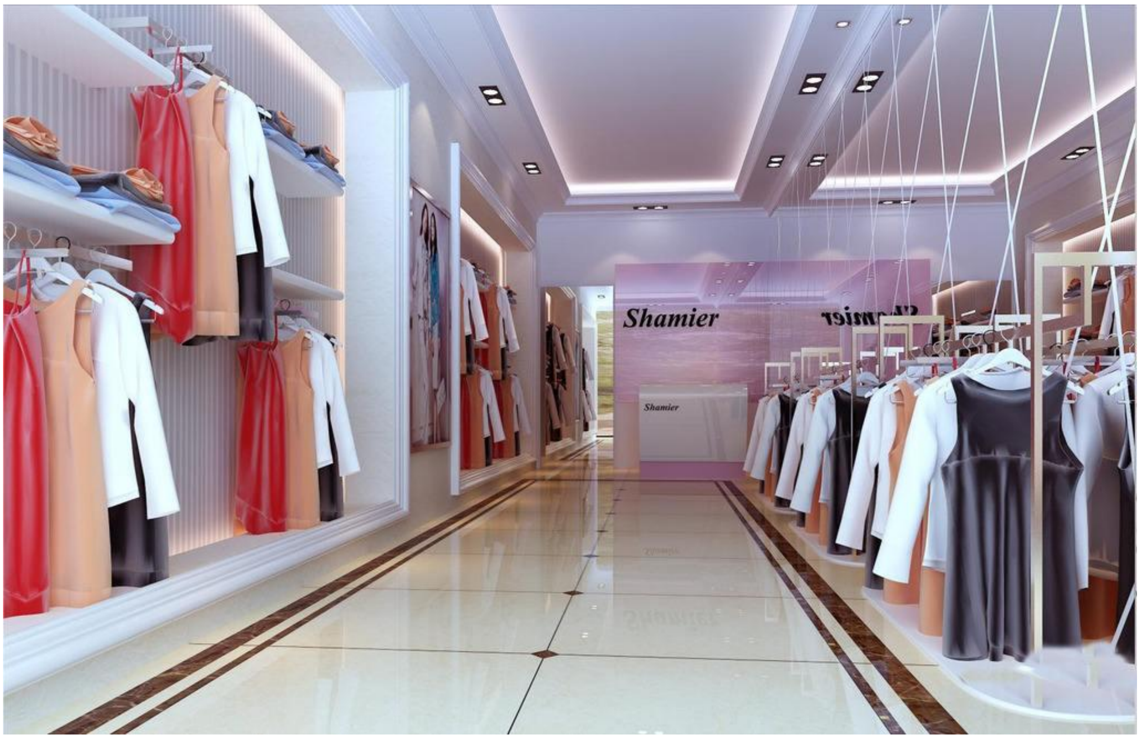

Display #35. Please evaluate this store from 1 to 10.

1	2	3	4	5	6	7	8	9	10

Poorly displayed **Average** **Well displayed**

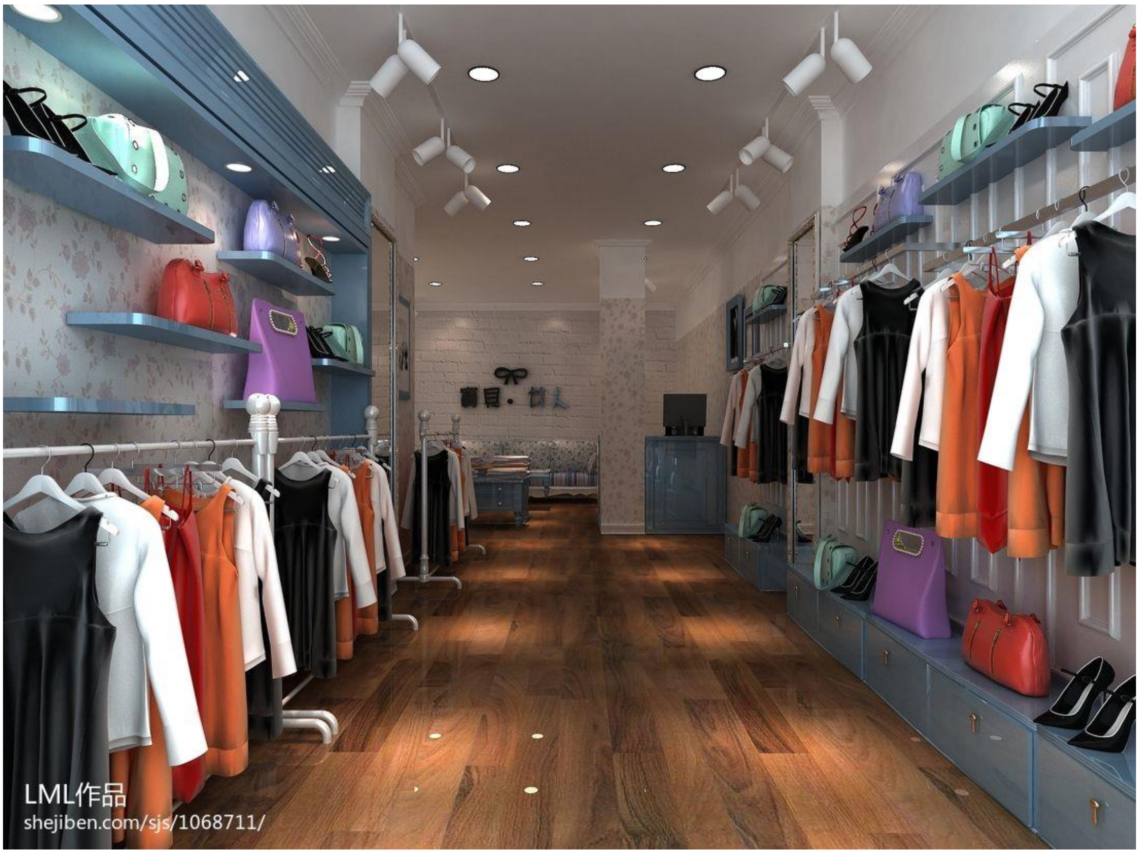

Display #36. Please evaluate this store from 1 to 10.

1	2	3	4	5	6	7	8	9	10

Poorly displayed **Average** **Well displayed**

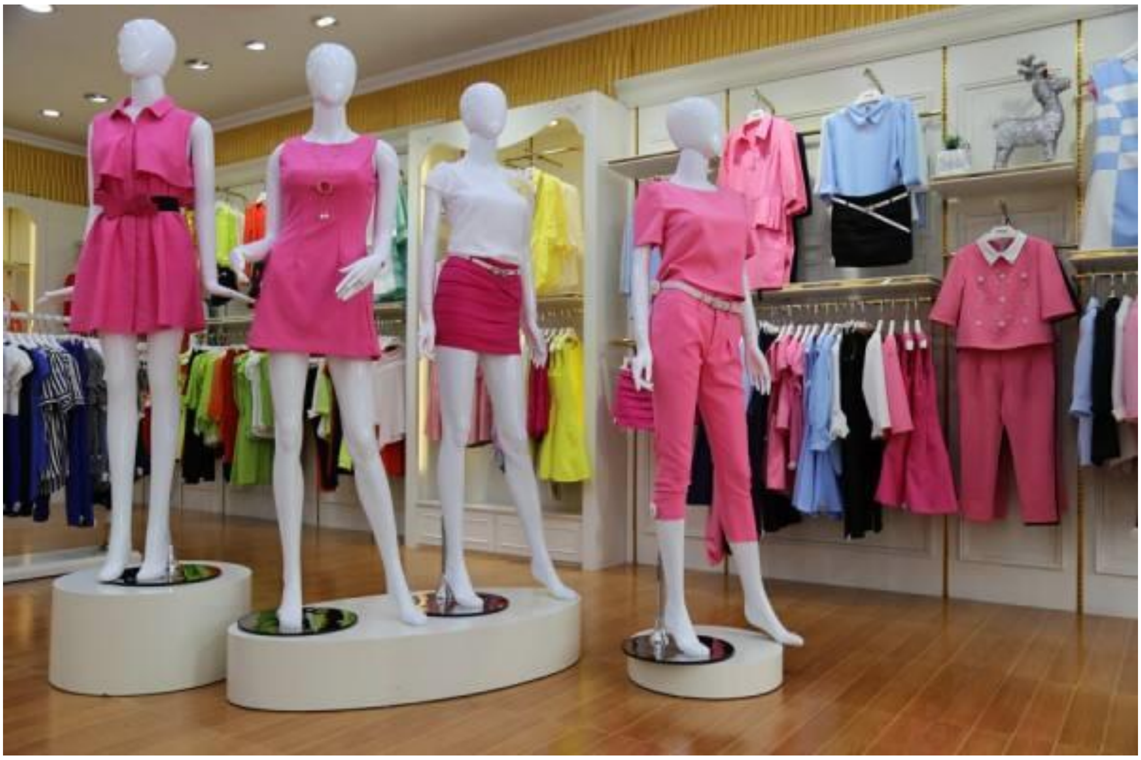

Display #37. Please evaluate this store from 1 to 10.

1	2	3	4	5	6	7	8	9	10

Poorly displayed **Average** **Well displayed**

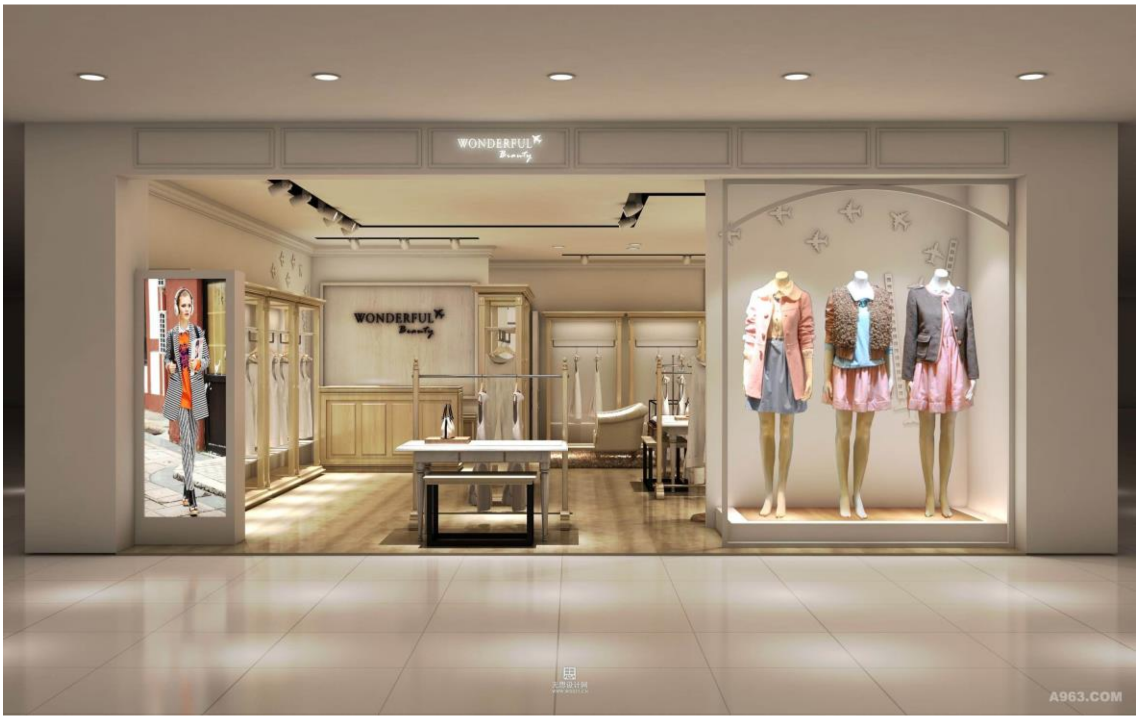

Display #38. Please evaluate this store from 1 to 10.

1	2	3	4	5	6	7	8	9	10

Poorly displayed **Average** **Well displayed**

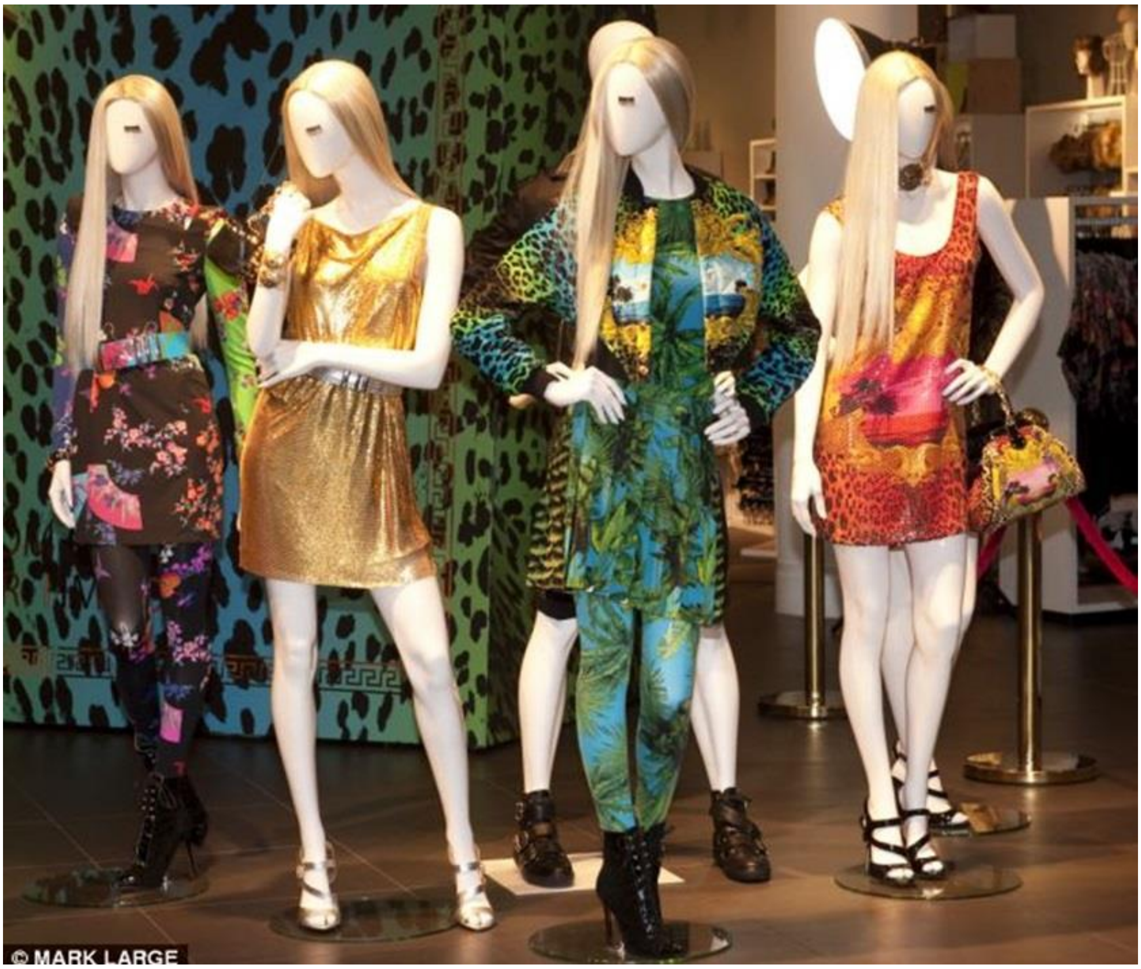

Display #39. Please evaluate this store from 1 to 10.

1	2	3	4	5	6	7	8	9	10

Poorly displayed **Average** **Well displayed**

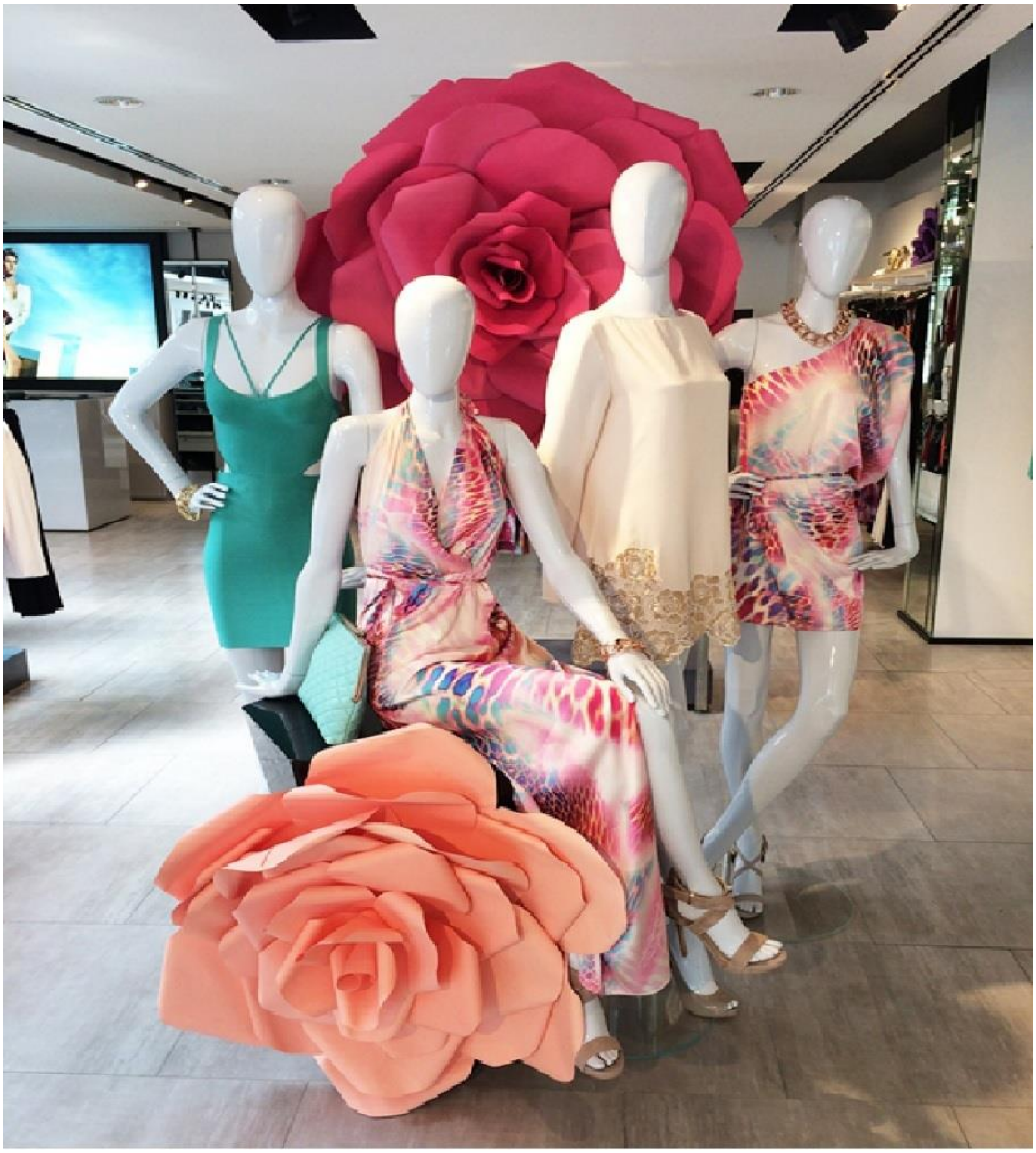

Display #40. Please evaluate this store from 1 to 10.

1	2	3	4	5	6	7	8	9	10

Poorly displayed **Average** **Well displayed**

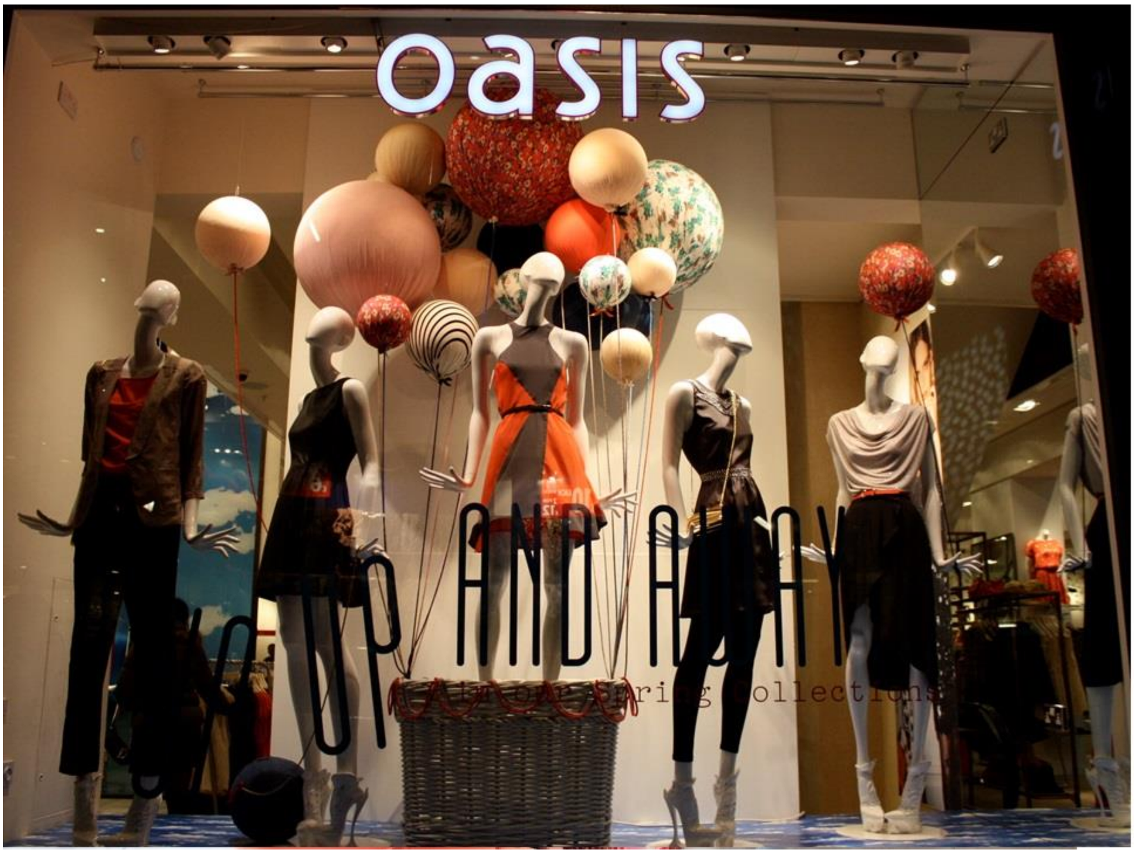

Display #41． Please evaluate this store from 1 to 10.

1	2	3	4	5	6	7	8	9	10

Poorly displayed **Average** **Well displayed**

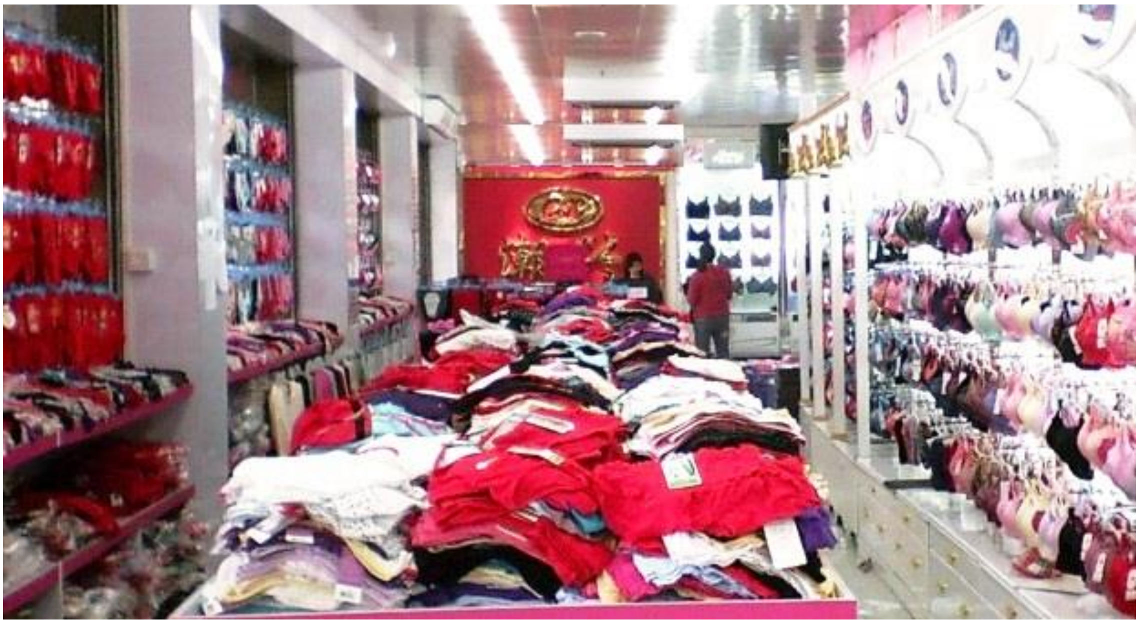

Display #42. Please evaluate this store from 1 to 10.

1	2	3	4	5	6	7	8	9	10

Poorly displayed **Average** **Well displayed**

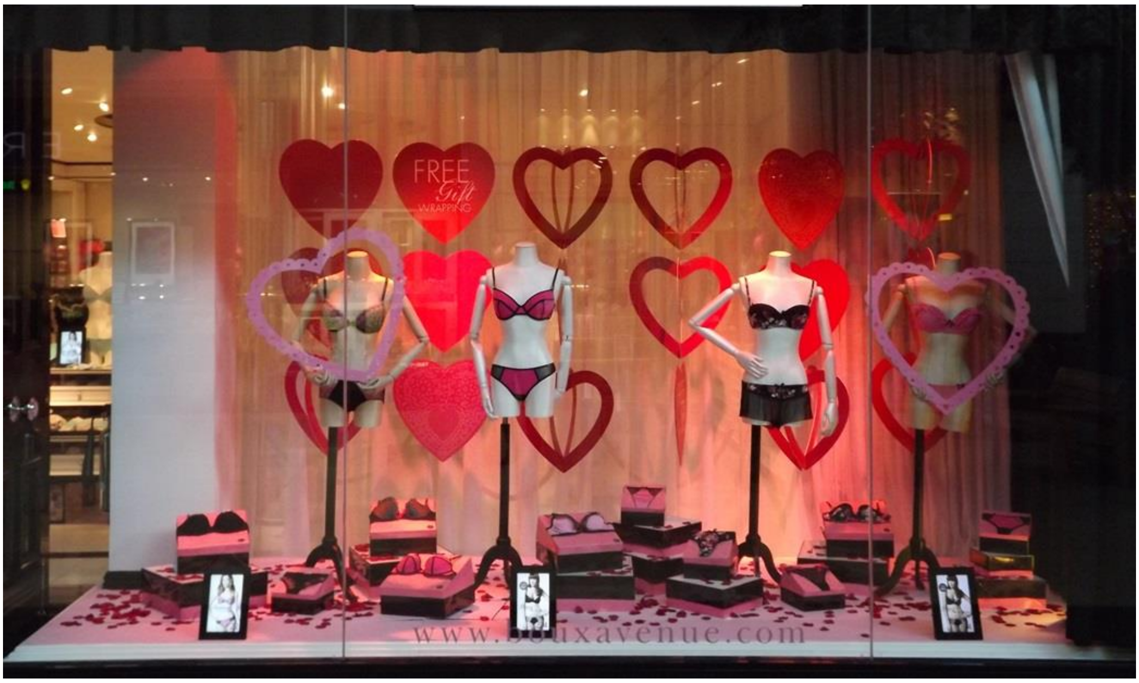

Display #43. Please evaluate this store from 1 to 10.

1	2	3	4	5	6	7	8	9	10

Poorly displayed **Average** **Well displayed**

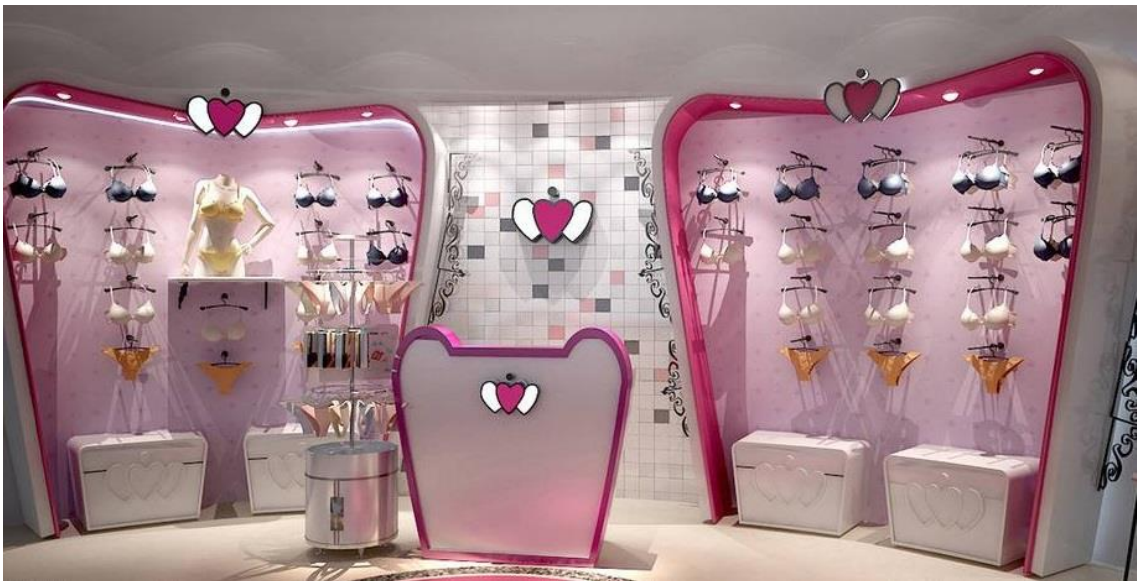

Display #44. Please evaluate this store from 1 to 10.

1	2	3	4	5	6	7	8	9	10

Poorly displayed **Average** **Well displayed**

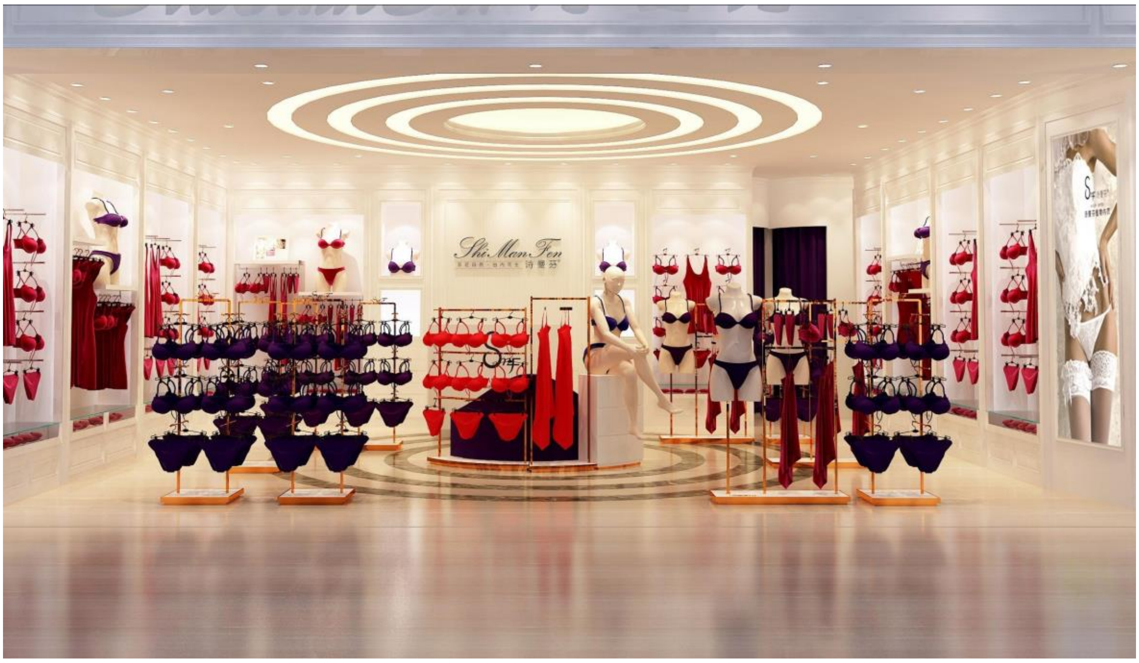

Display #45. Please evaluate this store from 1 to 10.

1	2	3	4	5	6	7	8	9	10

Poorly displayed **Average** **Well displayed**

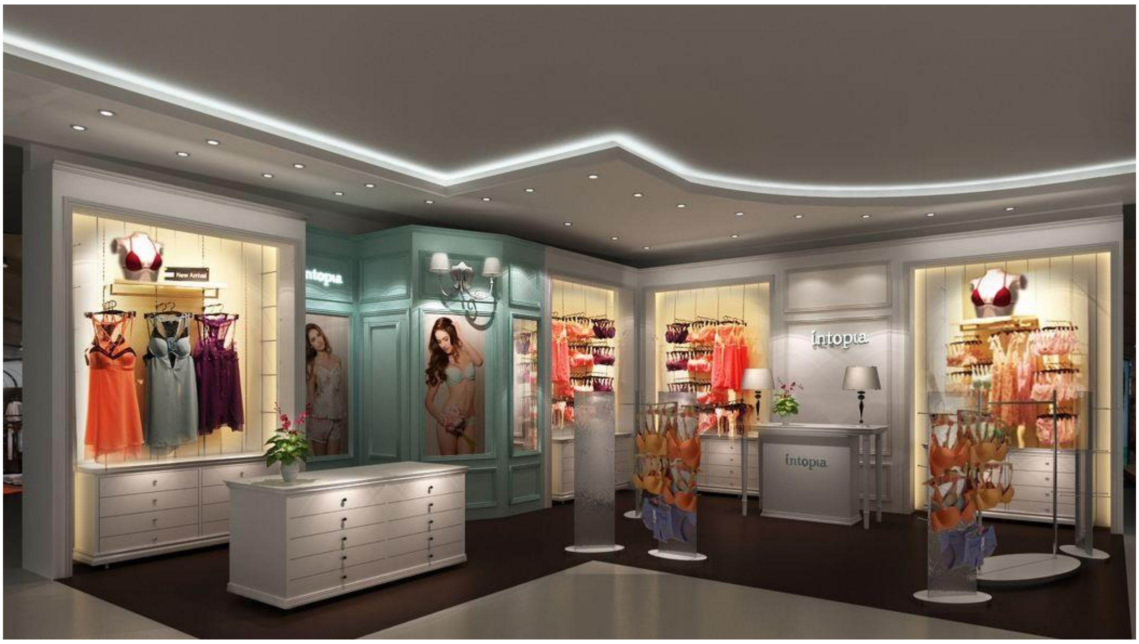

Display #46. Please evaluate this store from 1 to 10.

1	2	3	4	5	6	7	8	9	10

Poorly displayed **Average** **Well displayed**

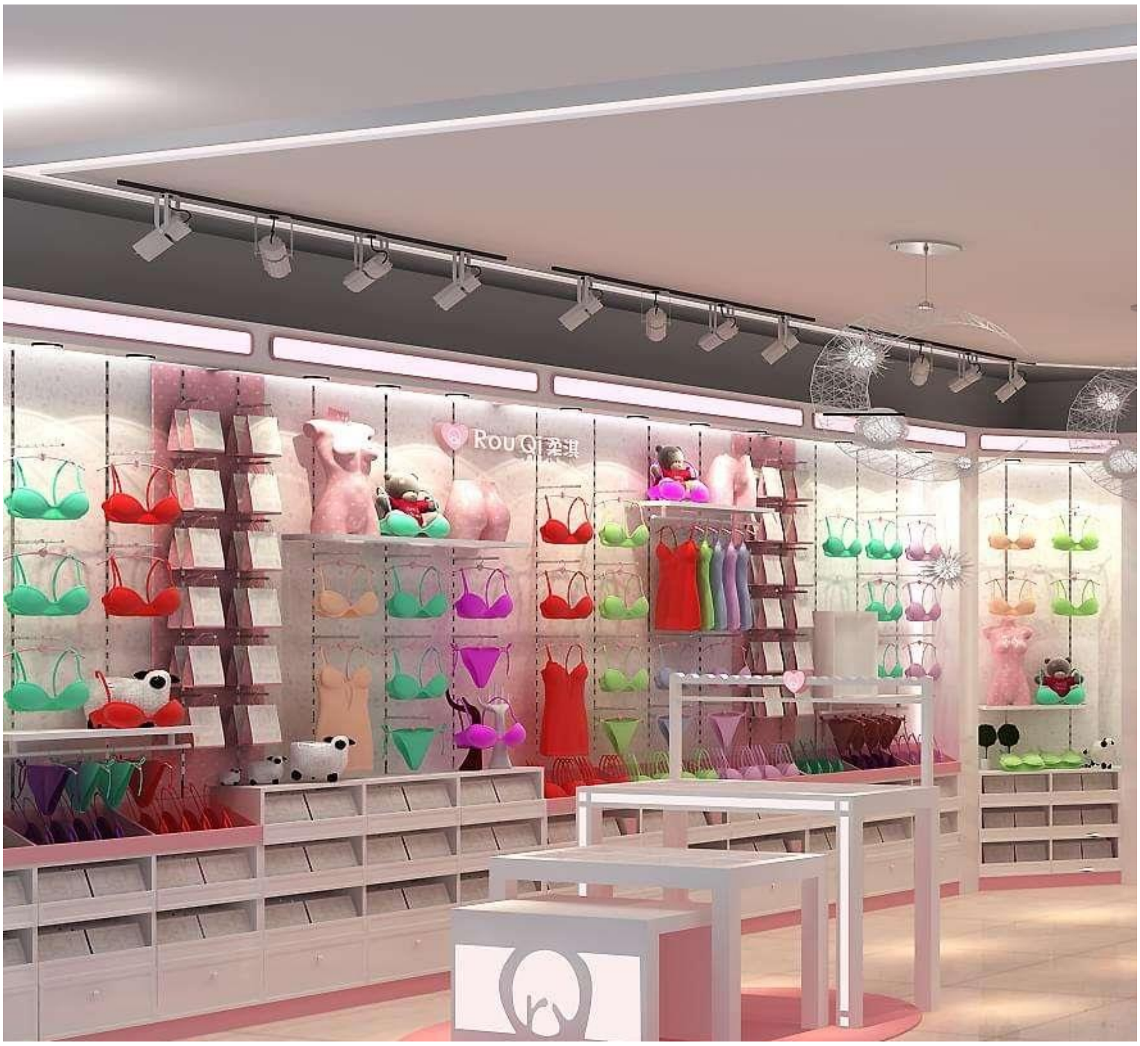

Display #47. Please evaluate this store from 1 to 10.

1	2	3	4	5	6	7	8	9	10

Poorly displayed **Average** **Well displayed**

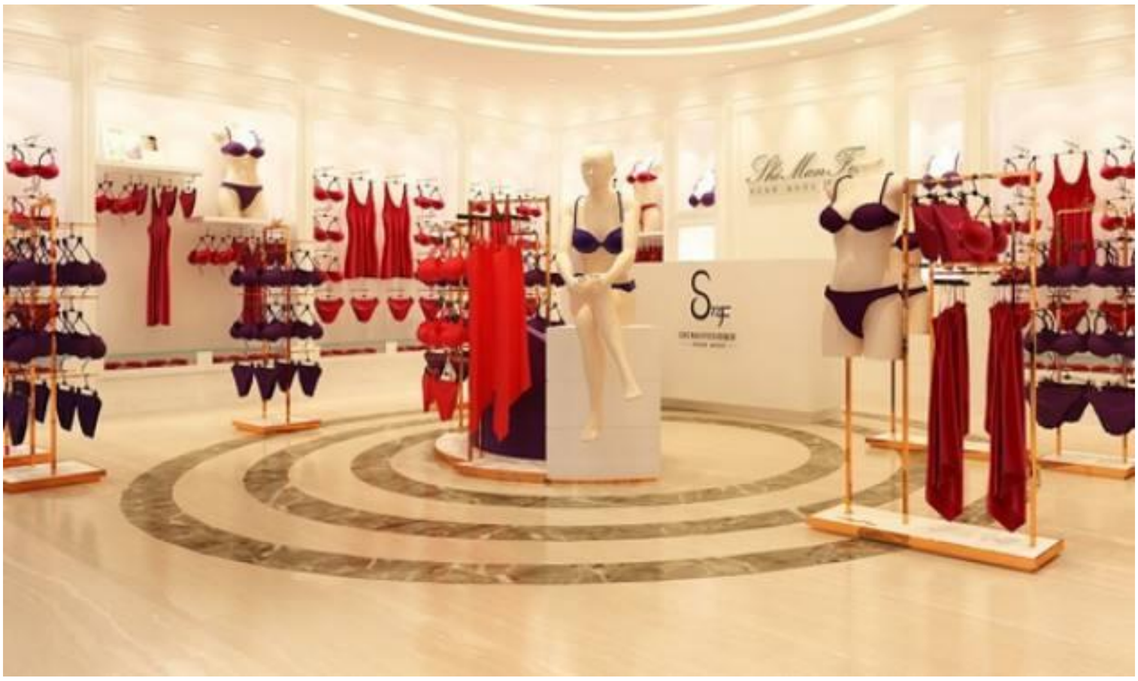

Display #48． Please evaluate this store from 1 to 10.

1	2	3	4	5	6	7	8	9	10

Poorly displayed **Average** **Well displayed**

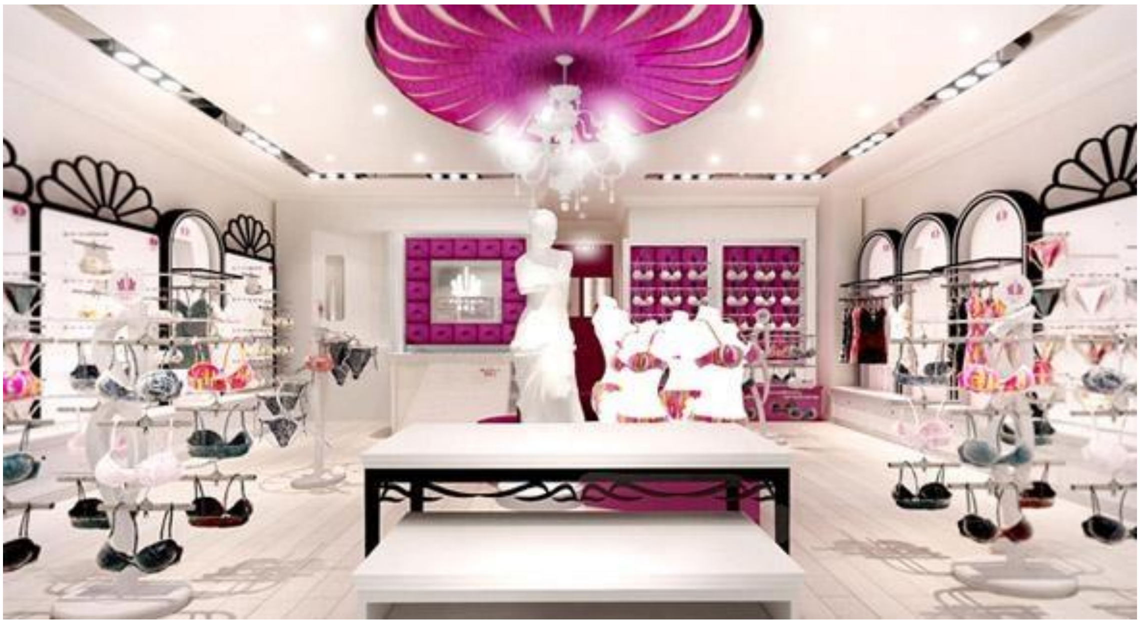

Display #49.   Please evaluate this store from 1 to 10.

1	2	3	4	5	6	7	8	9	10

Poorly displayed **Average** **Well displayed**

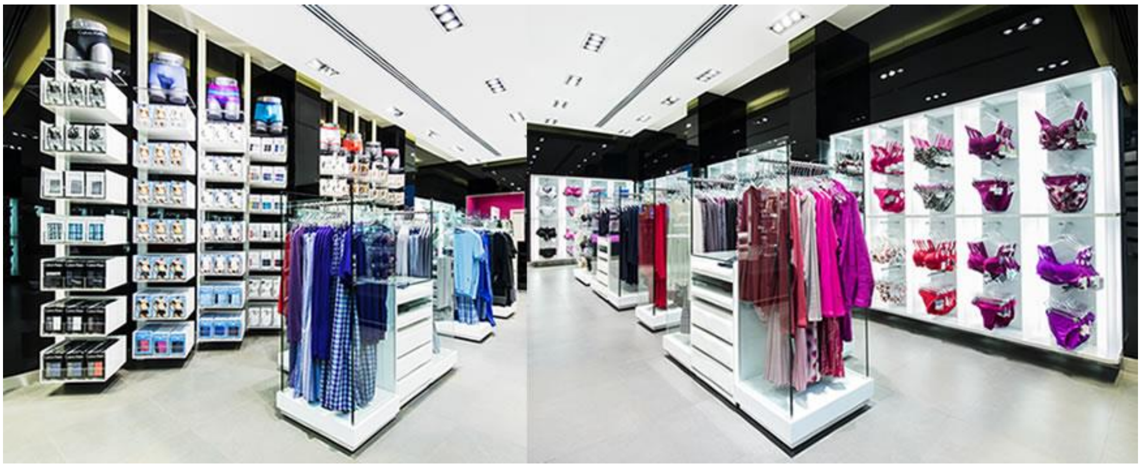

Display #50． Please evaluate this store from 1 to 10.

1	2	3	4	5	6	7	8	9	10

Poorly displayed **Average** **Well displayed**

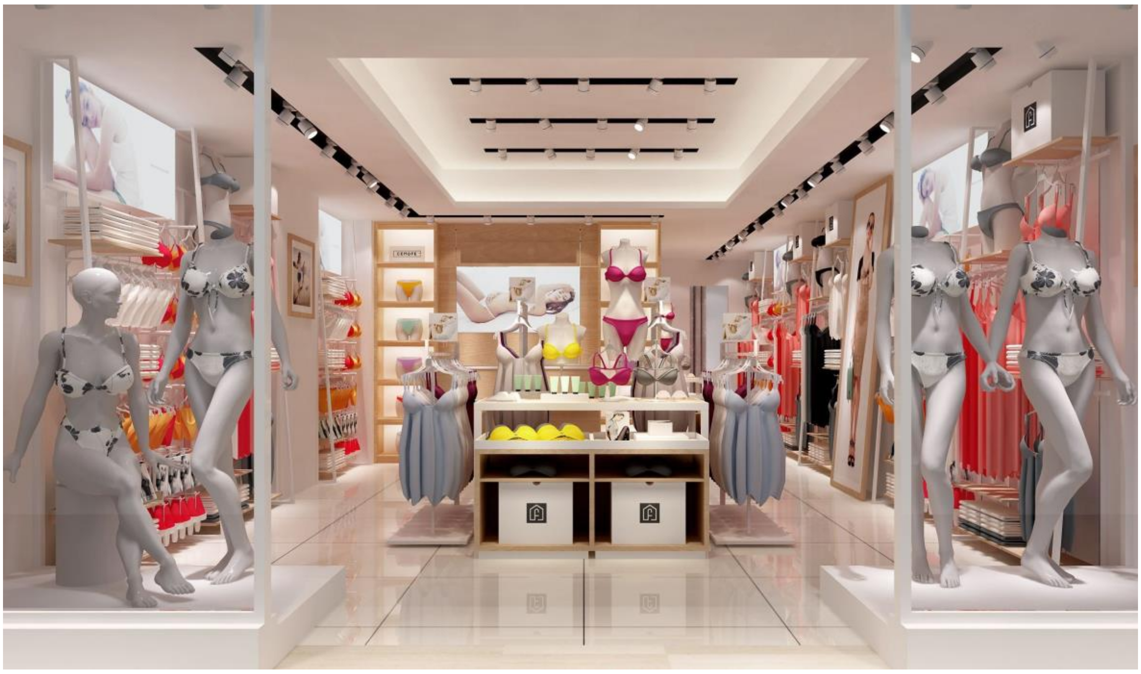

Display #51. Please evaluate this store from 1 to 10.

1	2	3	4	5	6	7	8	9	10

Poorly displayed **Average** **Well displayed**

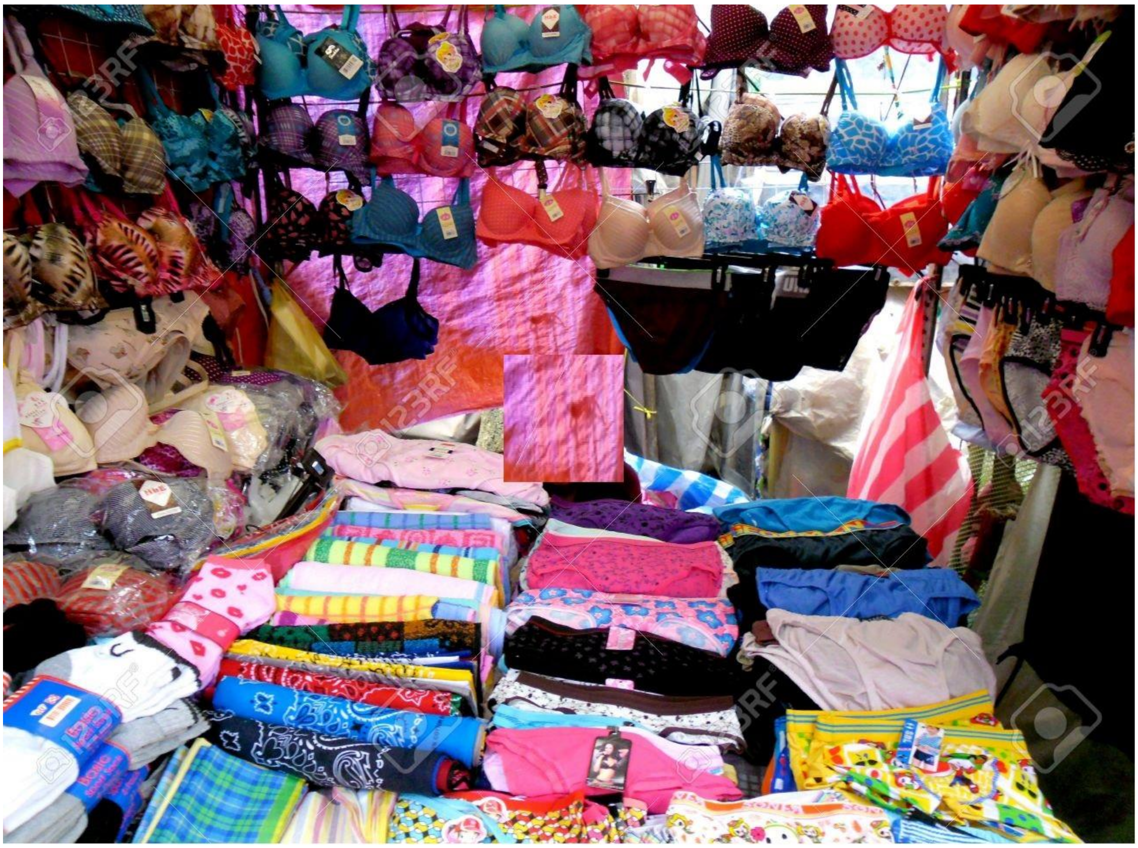

Display #52. Please evaluate this store from 1 to 10.

1	2	3	4	5	6	7	8	9	10

Poorly displayed **Average** **Well displayed**

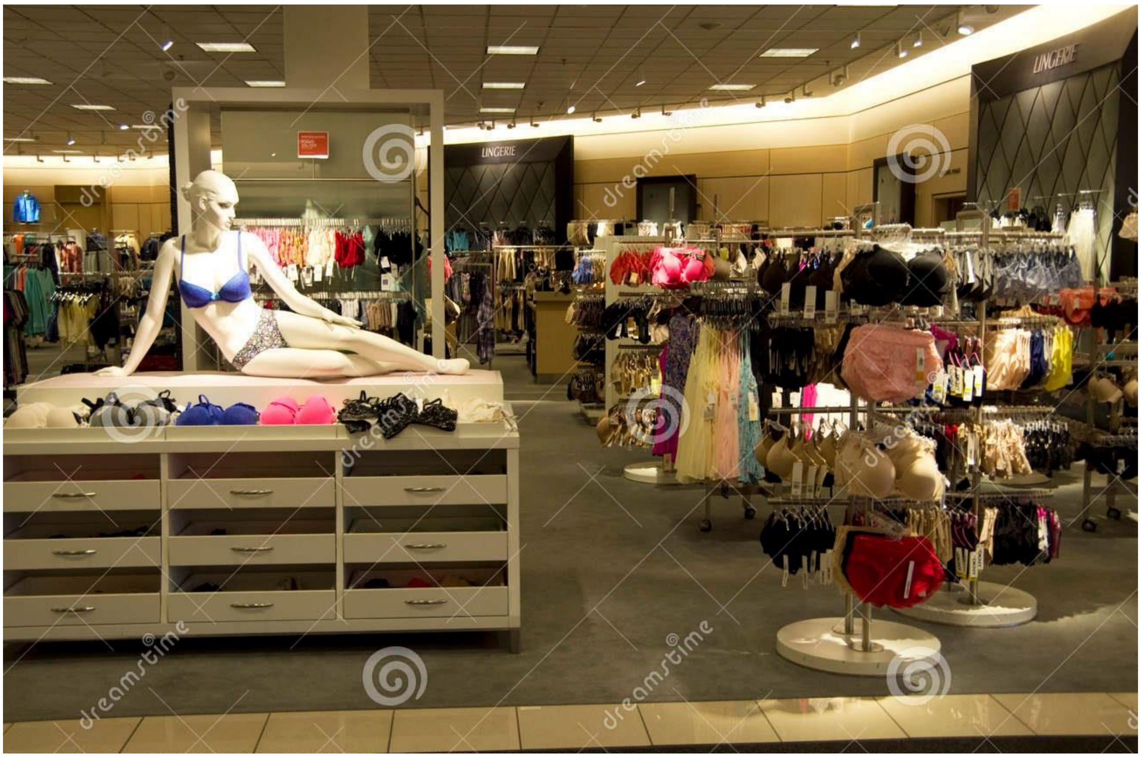

Display #53. Please evaluate this store from 1 to 10.

1	2	3	4	5	6	7	8	9	10

Poorly displayed **Average** **Well displayed**

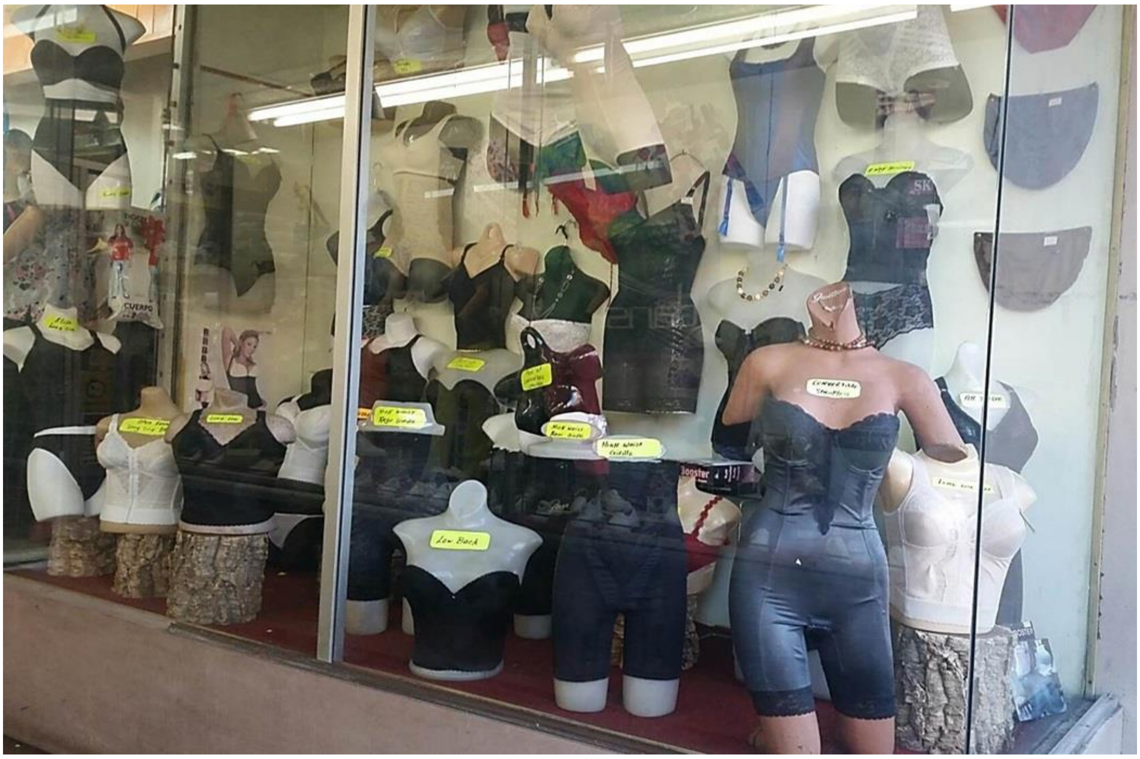

Display #54. Please evaluate this store from 1 to 10.

1	2	3	4	5	6	7	8	9	10

Poorly displayed **Average** **Well displayed**

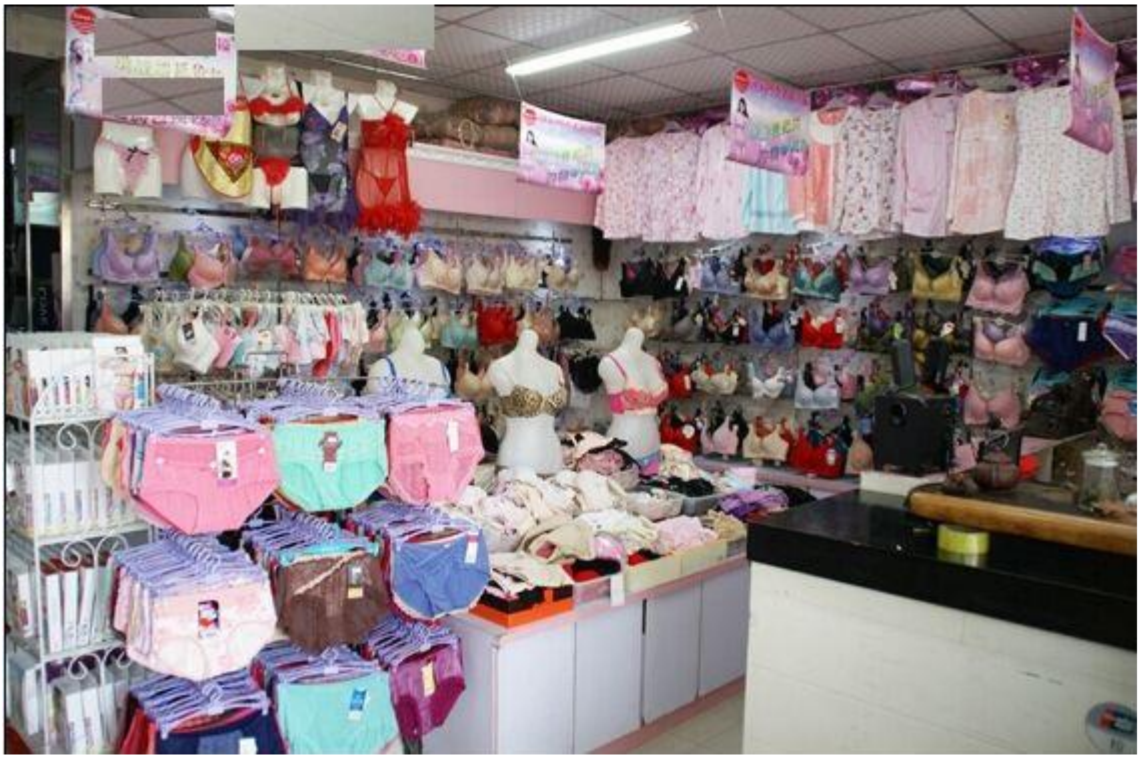

Display #55. Please evaluate this store from 1 to 10.

1	2	3	4	5	6	7	8	9	10

Poorly displayed **Average** **Well displayed**

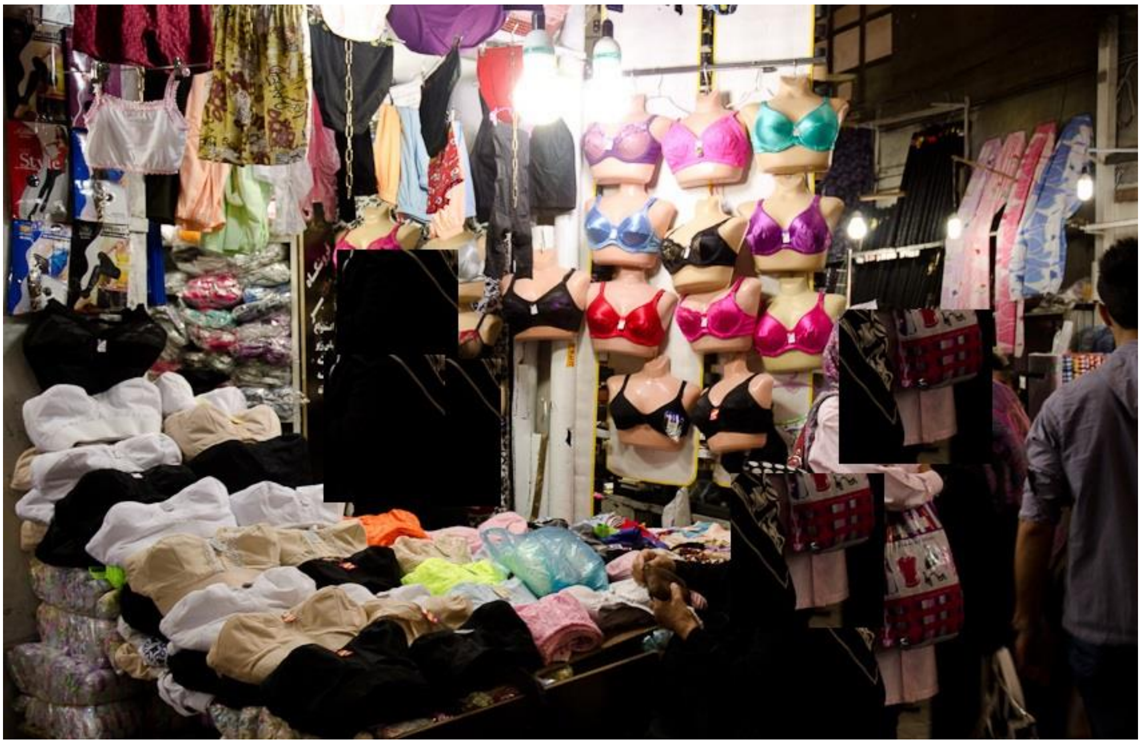

Display #56. Please evaluate this store from 1 to 10.

1	2	3	4	5	6	7	8	9	10

Poorly displayed **Average** **Well displayed**

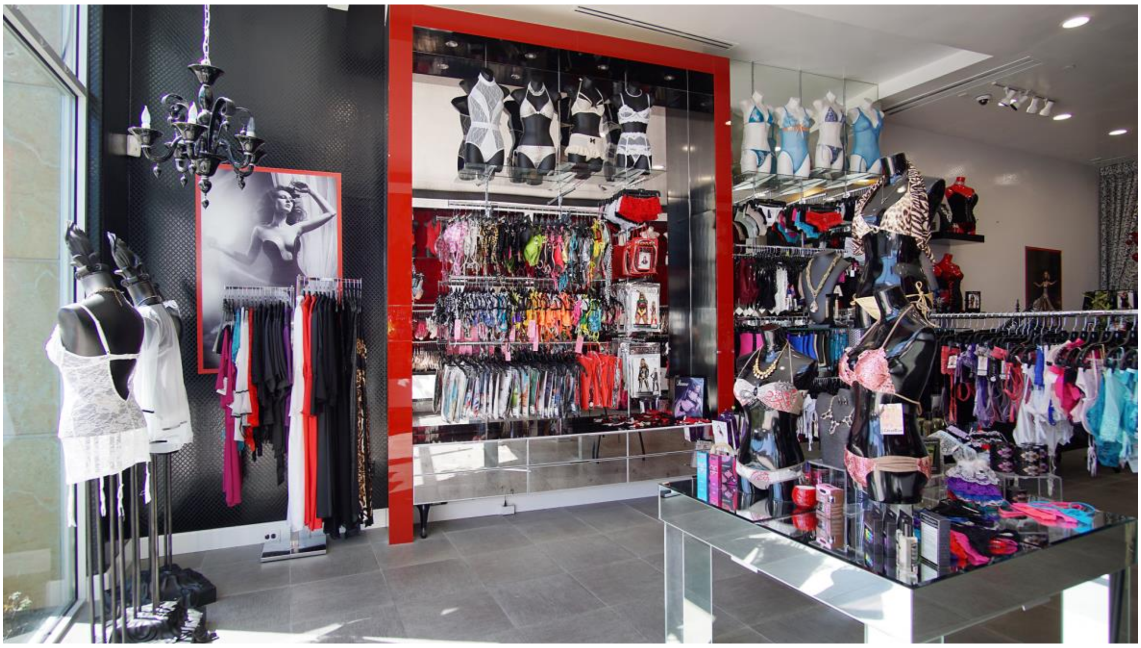

Display #57. Please evaluate this store from 1 to 10.

1	2	3	4	5	6	7	8	9	10

Poorly displayed **Average** **Well displayed**

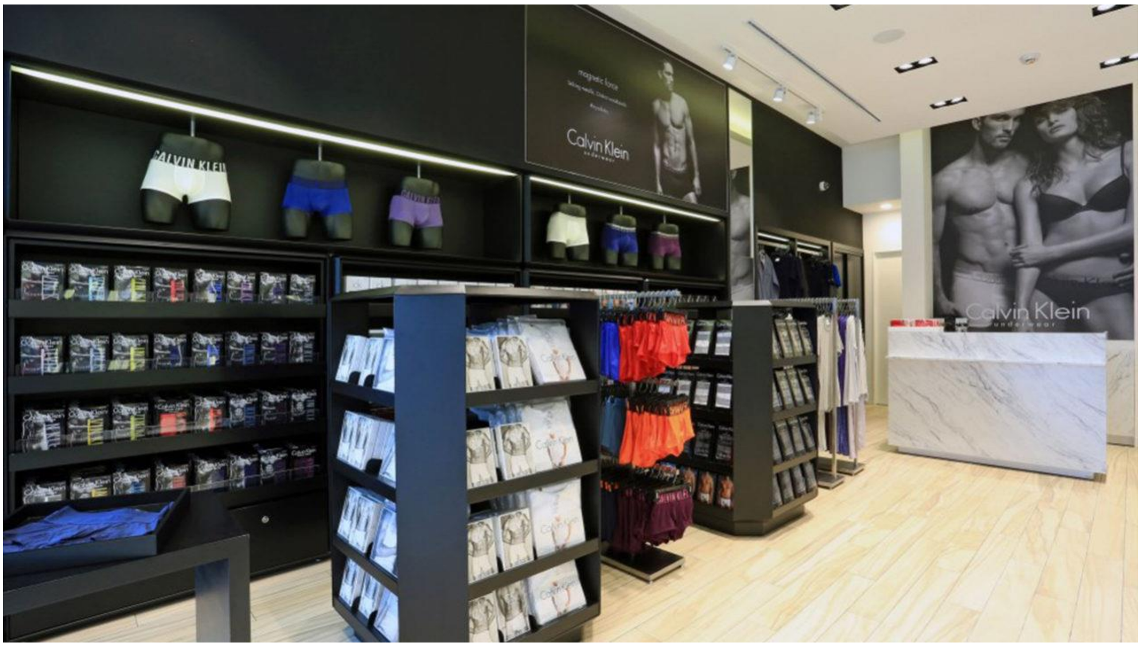

Display #58． Please evaluate this store from 1 to 10.

1	2	3	4	5	6	7	8	9	10

Poorly displayed **Average** **Well displayed**

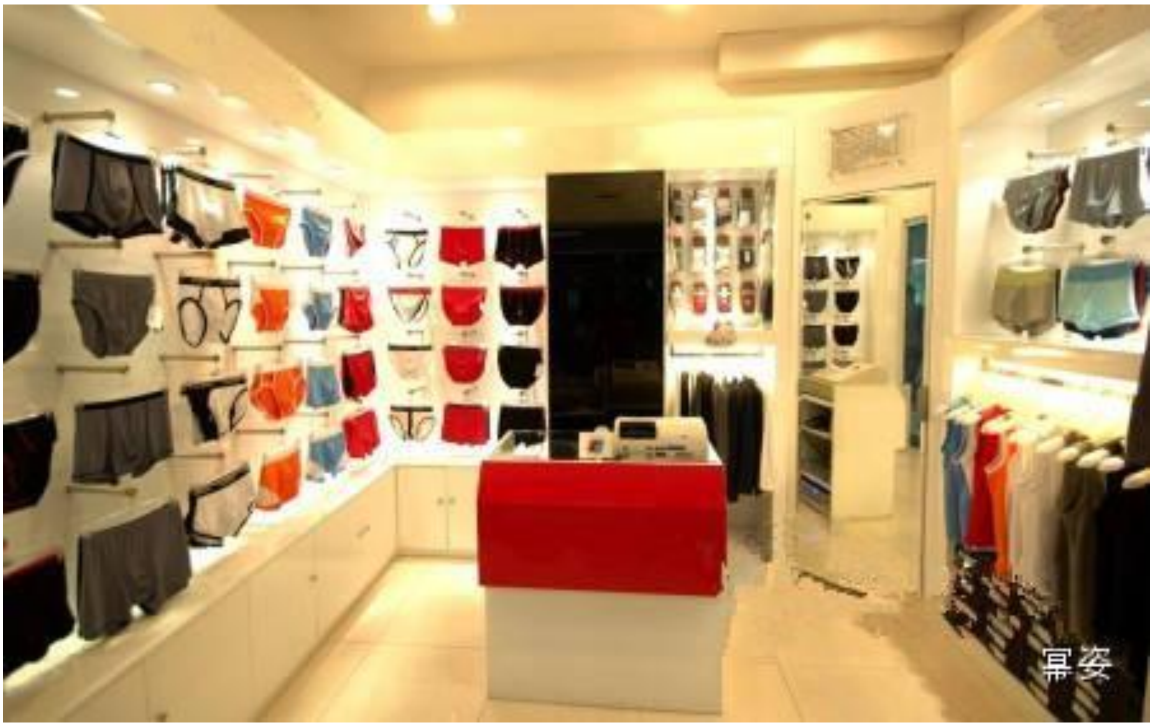

Display #59. Please evaluate this store from 1 to 10.

1	2	3	4	5	6	7	8	9	10

Poorly displayed **Average** **Well displayed**

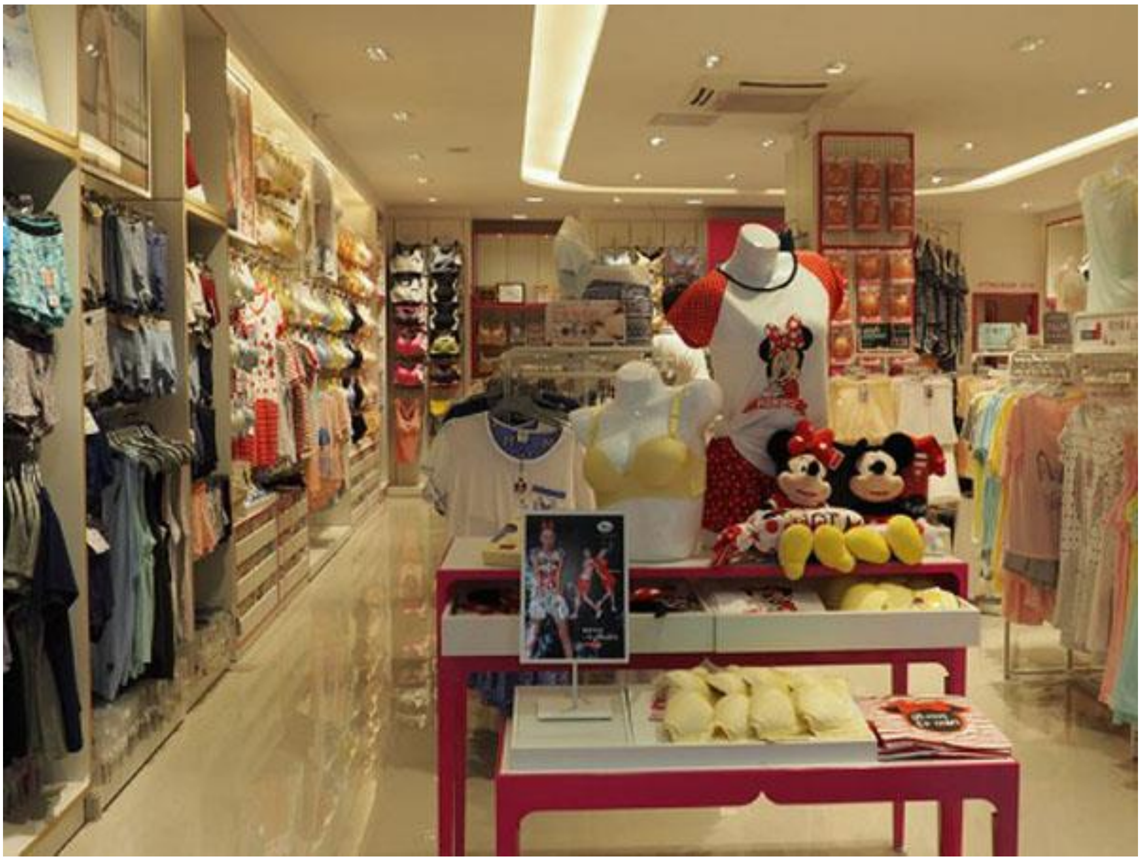

Display #60． Please evaluate this store from 1 to 10.

1	2	3	4	5	6	7	8	9	10

Poorly displayed **Average** **Well displayed**

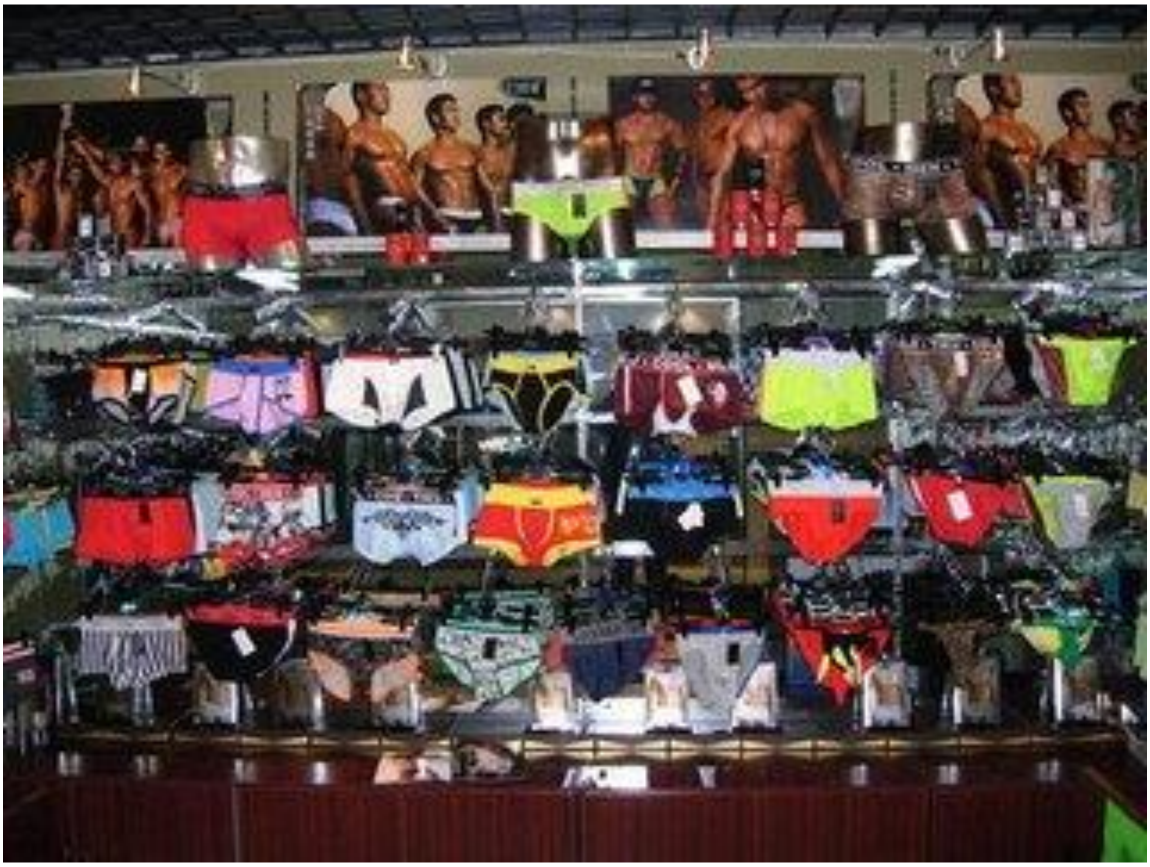

Display #61. Please evaluate this store from 1 to 10.

1	2	3	4	5	6	7	8	9	10

Poorly displayed **Average** **Well displayed**

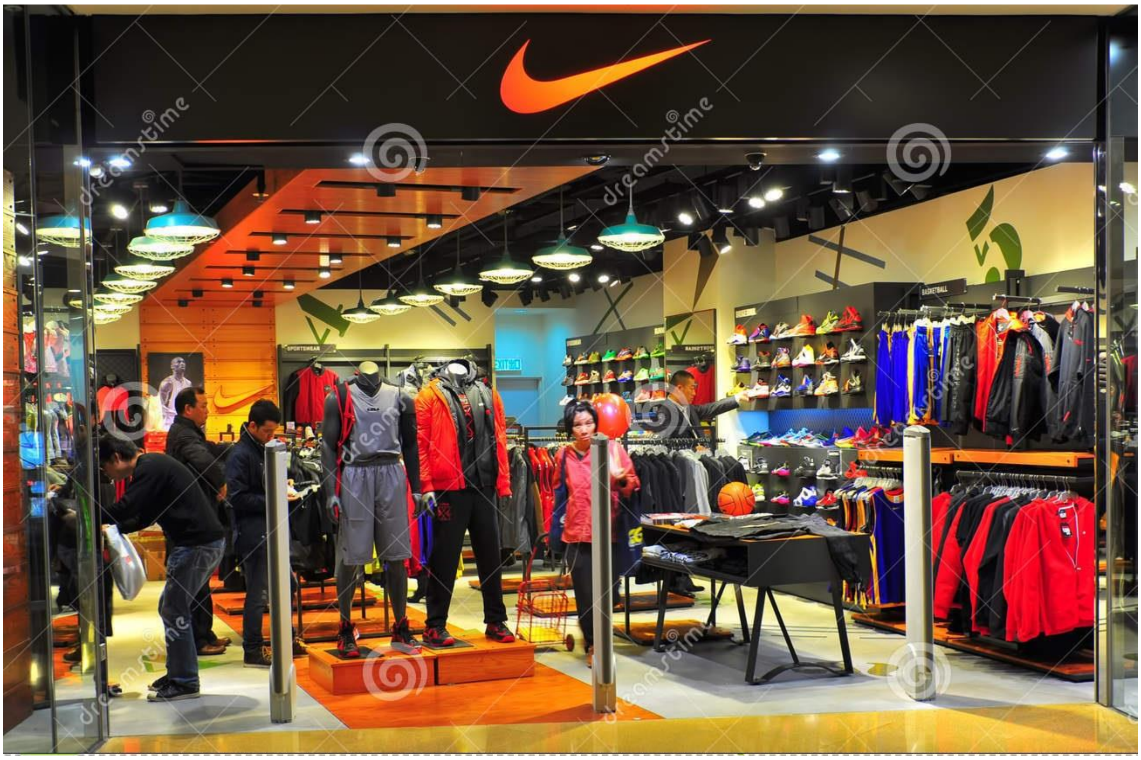

Display #62. **Please evaluate this store from 1 to 10.**

1	2	3	4	5	6	7	8	9	10

Poorly displayed **Average** **Well displayed**

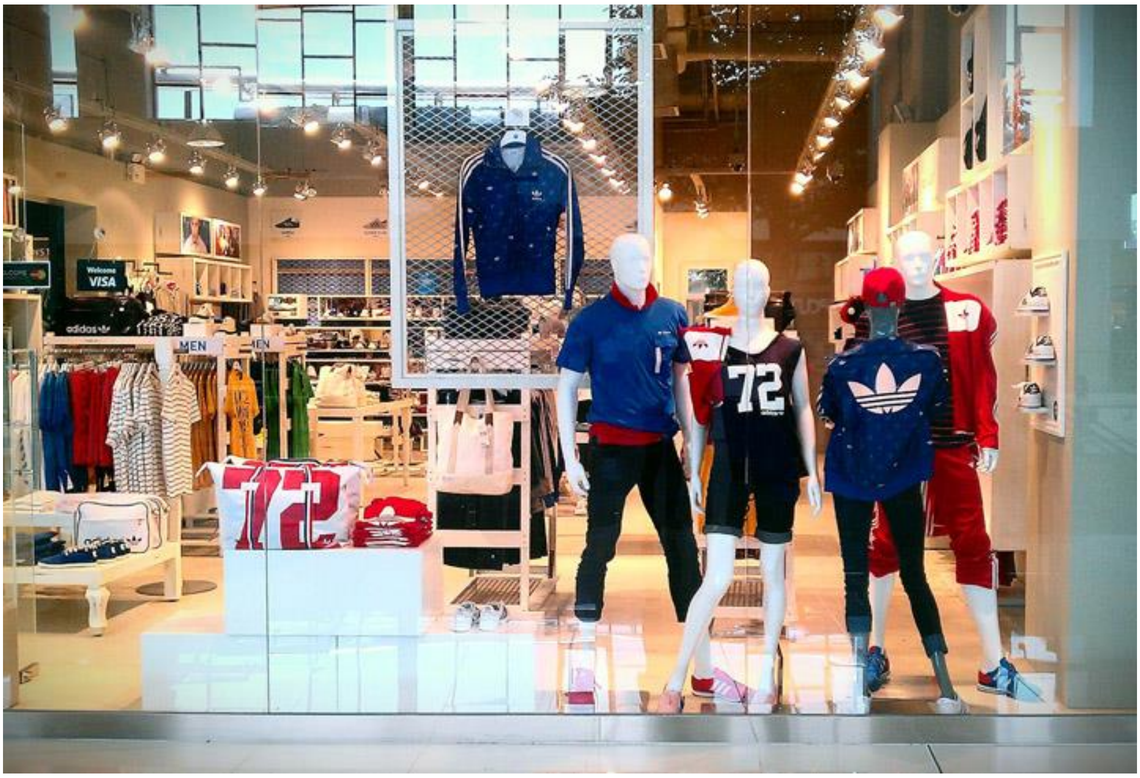

Display #63. Please evaluate this store from 1 to 10.

1	2	3	4	5	6	7	8	9	10

Poorly displayed **Average** **Well displayed**

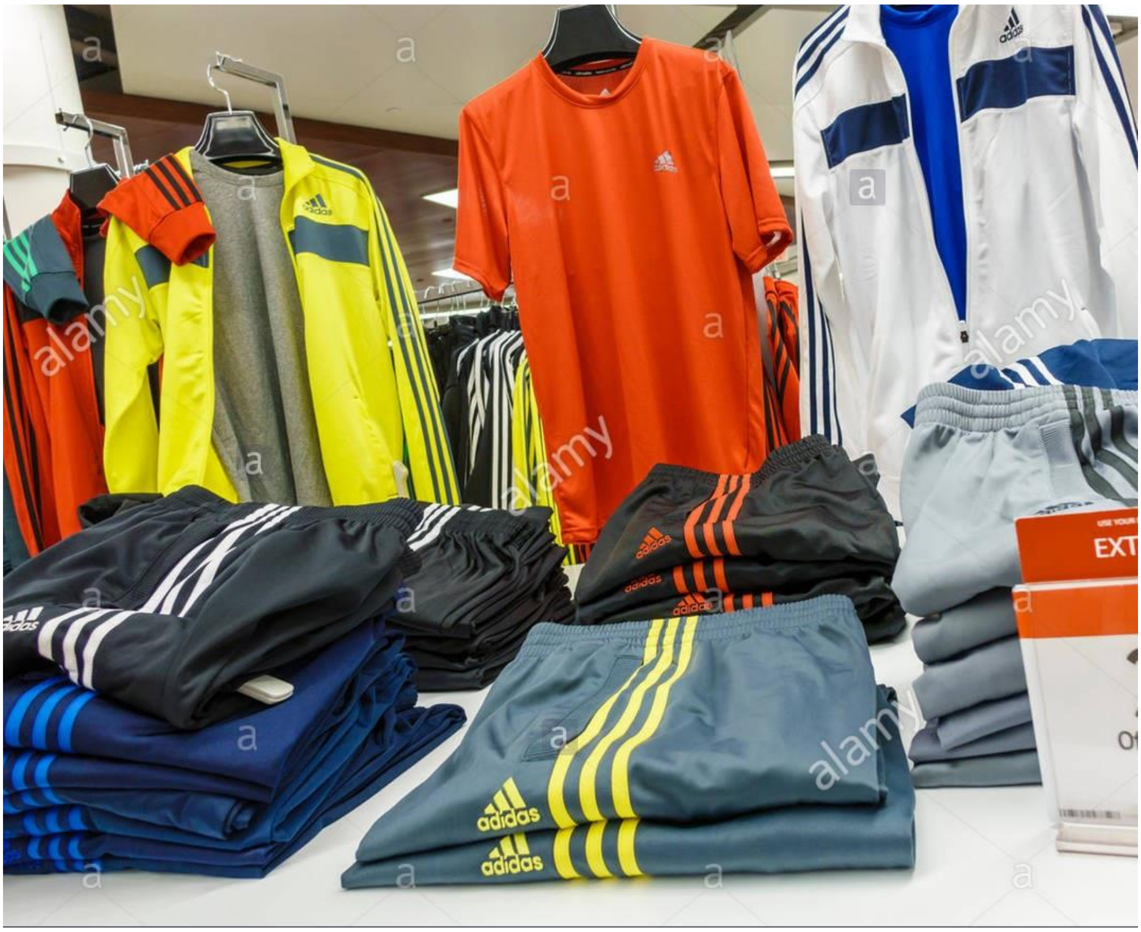

Display #64. Please evaluate this store from 1 to 10.

1	2	3	4	5	6	7	8	9	10

Poorly displayed **Average** **Well displayed**

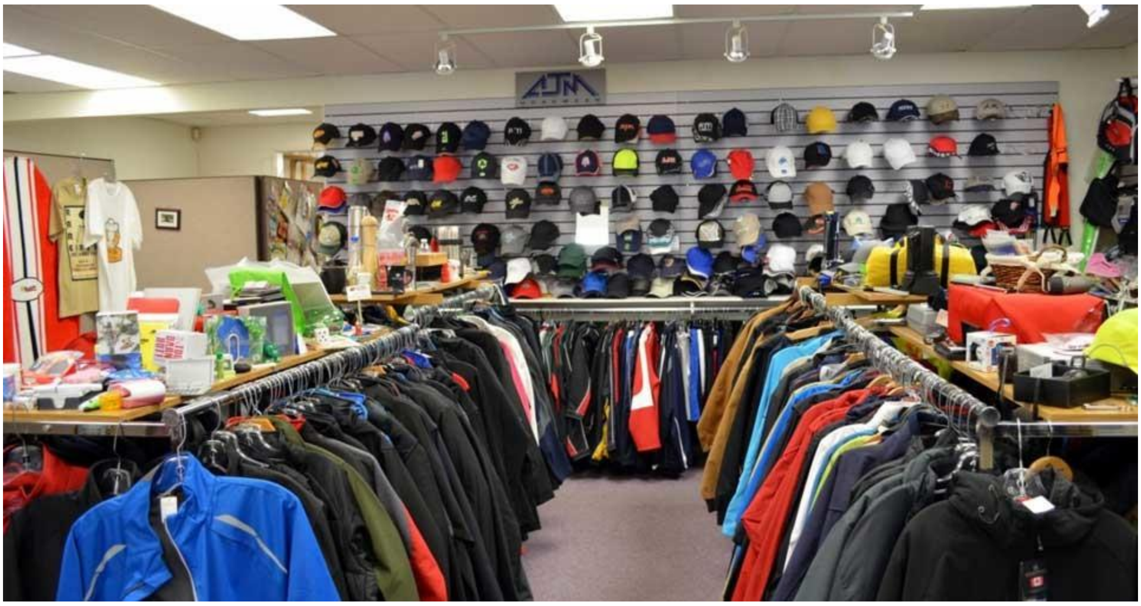

Display #65. Please evaluate this store from 1 to 10.

1	2	3	4	5	6	7	8	9	10

Poorly displayed **Average** **Well displayed**

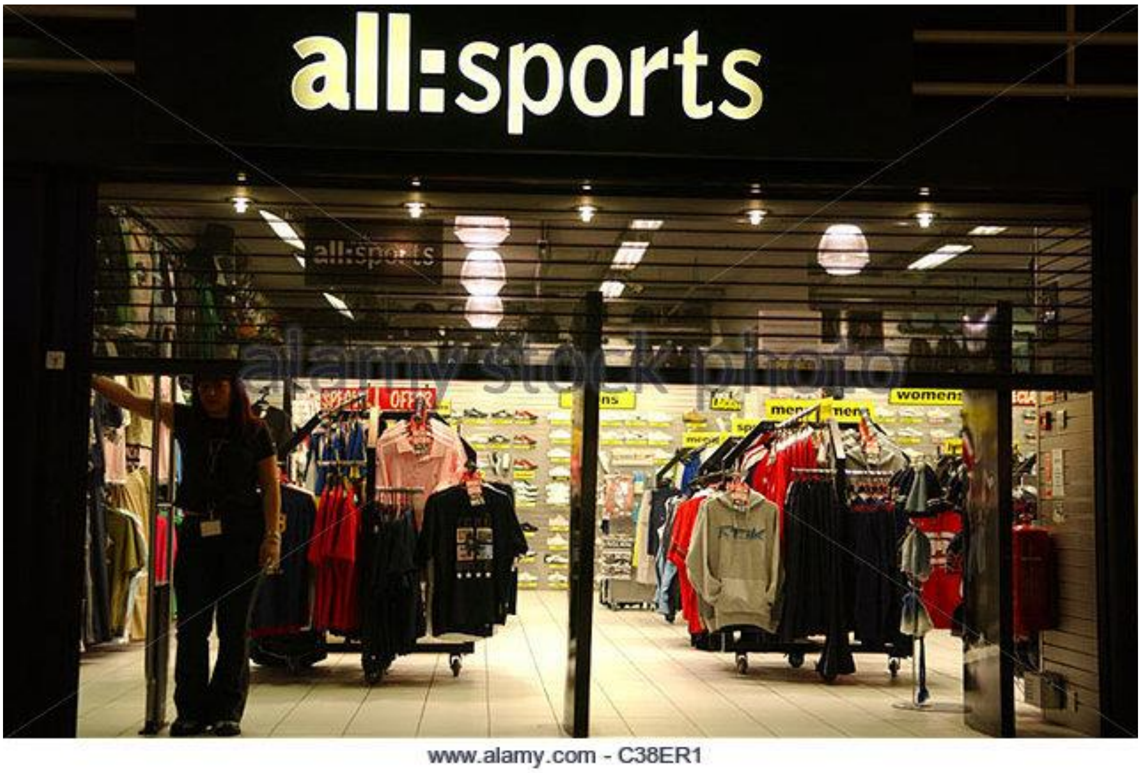

Display #66． Please evaluate this store from 1 to 10.

1	2	3	4	5	6	7	8	9	10

Poorly displayed **Average** **Well displayed**

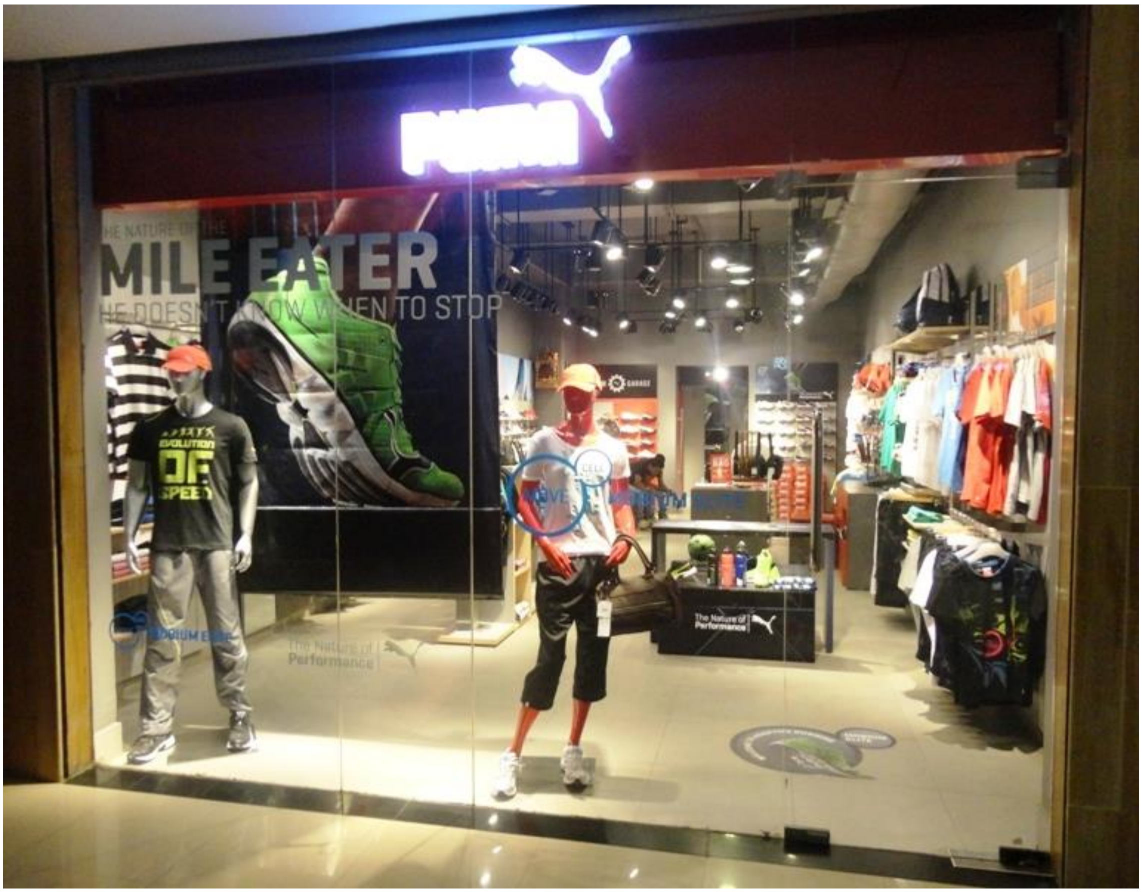

Display #67. Please evaluate this store from 1 to 10.

1	2	3	4	5	6	7	8	9	10

Poorly displayed **Average** **Well displayed**

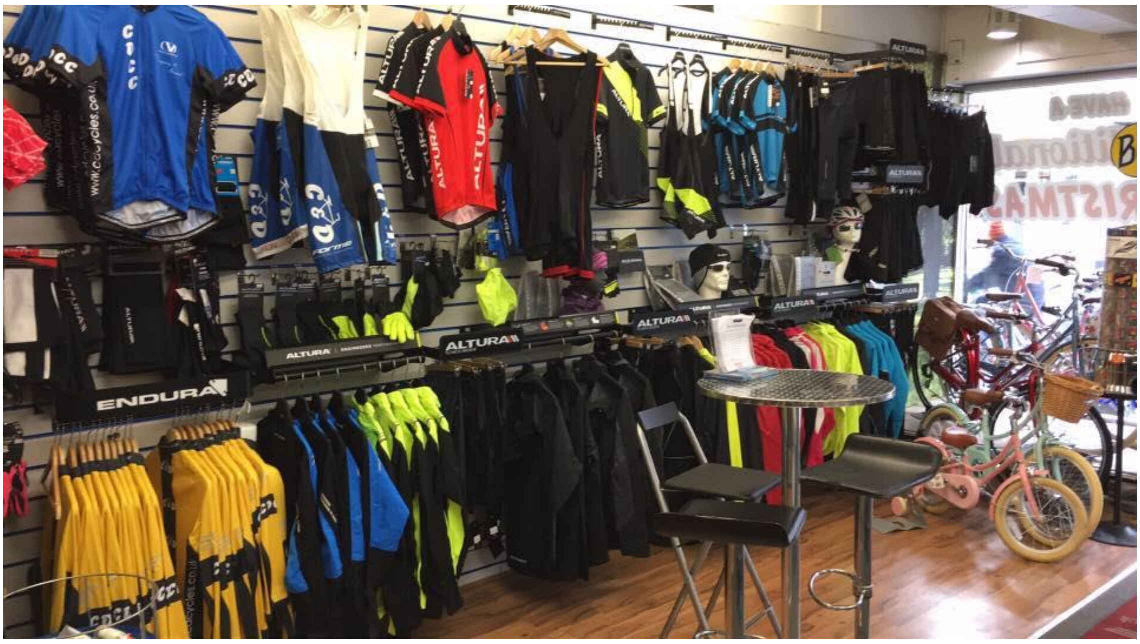

Display #68.   **Please evaluate this store from 1 to 10.**

1	2	3	4	5	6	7	8	9	10

Poorly displayed **Average** **Well displayed**

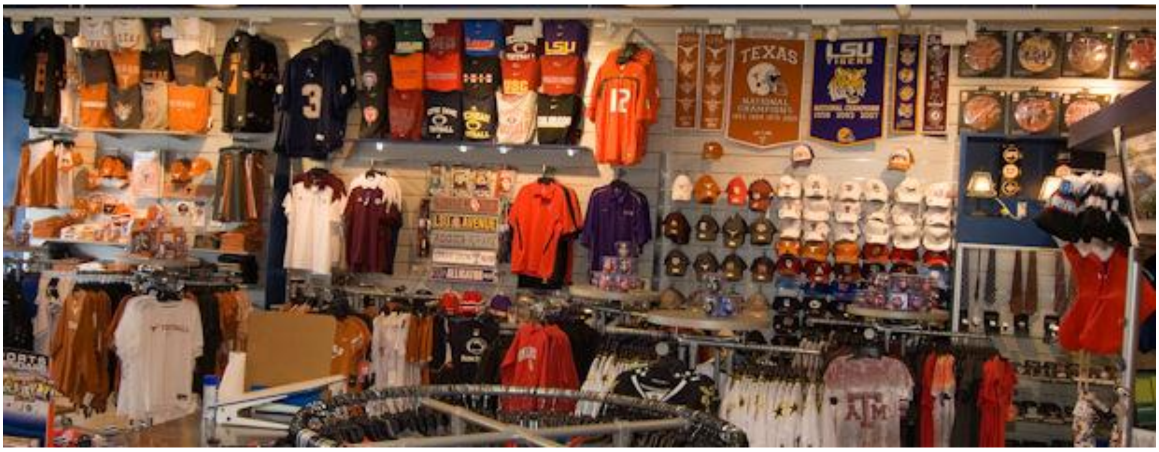

Display #69. Please evaluate this store from 1 to 10.

1	2	3	4	5	6	7	8	9	10

Poorly displayed **Average** **Well displayed**

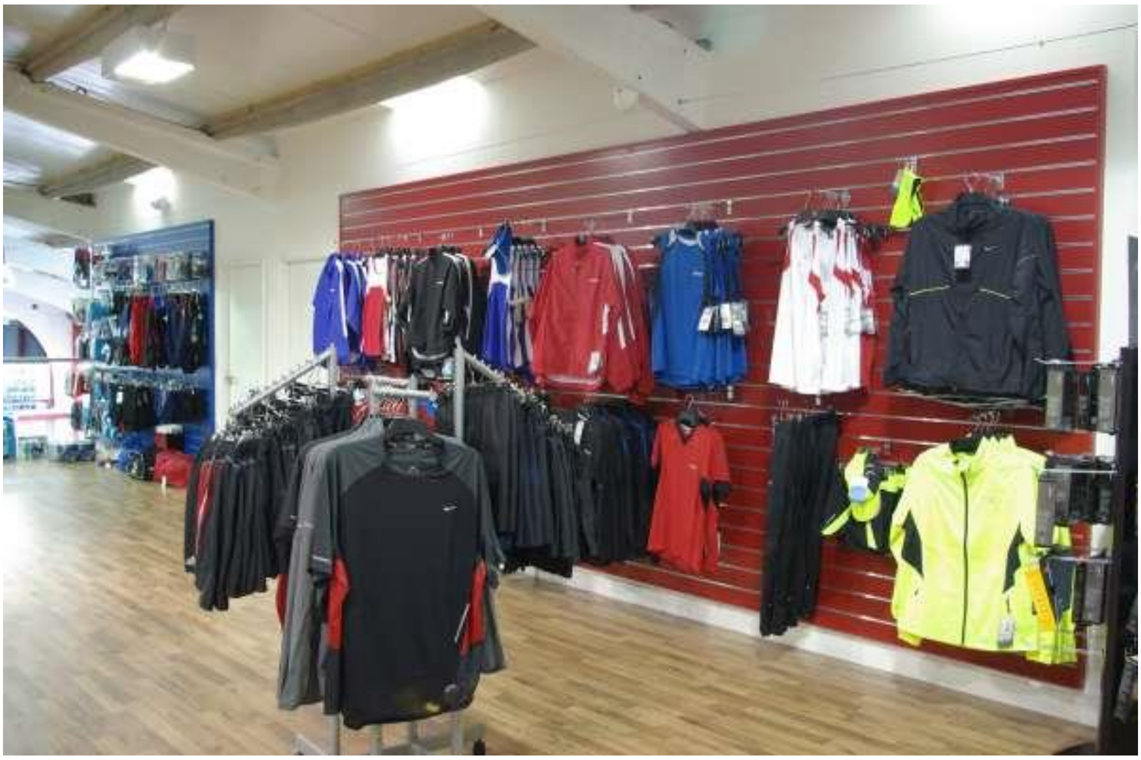

Display #70. Please evaluate this store from 1 to 10.

1	2	3	4	5	6	7	8	9	10

Poorly displayed **Average** **Well displayed**

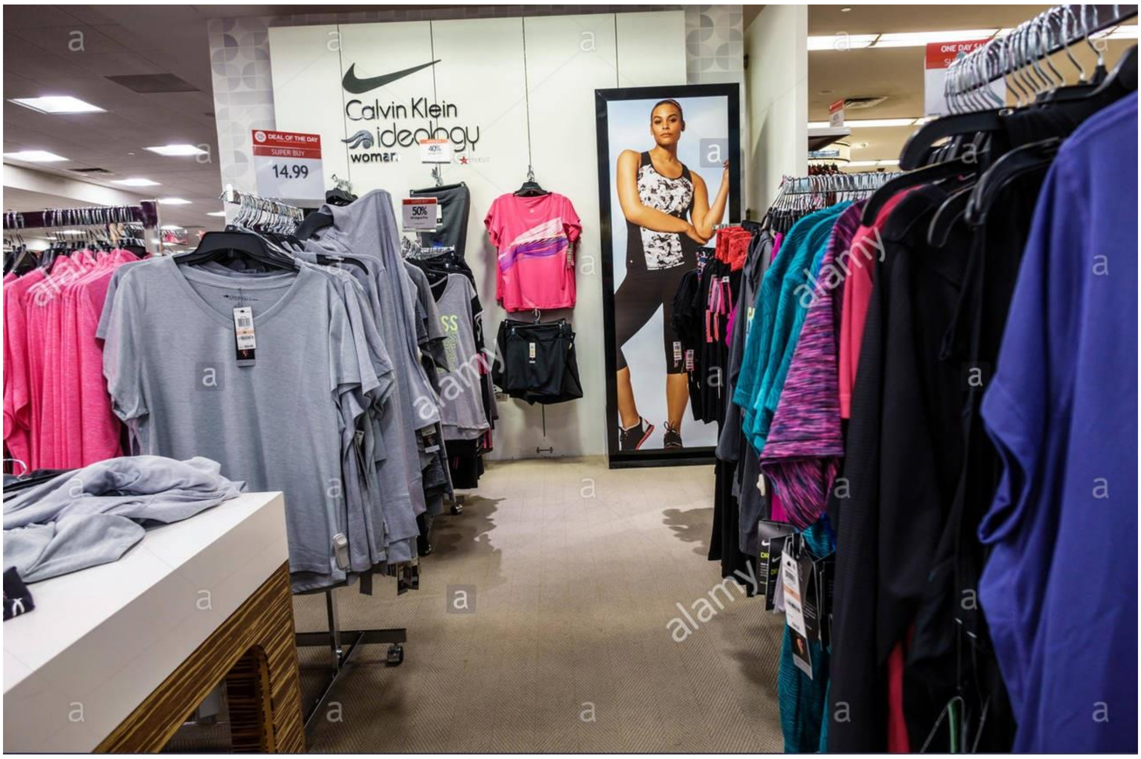

Display #71． Please evaluate this store from 1 to 10.

1	2	3	4	5	6	7	8	9	10

Poorly displayed **Average** **Well displayed**

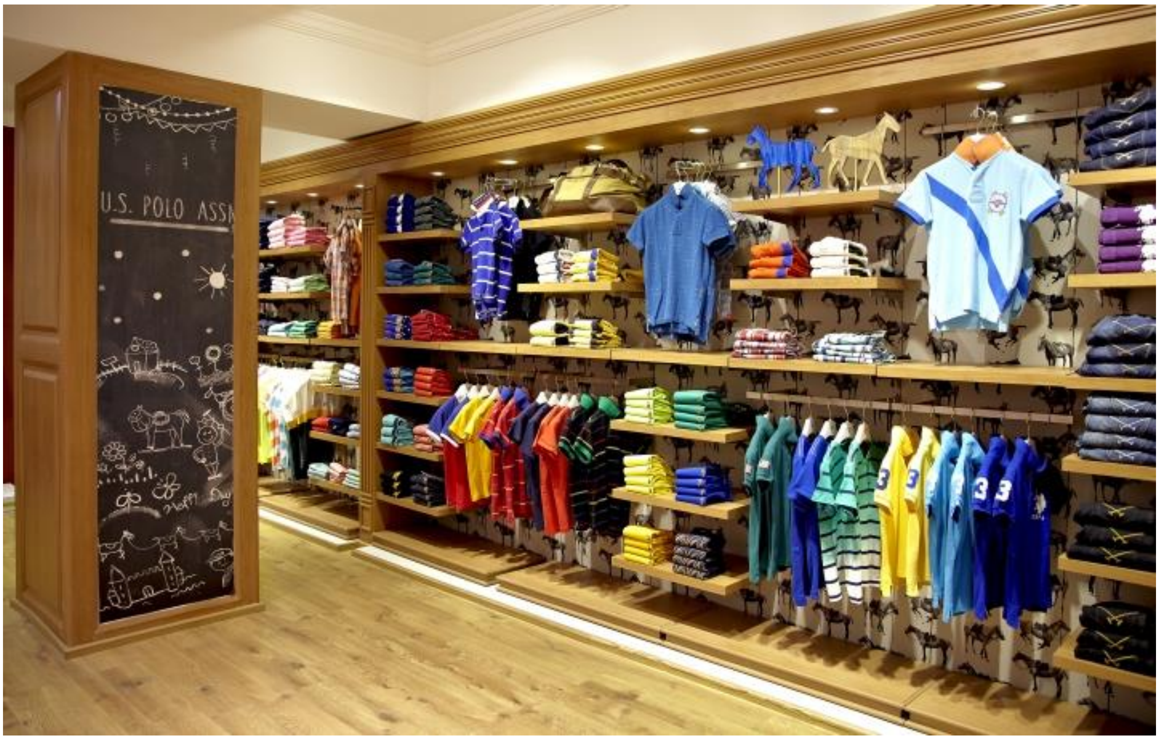

Display #72. Please evaluate this store from 1 to 10.

1	2	3	4	5	6	7	8	9	10

Poorly displayed **Average** **Well displayed**

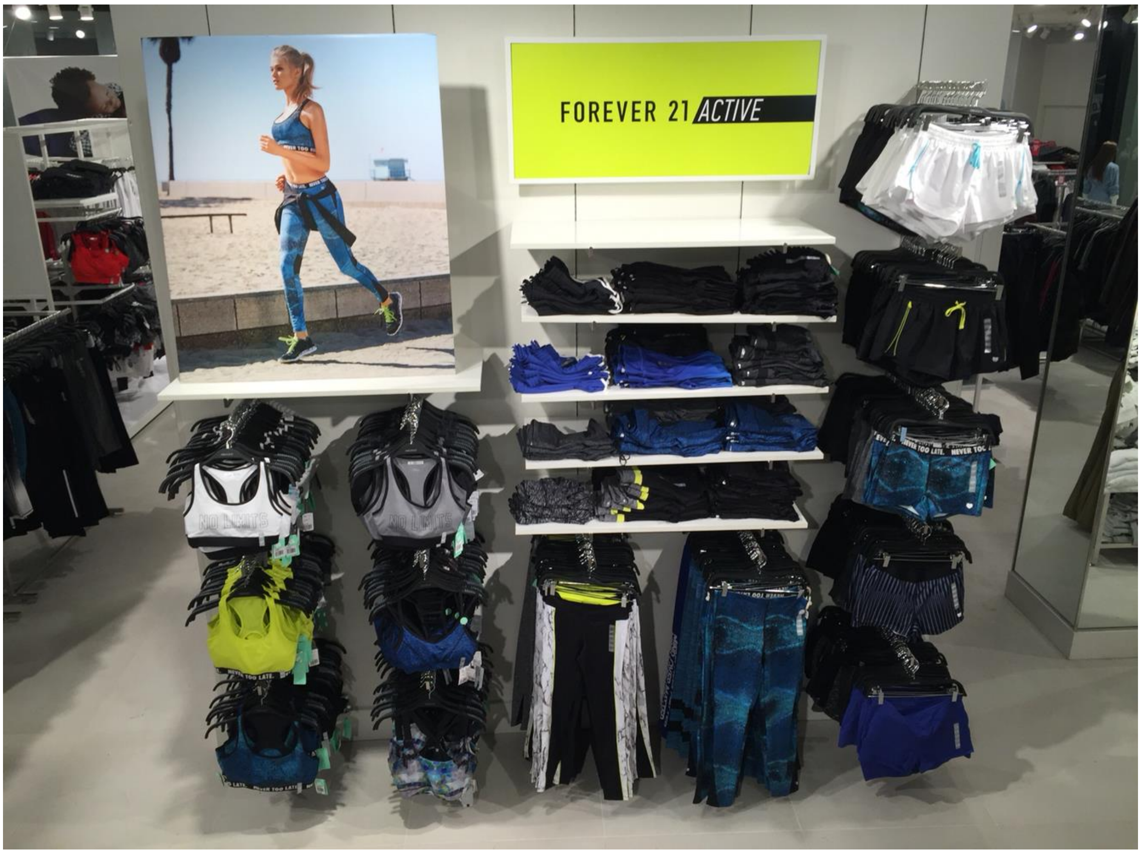

Display #73. Please evaluate this store from 1 to 10.

1	2	3	4	5	6	7	8	9	10

Poorly displayed **Average** **Well displayed**

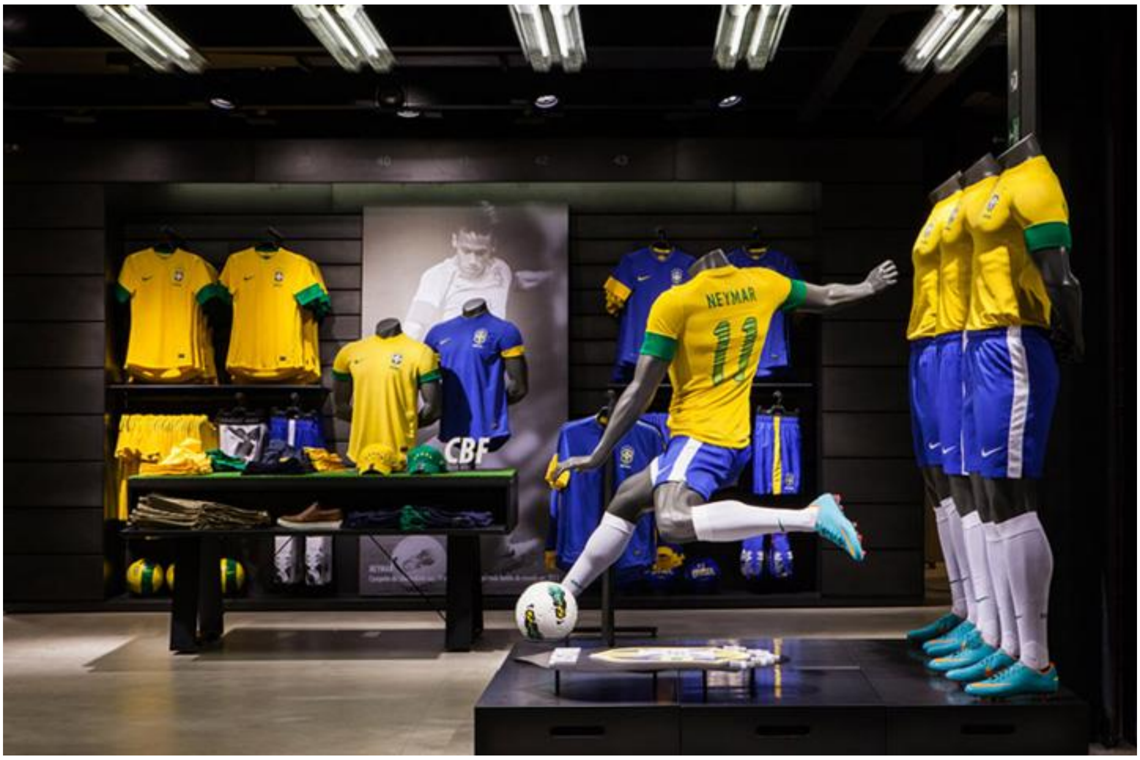

Display #74. Please evaluate this store from 1 to 10.

1	2	3	4	5	6	7	8	9	10

Poorly displayed **Average** **Well displayed**

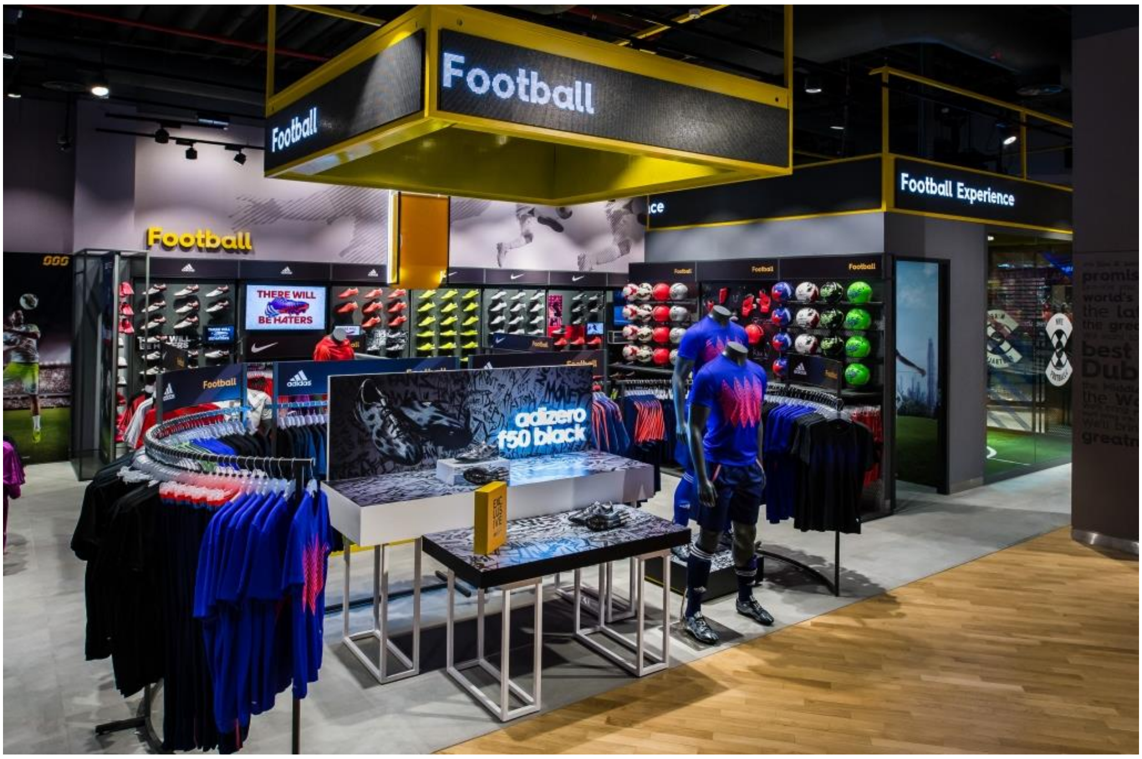

Display #75. Please evaluate this store from 1 to 10.

1	2	3	4	5	6	7	8	9	10

Poorly displayed **Average** **Well displayed**

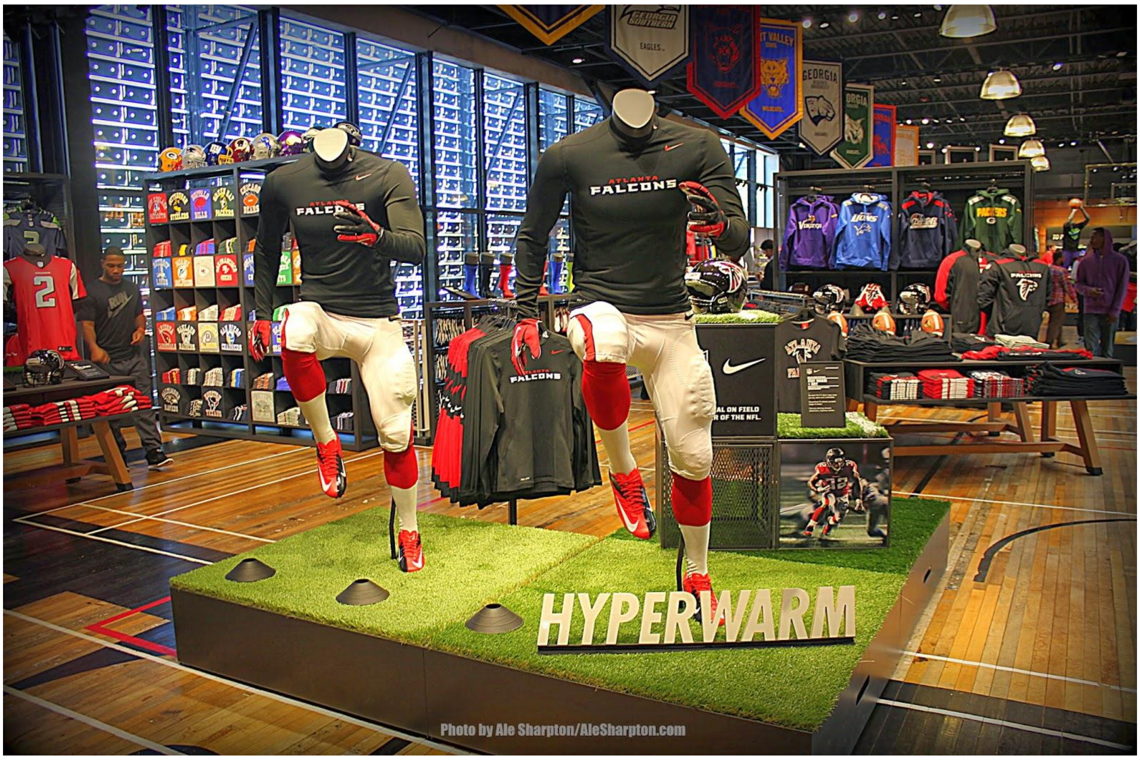

Display #76. Please evaluate this store from 1 to 10.

1	2	3	4	5	6	7	8	9	10

Poorly displayed **Average** **Well displayed**

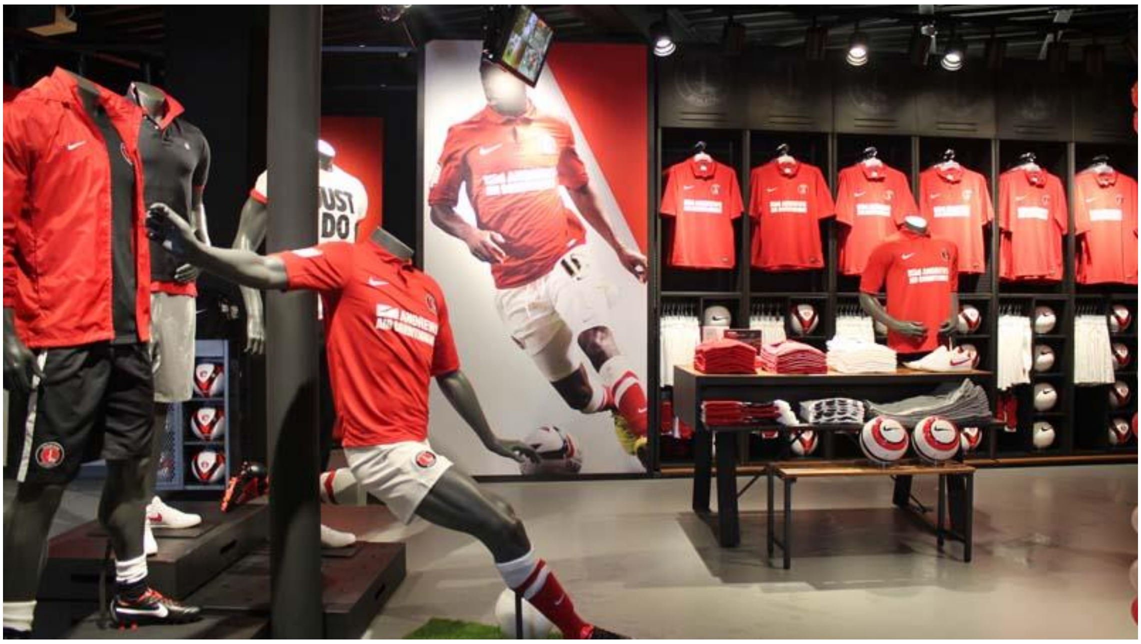

Display #77. Please evaluate this store from 1 to 10.

1	2	3	4	5	6	7	8	9	10

Poorly displayed **Average** **Well displayed**

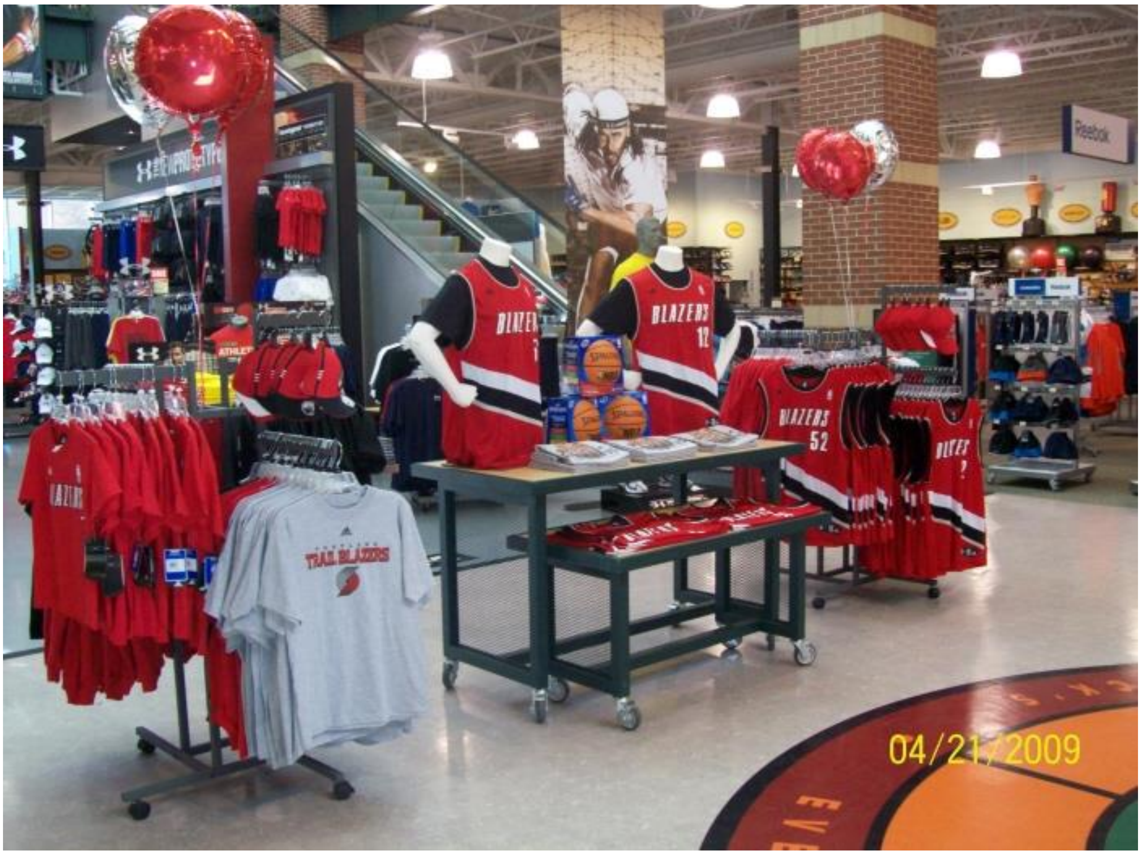

Display #78.   Please evaluate this store from 1 to 10.

1	2	3	4	5	6	7	8	9	10

Poorly displayed **Average** **Well displayed**

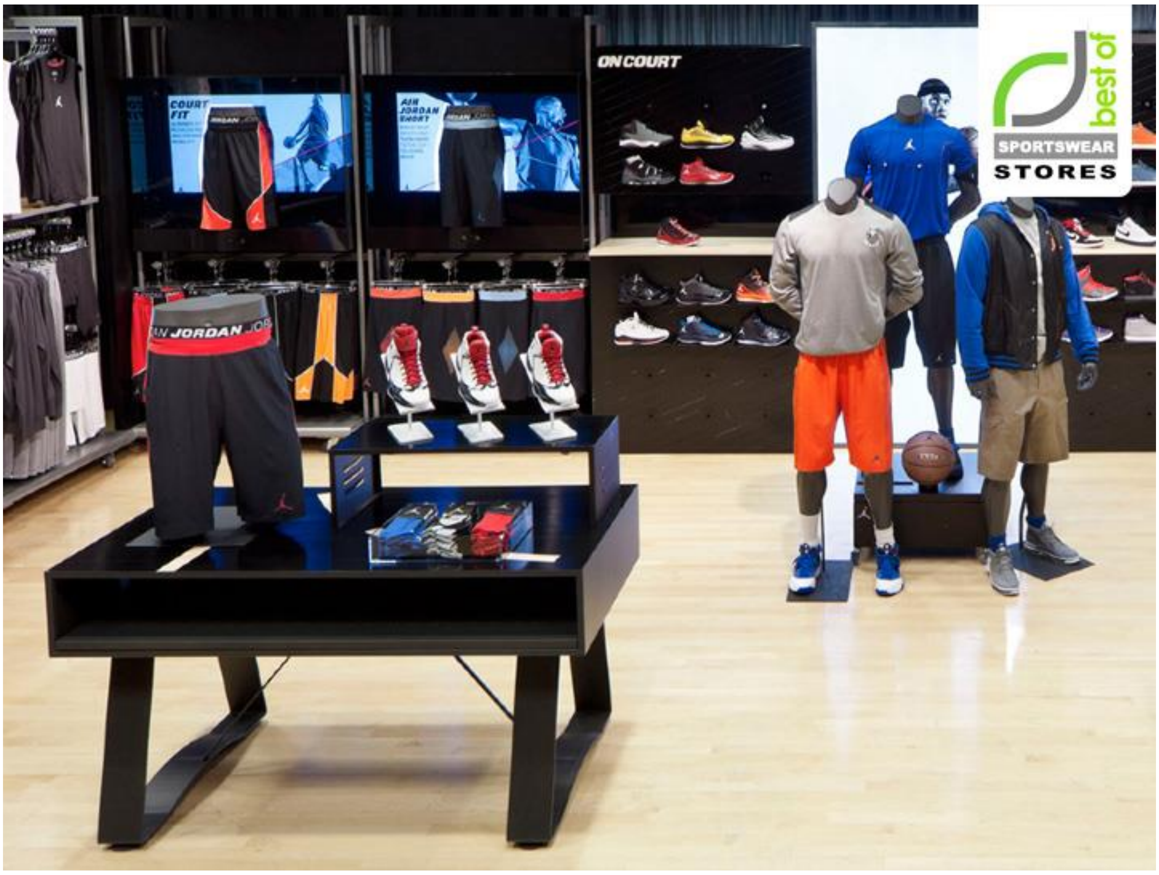

Display #79. Please evaluate this store from 1 to 10.

1	2	3	4	5	6	7	8	9	10

Poorly displayed **Average** **Well displayed**

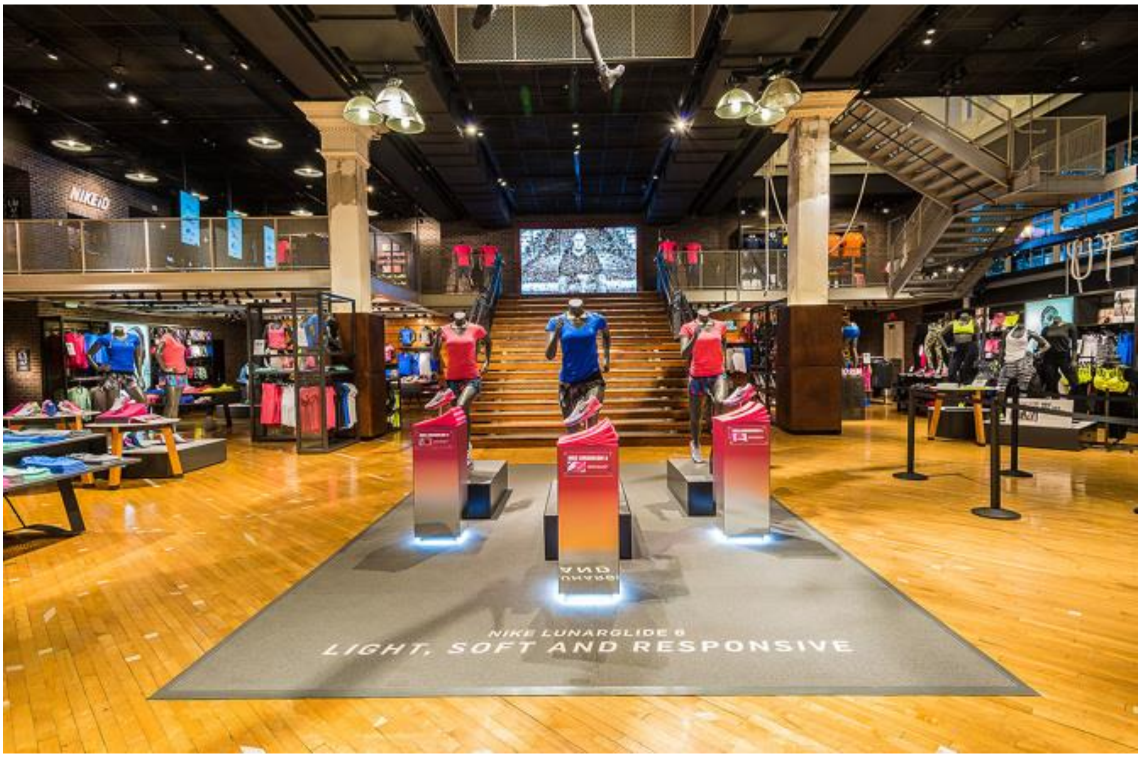

Display #80. Please evaluate this store from 1 to 10.

1	2	3	4	5	6	7	8	9	10

Poorly displayed **Average** **Well displayed**

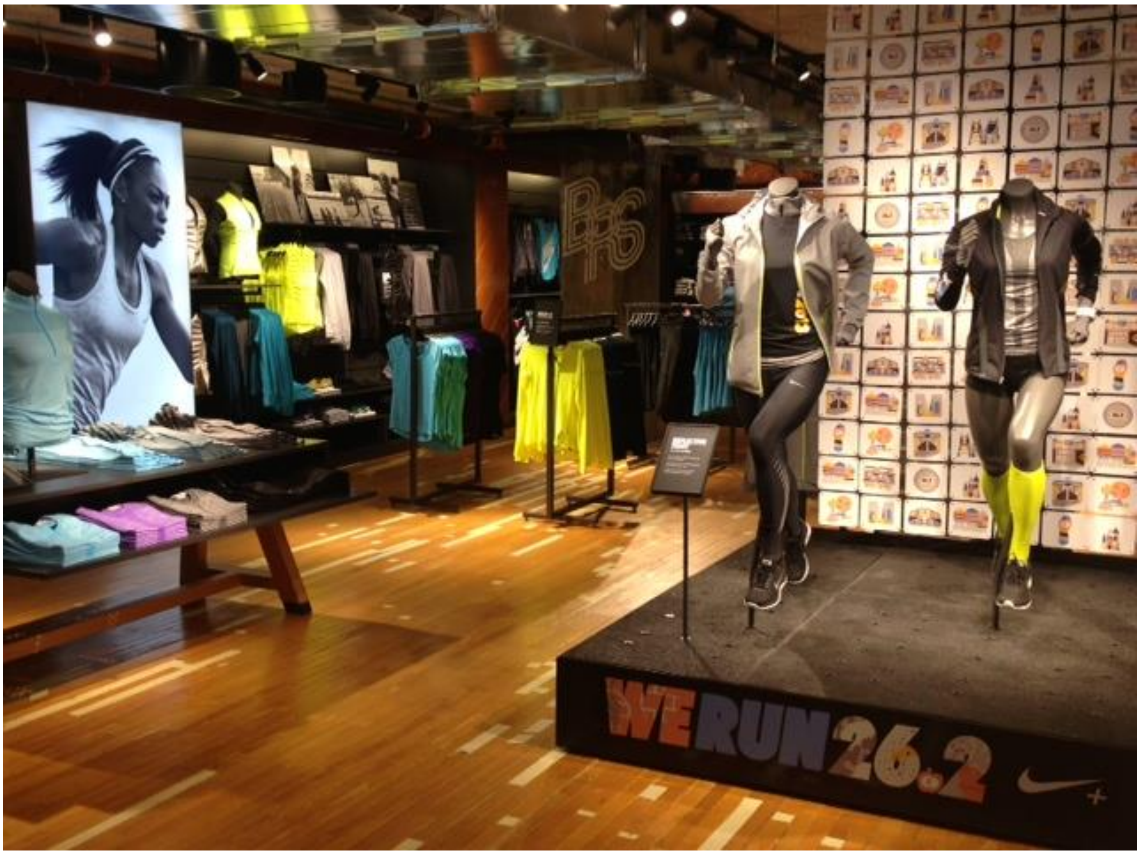

Thanks for your participant.

谢谢您的参与
